
JOURNAL INFORMATION
==============================
NLM Title Abbreviation: Innov Aging
Journal ID: Innov Aging
NLM Journal ID: 101703706
Journal ID: innovateage

Innovation in Aging

EISSN: 2399-5300
Publisher: Oxford University Press

ARTICLE INFORMATION
==============================
PMCID: PMC9995573
PMID: 36910698
DOI: 10.1093/geroni/igac065
Article ID: igac065
Article version: 1
Subjects: Participant Index, AcademicSubjects/SOC02600

Participant Index

Publication date: 2022 Nov
Electronic publication date: 2022 Dec 20
Volume: 6
Issue: Suppl 1
Issue title: Program Abstracts from The GSA 2022 Annual Scientific Meeting, “Embracing Our Diversity. Enriching Our Discovery. Reimagining Aging.”
First page: 882
Last page: 919
Copyright: © The Author(s) 2022. Published by Oxford University Press on behalf of The Gerontological Society of America.
Copyright year: 2022
License: This is an Open Access article distributed under the terms of the Creative Commons Attribution License (https://creativecommons.org/licenses/by/4.0/), which permits unrestricted reuse, distribution, and reproduction in any medium, provided the original work is properly cited.
License URL: https://creativecommons.org/licenses/by/4.0/

Page count: 38

==============================

### Participant Index

Arabic numbers refer to session numbers.
Duplicate numbers indicate the participant is listed as an author on multiple abstracts within a session.

**A**
Aartsen, Marja J.: 2410
Abadir, Peter M.: 6411
Abah, Theresa L.: 6350
Abbasi, Khuzeman: 2510, 6240
Abbate, Lauren M.: 4260
Abbott, Katherine M.: 2340, 3180, 3520, 4270, 4360, 4610, 6130
Abboud, Anissa: 3360
Abe, Koji: 6210
Abenojar, John Paul T.: 3950
Abe, Takumi: 6320, 6710
Abraham, Sitara J.: 4150
Abrams, Leah: 4110
Abshire Saylor, Martha: 3330
Abudu-Birresborn, Diana: 1420
Abujarad, Fuad: 5100
Acevedo-Fontanez, Adrianna I.: 6450
Achanta, Madhuri: 9010
Acharya, Geeta: 3690
Acosta, Lealani Mae: 4461
Acton, Gayle: 9020
Adamek, Margaret E.: 6330, 6630, 6800
Adamsen, Collette: 6180, 6280
Adams, Mariah: 6270
Adams, Mary Anne: 1400
Adams, Peter: 1200
Adams, Michaela: 6210
Adams-Price, Carolyn: 3380, 6280, 6790
Adams-Price, Carolyn E.: 4660
Adaralegbe, Adeleye A.: 2510, 6240
Addington, Elizabeth: 2300
Addison, Odessa: 5050
Adebusoye, Lawrence A.: 3900
Adekunbi, Daniel: 5001, 6010, 9010
Adelman, Ronald: 6530, 9030
Adelnia, Fatemeh: 4461
Adena, Maja: 2410
Adeniji, Dolapo O.: 6790, 6800
Adepoju, Omolola E.: 3540
Adesanya, Ikmat: 9020
Adeyombo, Adewale A.: 6500
Adhikari, Bandita: 6010
Adhikari, Dristi: 9020
Adinkrah, Edward: 9020
Adjei Gyimah, Akwai: 4684
Adler-Milstein, Julia: 1510, 6270
Adler, Nancy: 3070
Adrian, Shayle: 1250
Afaya, Agani: 6320
Aflatoony, Leila: 4030
Afsar Ali, Afnaan F.: 1570
Agans, Gretchen: 6760
Agarwal, Arunika: 4690
Agerholm, Janne: 6200
Aggarwal, Neelum: 6160
Agrawal, Yuri: 6740
Agrimson, Kellie S.: 9010
Agronin, Marc: 9000, 9010
Aguilan, Jennifer: 1200
Aguilar, Iris: 6770
Aguilar, Kaysey: 6710
Aguirre, Annabella S.: 2290
Agus, Sam: 9010
Agyemang, Lillian: 6390
Ahles, Tim A.: 1100, 3400
Ahluwalia, Jasjit: 9000
Ahmadi, Soheila: 9000
Ahmad, Omar: 3750
Ahmad, Waqar: 6760
Ahmed, Ali: 5173
Ahmed, Courtney: 3000
Ahmetovic, Alma: 6770
Ahn, Hyochol: 4490
Ahn, Roy: 4240
Ahn, Sangwoo: 9000, 9020
Ahrens, Monica L.: 9000
Ai, Amy: 9030
Aichele, Stephen R.: 3115, 3250
Aiello, Allison E.: 3430, 6050
Aiken Morgan, Adrienne T.: 2380
Aikens, James: 3130, 6060
Ailshire, Jennifer A.: 1410, 4140, 4690, 5000, 5130, 6310, 6710, 6830, 9000
Aitken, Sarah: 2090, 6280
Aizenstein, Howard: 1050
Ajith, Aniruddh: 9030
Ajrouch, Kristine J.: 1240, 3430, 5170
Akahane, Manabu: 2580
Aker, Narin: 3555
Akincigil, Ayse: 1170
Akishita, Masahiro: 6240
Akksilp, Katika: 9030
Akosah, Kwame O.: 1480
Akosionu, Odichinma: 3910
Akre, Ellesse-Roselee: 1320
Akushevich, Igor: 1000, 1390, 3720, 4290, 6060, 6110, 6411, 9000
Al Snih, Soham: 6300
Alagappan, Cecilia: 9000
Alamer, Khalid A.: 6510
Alam, Md Towfiqul: 2170
Alaniz, Amber: 4830
Alappan, Narendrakumar: 6270
Alarid-Escudero, Fernando: 6230
Albala, Cecilia: 6320
Albarran, Marz: 3000
Albers, Elizabeth A.: 1480, 4060
Albert, Jeffrey: 6200
Albert, Steven M.: 2330, 3880, 6130
Albrecht, Jennifer: 6740
Alcantar, Sandy: 3750
Alcorn, Edwin: 6420
Alderson, Jacob E.: 2140, 4620, 6550, 9010
AlDossary, Heba: 1530
Aldridge, Melissa D.: 5030
Aldwin, Carolyn M.: 2470, 5140, 6210, 9020
Alexander, Anne: 3460
Alexander, Bruce: 6100
Alexander, Gregory L.: 1510
Alexander, Karah: 4030
Alexander-Miller, Martha A.: 6100
Alford, Susan: 9000
Alireza, Salami: 3590
Ali, Sumbleen: 1290
Ali, Talha: 3360
Ali, Zuha: 9020
Alladi, Renuka: 9030
Allaire, Jason C.: 2380, 3270
Allala, Sai Chaithra: 9000
Allan, Alexa C.: 2380
Allemand, Mathias: 4620
Allen, Julie Ober: 4290
Allen, Katy: 9030
Allen, Laura D.: 1400
Allen, Lauren: 6210
Allen, Rebecca S.: 2030, 3251, 6100, 9020
Allen, Tammy D.: 2620, 6600
Allgood, Sallie: 4360
Allison, Matthew: 4330
Allison, Theresa A.: 3050
Allore, Heather G.: 3110, 6340, 6450, 6740, 9010
Allsopp, Richard: 3580, 6010
Almada, Luciana: 9000
Almeida, David M.: 2430, 2470, 2620, 3730, 3770, 4080, 5060, 6260, 6580, 6600
AlObaidi, Alya: 6641
Alomairi, Salhah A.: 9030
Alpass, Fiona: 3900
Alperin, Kate: 9020
Alpert, Jordan: 6510
Alshaheri, Alin: 9000
Alshakhs, Sulaiman: 9030
Alsulaily, Amjed: 4100
Altukhys, Abdulmajeed: 6420
Alvarado, Angela: 1310
Alvarez, Jessica: 9000, 9010
Alvarez, Valerie: 1310
Alves de Lima, Khalil: 4461
Alwan, Elizabeth: 6210
Amano, Takashi: 5130, 6060, 6800, 9000, 9020
Ambrose, Madison: 5172
Amestoy, Maya E.: 6440
Amick, K. Allison: 4461, 6010
Amick, Matthew E.: 6790
Amini, Reza: 6220
Amos, Christopher: 9020
Amstrong, Naomi: 6130
Ananthasubramaniam, Aparna: 4630

© The Author(s) 2022. Published by Oxford University Press on behalf of The Gerontological Society of America.

Anastasia, Trena: 3460
Andel, Ross: 2620, 4080, 4465, 6290, 6600
Andersen, Julie: 6840
Andersen, Stacy: 2501, 4810, 4870, 9030
Anderson, Andrea: 9000
Anderson, Brianna L.: 6240
Anderson, Hannah L.: 3520
Anderson, Joel: 2390, 9020
Anderson, Jon: 2060
Anderson, Katie: 6411
Anderson, Keith A.: 3000, 9030
Anderson, Loretta: 1030, 3140
Anderson, Nicole: 6380
Anderson, Rozalyn: 1140, 2180, 2330
Anderson, Ruth A.: 1540, 4210, 6760
Anderson, Sharon D.: 1420
Andes, Laura: 2690
Andrade, Flavia: 4370, 6650, 9020
Andrasfay, Theresa: 3260, 6710
Andreoletti, Carrie: 1070, 3800
Andreo, Micaela: 9020
Andrew, Nyche T.: 4710
Andrews, Claire: 6770
Anetzberger, Georgia J.: 3920, 4440, 4830
Angel, Barbara: 6320
Angelini, Luise: 2501, 6840
Angel, Jacqueline L.: 3020, 3240
Ang, Gregory: 3620, 4220
Ang, Shannon: 6710
Anilkumar Hemalatha, Sruthi: 2150, 6610
An, Jaesung: 4190, 6170
Ankuda, Claire K.: 5070
Anstey, Kaarin J.: 4022, 9000
Antczak, Radoslaw: 4690
Antic, Mina: 2620
Anton, Greta: 9030
Antonucci, Toni C.: 1240, 1290, 2440, 3430, 5170
An, Yang: 6740
Apostolova, Liana G.: 3570, 6820
Applebaum, Robert: 1530, 3180, 9010
Appleby, James: 4400
Apsley, Abner T.: 5060
Aranda, Maria P.: 2420, 4420, 6180, 6770
Arbeev, Konstantin: 1000, 1390, 4290, 4810, 6010, 6110, 6411, 9000
Arbex Silva Borim, Flávia: 9020
Ardakani, Azam: 6210
Aree-Ue, suparb: 3840
Arellano, Victor: 9010
Arevalo, Lyda: 9000
Argawal, Shailesh: 5001
Arguello, Eric: 6780
Arias-Merino, Elva Dolores: 6290, 6380
Arieli, Rotem: 4140
Arisoyin, Abimbola E.: 2510, 6240
Arling, Gregory: 9030
Armah, Henrietta Q.: 6590
Armao, Anne: 9010
Armstrong, Peyton A.: 9000
Arnold, Matthew: 1140
Arnold, Robin: 6220
Arora, Kanika: 2245
Arpawong, Thalida: 5140
Arpino, Bruno: 4690
Arrasmith, Chloe: 2340
Arroyo-Miranda, Mirna L.: 1440
Arshad, Faaizah: 3560
Ars, Joan: 6450
Arthur, Christopher B.: 9000
Aryal, Subhash: 6510, 6600
Asana, Ventra: 3860
Asante, Samuel: 6350
Asghari, Mehran: 6510
Ashby, Joan L.: 6110
Ashcraft, Alyce S.: 6760
Ashebir, Sophia: 3260, 4090
Ashe, Maureen C.: 3150, 6580
Ashida, Sato: 3240, 6830
Ashirifi, Gifty: 6790, 6800
Asquith-Heinz, Zayla: 3790, 4710
Assi, Lama: 6710
Astell, Arlene: 2460, 6380
Atherton, Olivia E.: 2620
Atkins, Ghislaine C.: 6750
Atkinson, Elizabeth: 3241
Atkinson, Taylor J.: 6290
Auerbach, Samantha: 6510
Ausserhofer, Dietmar: 1530
Austad, Steven N.: 2501, 3001, 4410
Austin, Thomas R.: 3400
Au-Yeung, Wan-Tai M.: 6080
Avent, Elizabeth S.: 3010
Aversa, Zaira: 3241
Avila, Jaqueline C.: 9000
Avila-Rieger, Justina: 2270
Avni, Elinore: 1450
Avtzi, Stella: 6450
Awata, shuichi: 3010
Ayabe, Takako: 9010
Ayalon, Liat: 1400
Ayasoufi, Katayoun: 6010
Ayele, Roman: 6210
Ayers, Emmeline: 6780, 9020
Azar, Martina: 6260
Azer, Lilian: 4691
Azhar, Gohar: 6130, 6180, 6840
Azizi, Mehrnoosh: 9000
Azman, Nur Diyana: 2150
Azuero, Andres: 6390

**B**

Bédard, Michel: 6170
Béland, François: 2580
Bülow, Pia H.: 9010
Baar, Tena: 9020
Baatar, Anujin: 4684
Babitzke, Jennifer: 2720
Bacsu, Juanita-Dawne R.: 9000
Badawi, Sarah: 6140
Bae, Harold: 4810, 6010
Baehr, Laura: 1120
Baek, Jihye: 4370, 5150, 9000, 9010, 9020
Baek, Jiwon: 6400
Baer-Benson, Henry: 3390
Baggett, Sharon: 1190
Bagley, Olivia: 1390, 6411
Baier, Rosa R.: 3310
Baik, Dawon: 6280
Baik, Sol: 4510, 9030
Bailey, Amelia: 9030
Bailey, Dionne J.: 6360
Baillargeon, Emma M.: 4340
Baim-Lance, Abigail: 1160, 3660, 6320, 6610, 9000
Baird, Lola: 2660, 6220
Baird, Sean: 6390
Bair, Heather: 2520
Bai, Xue: 1400
Bai, Yi-dong: 2501
Bakeman, Roger: 2520
Baker, Elaina: 5070, 6310
Baker, Emma: 4240
Baker, Hannah: 3483
Baker, Julia: 2340
Baker, Tamara: 2360, 3420, 6180
Baker, Zachary G.: 2730, 3310, 6070
Bakker, Franka C.: 4550
Bakk, Louanne: 6390, 9030
Bakulski, Kelly M.: 3430
Balaswamy, Shantha: 6090
Balbim, Guilherme M.: 6570
Baldwin, Megan: 3360
Bali, Krittika: 6770
Balingit, Arianna R.: 9010
Ballard, Aimee: 9000
Ballard, Erin E.: 6590
Ballew, Shoshana A.: 4330
Ball, Karlene: 9020
Balmuth, Alexa: 3260, 6310
Balubaid, Afnan: 6280
Banchereau, Jacques: 3001
Bandeen-Roche, Karen: 1010, 2200, 3110
Band-Winterstein, Tova: 9030
Banerjee, Sreya: 4190
Banerjee, Subhranshu: 6290
Bangerter, Lauren R.: 2160, 6110, 6530
Baniassadi, Amir: 9030
Baniya, Ganesh: 6690
Bankole, Ayomide O.: 1160, 3482
Banks, Sarah: 6100
Banks, Sarah J.: 9000
Banner, Davina: 2210
Banwo Fatai, Khadijat: 6430
Barbakoff, Daniel: 6140
Barbeau, Sean: 3540
Barbee, Harry: 1320
Barber, David: 6150
Barber, Justin: 4870, 6720
Barber, Sarah J.: 3190, 6250
Barbieri, Steven: 4270
Barbour, Elizabeth: 6300
Barbut, Mariana: 6380
Barclay, Ruth: 6780, 9010
Barczi, Steven: 6420
Barger, Steven: 9030
Barinas-Mitchell, Emma: 4810
Barker, Anna: 5083
Barlow, Meaghan: 2620, 6580
Barmaki, Roghayeh: 4021
Barmon, Christina: 1070
Barnard, Ryan: 9000
Barnes, Christian: 9030
Barnes, Deborah E.: 3050
Barnes, Jessica T.: 3190
Barnes, Lisa L.: 2450, 3290, 3600, 6290
Barnes, Priscilla A.: 9020
Barnes, Stacy L.: 6280
Barnes, Timothy: 6580
Barnet, Jodi H.: 4560
Barnett, Michael D.: 6050, 6410
Barooah, Adrita: 2400, 6770, 9010, 9020
Barrón, Yolanda: 6100

Barragan, Cassandra: 6000
Barragan, Noel: 6060, 6220, 6280
Barrere, Cindy: 4681
Barr, Erik: 9020
Barreto, Manuela: 2690
Barrett, Anne: 4030, 6190, 6250, 6470, 6520
Barrett, Kendall: 2260, 6280
Barrett, Matthew: 6320
Barrett, Rod: 1280
Barry-Dutro, Erin: 9000
Barry, Lisa C.: 2510, 5120
Barry, Lisa: 3500
Barry, Teta: 9000
Bar, Sudipta: 6010, 6840
Barta, Hayley: 5001
Bartholomae, Suzanne: 4510
Bartle, Emily: 4020
Bartlett, Sara P.: 4750
Bartley, Emily J.: 2300
Bartley, Jenna M.: 9010
Bartley, Mairead: 6110
Bartoldus, Victoria: 5010
Bartone, Michael: 1070
Bartres-Faz, David: 3510
Barve, Shirish: 6290
Barzilai, Nir: 6010
Basaran Sahin, Duygu: 6640
Bashian, Hannah M.: 2030, 2660, 6220
Basic, Ajla: 6180
Basisty, Nathan: 9010
Basque, Shalane: 5000
Bass, Danica: 6050, 6410
Bass, David: 6530, 9000, 9020
Bass, Ellen J.: 5020
Bassett, Rachel: 9030
Bast, Elizabeth: 6420
Batsis, John A.: 2080, 4640, 6780
Battle, Brian: 2510
Baucco, Christina M.: 9000
Baucom, Brian: 1320, 2540
Bauer, Scott R.: 6320, 6780
Baughn, Maya: 9010
Bauldry, Shawn: 6810, 9020
Bauman, Alexandra: 9000
Baumann, Sara: 6130
Baumbach, Abby: 6750
Baum, Carolyn: 4790
Baumgart, Matthew: 1560, 2490
Bayani, Mario: 9000
Bayliss, Elizabeth: 3360
Bay, Maxwell: 9010
Bayne, Alycia: 3100, 4240
Bazargan, Mohsen: 9020
Beach, Brian: 3210
Beach, Douglas: 9020
Beach, Scott R.: 4090
Beachy, Tim: 5010
Beam, Christopher R.: 6290, 9000
Beamer, Brock: 6780
Bean, Jonathan: 5050
Beard, Renee L.: 9000
Beard, Sheriden: 3000
Bear, Todd M.: 2590
Beato, Morris: 6700
Beattie, Zachary: 2450
Beaulaurier, Richard: 6420
Beaulen, Audrey: 1160
Beaulieu, Celeste M.: 3800, 6000
Beauregard, Lisa: 1260
Beavers, Daniel: 3510
Beavers, Kristen: 6641, 9000
Beazley, Anthony: 6000
Becker, James T.: 1340
Becker, Melanie: 6380
Becker, Todd D.: 1390, 2700, 5150, 6720
Beck, Jennifer: 6010
Beck, Samuel: 3810
Beeber, Anna: 3170, 4210, 5010, 6450, 6760
Beech, Bettina M.: 6180
Behers, Benjamin J.: 9030
Behrens, Liza L.: 3520
Beigay, Teresa: 2590
Beilin, Lawrence: 4683, 5083
Beiser, Alexa S.: 9020
Belanger, Emma: 3200, 4820, 5081
Beld, Matthew: 9030
Belinka, Benjamin: 6411
Bellah, Sydney: 6620
Bellantoni, Michele: 1110
Bell, Bethany A.: 2300, 4730
Bellelli, Giuseppe: 5010
Bellen, Hugo: 6840
Belli, Hayley M.: 3060
Bellingtier, Jennifer A.: 2470, 2750
Bellizzi, Keith: 9000
Bell, Mallory: 6380
Bello, Ghalib: 1350
Bell, Sue Anne: 4465
Bell, Tyler R.: 3000, 4225, 6050, 6200, 9020
Belsky, Daniel: 3115, 5060
Beltran, Susanny J.: 6090, 6350, 6700, 9020
Belza, Basia: 5010
Benatti De Oliveira, Gabriela: 9020
Benayoun, Bérénice A.: 3001
Benda, Natalie C.: 9030
Benden, Mark: 6710
Bender, Alexis A.: 1170, 3760, 5173, 9000, 9010, 9020
Bender, Andrew R.: 4040
Bender, Anna E.: 1530
Bender, Emma N.: 6160
Bender, Kaye: 9030
Beneciuk, Jason: 9030
Benedetti, Kelli: 6411
Ben-Eliezer, Daniel: 6420
Benett, Jenil: 6220
Benjamin, Cynthia: 3050
Bennett, Crystal: 9000
Bennett, David A.: 2140, 4620
Bennett, Dayna S.: 3180
Bennett, Michael: 9030
Benson, Clark: 2160, 6310
Benson, Jacquelyn J.: 1300
Bentley, Megan: 4023
Benton, Donna: 4510, 6310
Benton, Melissa J.: 6770, 9010
Benton, Nathan: 3610, 6380
Benz, Hannah: 6140
Berbiche, Djamal: 6740
Berdai Chaouni, Saloua: 3610
Berdychevsky, Liza: 3260, 6390
Berg-Carramusa, Cara: 1280
Bergeman, Cindy S.: 4555, 6380
Bergen, Gwen: 4100
Berger, Corinne: 9000
Berger, Ann: 6280
Bergeron, Caroline: 2250, 6710
Berghoff, Ashley: 6280
Berg, Kristen: 4100
Bergmann, Johannes-Michael: 3090
Bergman, Sky: 4520, 4750
Bergman, Yoav S.: 3490, 6480
Bergstrom, Jaclyn: 4461, 6010, 9000
Bergstrom, Kelly A.: 3850
Berg-Weger, Marla: 1110, 6720, 6790, 9000
Berish, Diane: 1110, 2530, 3720, 9000
Berkman, Lisa F.: 3040
Berlinger, Nancy: 3585, 4380
Berman, Rebecca: 4270, 4590
Bernacchi, Veronica: 9010, 9020
Berna, Renee: 6560
Bernays, Florence: 5100
Berndt, Annette: 9000
Bern-Klug, Mercedes E.: 4830
Bernstein, Laura: 9000, 9020
Berridge, Clara: 3670, 6260
Berry, Sarah: 2290
Berry-Stoelzle, Maresi: 6100
Berta, Whitney: 4210
Bertoni, Alain: 3400
Bertram, Lars: 1010, 6010, 6641
Besnier, Florent: 6570
Besser, Lilah: 1050
Bessette, Lily G.: 9030
Bessette, Lily G: 6641
Best, Irene: 2230
Best, John R.: 6290
Best, Ryan: 6440
Bethea, Traci N.: 3400
Bethell, Jennifer: 6270, 9000
Betley, Wendy: 6280
Betrisey, Carine: 6140
Betts Adams, Kathryn: 6250
Betzel, Richard: 6290
Betz, Joshua: 3560
Betz, Marian E.: 6470, 9030
Beverly, Alexis R.: 9000
Beydoun, Safa: 6010
Bhagianadh, Divya: 2245
Bharadwaj, Mythili: 6000
Bhat, Aarti: 3770, 5060, 6580
Bhat, Ajay: 6010
Bhattacharjya, Sutanuka: 4160
Bhattacharyya, Kallol Kumar: 4465, 6540
Bhatta, Tirth: 4905, 6170, 6200
Bhatti, Danish: 6420
Bhatti, Usama: 2160
Bhatt, Jem: 3610
Bhatt, Tanvi: 4800, 6420, 6570, 6780
Bherer, Louis: 6570, 6740
Bianchi, Andria: 9000
Bian, Wenxin: 9000
Bibbo, Jessica: 1190, 3530
Bielak, Allison A M: 1100
Bierman, Alex: 4022
Biery, Nyann: 9020
Biese, Kevin: 4390
Biesmans, Jesper: 6270
Biggar, Virginia S.: 2160
Bigger, Sharon: 9020
Biggs, Mary L.: 3400
Bijou, Christina: 4225, 6200
Bilbrey, Ann Choryan: 6750

Bildtgård, Torbjörn M.: 1300
Billings, Pamela: 6360
Binford, Sasha: 6460
Birditt, Kira: 1450, 2440, 3480, 3730
Biritwum, Akosua B.: 9000
Birkeland, Robyn W.: 4220
Bisconti, Toni L.: 4730, 9000
Bishnoi, Alka: 6740
Bishop, Alena: 9000
Bishop, Alex: 6180, 6620, 6810
Bishop, Christine E.: 6360, 6540
Bishop, Nicholas J.: 1090
Bitto, Alessandro: 6840
Biyani, Rajlaxmi M.: 6790
Bjore, Emma: 3460
Bjork, Lara: 3100
Blackberry, Irene D.: 3550
Blackburn, Justin: 1190, 4100, 6760
Black, Kathy: 4465
Blackshear, Chad: 3110, 4200
Blackwell, Terri: 9010, 9030
Blackwood, Jennifer: 6220, 6450, 6510, 9030
Blair, Elizabeth: 2020
Blanche, Erna I.: 6740
Blanc, Judite: 6680
Blaskewicz Boron, Julie: 2140, 6090, 6170, 6290, 6390
Bleijlevens, Michel H.: 5100, 6270, 6560
Blench, Karl: 3481
Blieszner, Rosemary: 3240, 9020
Blinka, Marcela D.: 2690
Blocker, Kenneth A.: 3410, 4160, 9020
Block, Laura: 2160, 3750, 9010
Bloom, David E.: 4690
Bluck, Susan: 4555, 6300, 6340, 6380
Blumen, Helena: 6780
Boafo, Jonelle: 3060
Boakye, Maxwell: 6110
Bobitt, Julie: 2245, 5082, 5130, 9020
Boblitz, Alexa: 9000
Bobo, Michelle: 6740
Bobroff, Stella: 9000
Bochnak, Paula: 3620
Bodenheimer, Lisa: 6560
Boelen, Paul A.: 4460
Boerner, Kathrin: 1320, 2440, 2720, 4460, 6480, 6830
Boger, Jennifer: 2460
Bohnert, Adam: 2501, 6840
Bohplian, Suweena: 6780
Bokka, Anirudh: 6411
Bokun, Anna: 6800
Boland, Angela K.: 6290
Boland, Elizabeth M.: 6470
Bolkan, Cory: 4900, 6610
Bollens-Lund, Evan: 4530, 5030
Bolton, Cherish A.: 6120
Bolt, Sascha R.: 2090, 3555, 6270
Boltz, Marie: 2230, 2530, 2680, 3310, 3520, 4300, 4760, 6420, 9030
Bonaguidi, Michael: 9010
Bonazinga, Lindsay: 3460
Bonds Johnson, Kalisha: 1440, 3240, 4610, 4850
Bonewald, Lynda: 5001
Bonifas, Robin P.: 6060
Bonner, Gloria: 6160
Bonomo, Melia: 3481
Bons, Joanna: 3241
Boockvar, Kenneth S.: 5030
Boot, Walter R.: 1570, 3410, 3670, 4020, 4190, 6290
Borasio, Gian Domenico: 2680, 3720, 4480, 6730
Boraxbekk, Carl-johan: 9010
Borcar, Apruva: 6641
Bordes, Piper J.: 6830
Borek, Pamela: 4150
Boren, Hannah: 6270
Borgmann, Lisa: 2750
Boring, Michael A.: 4120
Borkan, Jeffrey: 6770
Boron, Julie B.: 2140
Boroumand, Shahdokht: 6230
Borrat-Besson, Carmen: 2680
Borre, Ethan D.: 9030
Borrero, Lisa: 2350, 3780, 9030
Borson, Soo: 1150
Bos, Angelo Jose G.: 3620
Boscardin, W. John: 2510, 3690, 4460, 5030
Boscart, Veronique: 6270
Bosch, Pamela: 9030
Bosse, Jordon D.: 5084
Boström, Anne-Marie: 6760
Botoseneanu, Anda: 6290, 6340, 6450, 6740
Botsch, Meghan: 2160
Bouchard, Elizabeth G.: 6430
Bouchard, Lauren M.: 6580
Boudreau, Jacqueline H.: 1040
Boudreau, Robert M.: 9030
Bouldin, Erin D.: 1330, 3780, 6280, 6310, 6530
Bourassa, Kyle: 5050
Boustani, Malaz A.: 6580, 9000
Bowblis, John: 3180, 3520, 3950, 6540
Bo, Wei: 6280
Bowen, Lauren M.: 3800, 6000
Bowen, Mary (Libbey) E.: 6760
Bowen, Pamela G.: 6750
Bowers, Katherine: 3140, 9020
Bowles, Kathryn H.: 6100
Boxer, Rebecca: 3360
Box, Helen: 6280
Boyd, Cynthia: 2690, 6800
Boyd, Kelly: 6220
Boyko, Edward: 3400
Boyle, Joseph: 6070
Boyle, Julia T.: 2030
Brabham, Deborah D.: 1420
Bradley, Dana B.: 1250, 1400, 3420, 6790
Bradshaw, Spencer: 6410
Brailsford, Jennifer: 9030
Brandt, Anthony: 3481
Brandt, Cynthia: 6770
Brandt, Martina: 6300
Brandt, Nicole: 3650, 6350, 6560
Brasher, Melanie: 6400
Brauer, Simon G.: 1240
Braun, Kathryn L.: 3220, 6180
Bravata, Dawn M.: 6390
Bravo-Ramirez, Karen: 9020
Bravo, Rosana: 6140
Breda, Kathleen M.: 5083
Breitfelder, Laura K.: 1020
Brekken, Rolf: 9000
Brem, Rachel: 6010
Brennan-Ing, Mark: 3760
Brenner, Rachel: 1330
Brenowitz, Willa D.: 2670, 3560
Breton, Juliana: 6570
Brewster, Glenna S.: 3482, 6600, 9030
Brewster, Katharine K.: 3600
Brickman, Adam: 4470
Bridges, Katherine: 3700
Bridges, Preston B.: 6020
Brinson, Jessica: 3560
Brintz, Benjamin J.: 1330
Brisendine, Matt: 5091
Bristol, Alycia: 3500
Brito, Tábatta: 9020
Brittany, Dennis: 4190
Britt, Katherine C.: 6260, 9020
Brodrick, Marisa: 6310
Brody, Abraham: 2130, 3060, 6110
Brody, Lilla: 2000, 6310, 6530
Brody, Rebecca: 4680
Broen, Tiana: 6390
Brom, Heather: 1530
Bronas, Ulf G.: 4800, 6411, 6780
Bronskill, Susan: 9000
Brooks Carthon, Margo: 1530
Brooks, Amber K.: 2300, 6180
Brosius, Tracy: 3460
Brothers, Allyson: 5110
Brotherson, Sean: 6780
Browdie, Richard: 3180
Brown-Borg, Holly: 2180
Brown, Casey L.: 4850, 9000
Brown, Cynthia J.: 3350
Browne, Julia: 6170
Brown, Ellen L.: 9000
Browne, Tarah B.: 9020
Brownfield, Douglas: 1470
Brown-Hughes, Travonia: 2070
Browning, Wesley R.: 6750, 9000
Brown, J. Scott: 6520
Brown, Jocelyn: 9000
Brown, Lana M.: 6220, 6360
Brown, Lauren: 6200
Brown, Lisa M.: 4465, 6010
Brown, Maria T.: 3000, 6300
Brown, Monique J.: 3760
Brown, Patrick J.: 3600
Brown, Rebecca: 6140
Brown, Ryan: 9010
Brown, Shelby: 6540
Brown, Susan L.: 1300, 4070
Brown, Susan H.: 9000
Brown, Teressa: 1420, 6740
Brown, Tyson: 6180
Bruce, Marino A.: 6180
Bruefach, Tyler: 2200
Bruellman, Ryan: 9010

Brunton, Stephen: 9000
Brush, David M.: 4480
Bruzios, Kathryn: 4900
Bryan, Stirling: 4720
Bryant, Chalandra: 9030
Bryant, Natasha S.: 2720, 6210, 6760
Bryanton, Olive: 6250
Bryk, Kelsey: 6450, 9000
Bubu, Omonigho M.: 6680
Buchanan, Scott: 9020
Buchan, Jacob: 9000
Buchmann, Nikolaus: 1010
Buchwalter, Abigail: 5091
Buck, Harleah: 1180, 3910, 3930, 4650, 9020
Buckley, Thomas D.: 2700, 6610, 6680
Buckwalter, Kathleen C.: 3680
Bucy, Taylor: 3130, 3482, 3660, 3910, 5130, 6540
Budhathoki, Chakra: 4640
Budhwani, Hina: 6740
Bueno, Michael V.: 2250
Bui, Cindy N.: 2440
Bui, Nhat Minh: 6270
Bulanda, Jennifer R.: 6260, 6520
Bulgarelli, Alexandre F.: 6100
Bull, Amanda: 6230
Bullock, Avery L.: 6800
Bullock, Karen: 9030
Bülow, Per: 9010
Bunch, Jacinda: 9020
Bunker, Jennifer: 3830
Burack, Orah R.: 6100
Burant, Christopher J.: 3115, 5140, 6090, 6160, 6820, 9030
Burch, Hannah: 6770
Burgdorf, Julia G.: 1180, 4640
Burek, Sam: 5001
Burgess, Elisabeth: 5173, 6760
Burgos Zuniga, Claudia: 6000, 6220
Burke, David T.: 9000
Burke, Emily S.: 6100, 6280
Burke, Robert E.: 3130
Burk-Leaver, Erin Y.: 3760
Burman, Bonnie K.: 3530
Burnes, David: 4090, 6480
Burnette, Denise: 2700, 6610
Burnett, Jason: 4890
Burnham, Dyanna: 6430
Burns, Annette: 2690
Burns, Catherine: 2460
Burns, Edith: 4700, 9000, 9010
Buron, Bill: 9000
Burr, Jeffrey A.: 2040, 3220, 6670, 6810
Burrow, Anthony L.: 2430
Burston, Betty: 1070, 6090
Burton, Cassandra: 3700
Burton, Ellen: 1190
Busby-Whitehead, Jan: 9030
Buse, Caroline: 6420
Buta, Brian: 1010
Butler, Brenda: 3860
Butler, Jason: 6840
Butler, Jorie: 4010
Buxton, Orfeu M.: 2620, 4080, 6600, 9010
Buys, David R.: 6340, 9030
Buysse, Daniel J.: 4080
Bybee, Sara: 1320, 2540
Byers, Amy L.: 2510
Byers, Chelsey: 5082, 5130, 6000
Bylund, Peggy R.: 6590
Bynum, Julie: 3130, 6060, 9010
Byrd, DeAnnah R.: 6290

C

Cabrero Castro, Jose Eduardo: 2580
Cacchione, Pamela: 9000
Cadar, Andreia N.: 9010
Cadmus, Eniola O.: 3900, 3920
Caffrey, Christine: 6100, 6540
Caffrey, Melanie: 9000
Cagle, John: 1390, 4510, 6730
Cagney, Kathleen A.: 6550
Cagnin, Annachiara: 1030
Cain, Stephen M.: 9000
Cai, Yurun: 1280, 4040, 4120, 6740, 9030
Calamia, Matthew R.: 6830
Calasanti, Toni: 6210, 6750
Caldera, Selena: 2650
Calfee, Kristin: 4080
Calkins, Margaret: 6760, 9030
Callahan, Christopher: 4280, 6220
Callan, Judith: 9010
Calo, William A.: 3520
Calzada, Christopher: 9020
Camacho, David: 2700, 4225, 5150, 6180
Camacho, Simone: 6070, 6720
Cambia, Jansen M.: 3483
Cameron, Jessica: 6790
Cammer, Allison: 9000
Campbell, Jessica: 9000
Campbell, Kaitlyn M.: 2520
Campbell, Laura M.: 2450
Campbell, Margaret: 4160
Campbell, Noll: 6510, 6590, 9000
Campetti, Randi: 4890
Campisi, Judith: 3241, 9010
Campitelli, Anthony: 6450, 6510, 9000
Camp, Janella: 6200, 9000
Camp, Kathlene: 1250
Canell, Anastasia E.: 6130, 6310
Cannon, Rachel: 6530
Can, Reyyan: 3490
Cantu, Phillip A.: 3020, 6210, 6300, 9020
Cao, Jiepin: 9020
Cao, Katy (Qiuchang): 4370, 5080
Cao, Kevin: 3910
Cao, Yuxin: 3450
Capezuti, Elizabeth: 9010
Capolino, Alexandria C.: 3780, 6400
Cappon, Davide: 3482
Caprio, Thomas V.: 1110, 4580, 6140, 9010
Carbonell, Ellen: 2560
Cardenas, Valeria L.: 6120
Carder, Paula: 3200, 3910, 4022, 6540
Carey, Rebecca: 3460
Carino, James: 2460
Carling, Stacy: 3460
Carlson, Bryant: 4022
Carlson, Michelle C.: 3690, 3750
Carman, Katherine: 6440
Carmichael, Alicia G.: 2420
Carnahan, Jennifer L.: 4100
Carneiro, Francis: 6180
Carpenter, Brian D.: 2030, 3420, 4480, 6160, 6520, 6730, 9010, 9020
Carpenter, Joan G.: 3520, 9020
Carpenter, Matthew: 9030
Carr, Alan: 4225, 6580
Carrasquillo-Sánchez, Kiara: 2700
Carr, Dawn: 1230, 2200, 9020
Carr, Deborah: 1450, 1490, 3070
Carre, Nicole: 9020
Carrico, Catherine: 3460
Carrillo, Alejandro: 2220, 6640
Carrion, Iraida V.: 3550
Carr, Megan: 9000
Carroll, Brooke: 9020
Carroll, Judith E.: 1100, 3400, 3440, 4560
Carroll, Simon: 2600
Carstensen, Laura: 3190, 6170, 9010
Carter, Kara: 1090
Carter, Lorraine: 6000
Carttar, Tennery: 9010
Carver, Chase M.: 3241, 6010, 6411
Cary, Michael P.: 9010
Casanova, Giuliana: 6620
Casanova, Ramon: 9000
Case, Donna J.: 4020, 4023
Case, Sami L.: 6411
Casey, Martin F.: 9030
Cash, Antonia: 6680
Caskie, Grace I. L.: 6130, 6310
Caso, Tabatha: 9030
Cassady, Mara: 9030
Cassatt, Sarah: 5050
Cassidy, Jessica: 6530, 9000
Castagna, Nicolette: 6420
Castaneda-Avila, Maira A.: 2640
Castaneda, Salvador: 2560
Castle, Steven: 4260
Castro-Portuguez, Raul: 6840
Castro, Saul: 4060
Cattaneo, Gabriele: 3510
Cauley, Jane A.: 2290, 4080, 4120, 6650
Cauley, Jane A: 9030
Cavalcante, Ricardo: 6100
Cavalli, Stefano: 6380
Cawthon, Peggy M.: 2290, 3340, 3690, 4080, 4120, 4600, 6200, 6320, 6780, 9010, 9020, 9030
Cazet, Jack F.: 3810
Celardo, Caitlin: 3130
Cene, Crystal: 2200
Cenko, Erta: 6510
Centi, Sophia: 6280
Cenzer, Irena: 5030
Cerbone, Felicia G.: 4240
Cerino, Eric S.: 2430, 2470, 9020
Cerino, Lauren: 6310
Ceruso, Marco: 9030
Cervantes Navarrete, Matilde: 2600
Cervantes, Diana E.: 1110
Cetkowski, Richard: 6560

Chai, Hye Won: 6190, 9030
Chakraborty, Papiya: 1470
Chakravarty, Esha: 6430
Chakravarty, Indrani: 6430
Chalasani, Suresh B.: 9030
Chamberlain, Stephanie: 4210, 5010
Chambers, Kevin: 3100
Chamut, Steffany: 6230
Chan, Alex Siu Wing: 9020
Chan, Angelique: 4170
Chan, Athena Chung Yin: 6310
Chan, Cecilia Lai Wan: 4022, 4684, 5084
Chandler, Michael: 9000
Chandra, Rashmi: 6411
Chane, Samson: 6330
Chaney, Kimberly E.: 5000
Chang Gudjonsson, Milan: 1190, 6220, 6800
Chang, Chiang-Hua: 9010
Chang, Da Jung: 6220
Chang, E-Shien: 4900, 9000, 9030
Chang, Haeyoon: 9010
Chang, Juliet: 3750
Chang, Jun Ha: 6420
Chang, Ling-Hui: 4450, 6350
Chang, Megan: 6740
Chang, Pei-Fen: 6760
Chan, Grace Man Yee: 4022, 4684, 5084
Chang, Sun Ju: 6320, 6510, 6650
Chang, Yu-Chien: 6540
Chang, Yu-Ping: 6110, 6280, 6510, 6770
Chang, Yu-Ting: 9010
Chan, Keith T.: 3860, 9010, 9030
Chan, Kiera: 9010
Chan, On Fung: 9030
Chanti-Ketterl, Marianne: 4420
Chao, Linda: 3050
Chao, Shiau-Fang: 6240, 6700, 6800
Chao, Tony C.: 1120
Chao, Ying-Yu: 4700, 6480
Chapadia, Bunsi: 9010
Chaperon, Claudia: 6280
Chapman, Brian: 9020
Chapman, Joyce: 4681
Chapman, Karen R.: 1120
Chapman, Silvia: 4470, 6260
Chappell, Amanda N.: 6210
Chappell, Richard J.: 2670
Charara, Rima: 6220
Chard, Sarah E.: 9010
Charles, Doreek: 1310
Charles, Susan T.: 2430, 2470, 3730
Charness, Neil: 3410, 4400, 6290, 9020
Chase, Jo-Ana: 2250
Cha, Seung-Eun: 6750
Chasteen, Alison: 5000, 6640
Chataut, Akankshya: 6390
Chaudhari, Diptaraj S.: 9000
Chaudhari, Nikhil: 9000
Chaudhari, Piyush V.: 9020
Chaudhury, Habib: 3450, 4090, 6500
Chaves, Paulo H M.: 4490
Cheek, Dana: 4830
Chee, Kyong Hee: 4660
Chee, Wonshik: 9030
Chei, Choy Lye: 6290
Chen, Cynthia: 3390, 3620, 3820, 4220, 6330, 6770, 9020, 9030
Chen, Danica: 4001
Chen, Diefei: 4870
Chen-Edinboro, Lenis P.: 1030, 6350
Chen, Frances L.: 9010
Cheng, Grand: 4170
Cheng, Kent Jason G.: 6520
Cheng, Mengling: 6200
Chen, Gong: 9010
Chen, Guangzhou: 3270
Cheng, Yan: 6790, 5173
Cheng, Zhaowen: 6170
Chen, Honglei: 2670
Chen, Hongtu: 4700
Chen, Huashuai: 1100
Chen, Jen-Hao: 3040
Chen, Jiachen: 6450, 6740
Chen, Jiu-Chiuan: 3510
Chen, John S.: 9020
Chen, Kuan-Hua: 4681, 4850, 9000
Chen, Lei: 3770
Chen, Li: 6200
Chen, Lu: 3020
Chen, Mei-Lan: 6780
Chen, Michelle: 9010
Chen, Ping: 1280
Chen, Randi: 3580, 6010
Chen, Ruijia: 4690
Chen, Samson: 4681
Chen, Siting: 6450
Chen, Tuo-Yu: 6330, 6600, 9030
Chen, Xi: 1000, 5010, 6450
Chen, Xia Yu Summer: 9020
Chen, Xiaomeng: 4600
Chen, Xiayu S.: 4370, 6650
Chen, Ya-Mei: 6090, 6350, 6540
Chen, Yanjun: 2670
Chen, Yun-Zih: 6760
Chen, Yuxuan: 4681, 4850, 9000, 9010
Cherry, Katie E.: 6830
Chesney, Margaret A.: 3050
Cheung, Ethan Siu Leung: 4240, 6230, 6500
Cheung, Karen Siu Lan: 4022, 4684, 5084
Cheung, Krystal: 6430
Chew, Hui Jun: 2210
Chia, Ngee Choon: 3820
Chidebe, Runcie: 6430
Chien, Jessie: 6290
Chien, Shao-Yun: 6420
Chihuri, Stanford: 5171
Chi, Iris: 1500, 1540, 3870, 6240, 6680, 6750, 9030
Chikama, Hitomi: 6450
Chi, Kevin: 9010
Childs, Elisa: 6720
Chima, Emmanuel: 4770
Chin, Janecca: 1450
Chin, Jessie: 9020, 9030
Chintapanyakun, Teepatad: 3840
Chippendale, Tracy: 3880, 6110
Chiriboga, David: 6490, 6810
Chiriboga, Germán: 6140
Chisholm, Lilly: 4020
Chittams, Jesse: 1530
Chiu, Chi-Tsun: 4420
Chiu, Venus: 4330
Chiu, Wan-Yu: 6350
Chmelka, Gina: 3130
Choate, Ashley: 5083
Cho, Dongwook: 9010
Chodosh, Joshua: 1560
Cho, Eunhee: 6080, 6270, 6300, 6320, 9000
Cho, HanNah: 6390, 6730
Cho, Hyunkeun: 3240
Choi-Allum, Lona: 6480
Choi, Bomi: 6390
Choi, Catherine: 6010
Choi, Chris: 9000
Choi, Eunyoung: 3220, 4690, 5000
Choi, Ga-Young: 6480
Choi, Gi Won: 6320, 6510
Choi, HwaJung: 3130
Choi, Hyo: 6010
Choi, Hyojin: 4510
Choi, Jean: 9000
Choi, JiYeon: 6400
Choi, Mi Sun: 6500
Choi, Namkee G.: 2240, 6350
Choi, Roy B.: 6840
Choi, Seongmi: 6400
Choi, Seonhwa: 6270
Choi, Won: 2550
Choi, Yeon Jin: 6180, 6240, 6310
Choi, Yong K: 9020
Choi, Yoonseok: 6210, 6390, 6580
Cho, Jinmyoung: 6100, 9010
Cho, Joonyoung: 6090
Chong, Natalie: 5070
Chong, Wayne F.: 2290, 3840
Choo, Esther: 5100
Chopik, William J.: 1240, 2220, 2280, 4010, 6640
Chopra, Anita: 6560
Cho, Seungjong: 6820
Cho, Sunghwan: 2700
Cho, Susie: 1150
Cho, Tsai-Chin: 2270, 3130
Chou, Andrew: 9020
Choula, Rita B.: 2650
Choun, Soyoung: 5140, 6210, 9020
Chow, Angela: 9020
Chow, Esther O.: 6350, 6530
Chow, Natalie: 6390
Chow, Stephanie W.: 2730, 4250, 6320
Cho, Youngmin: 5010, 6450
Cho, Youngshin: 6420
Christensen, Kaare: 4810, 6010, 9030
Christian, Thomas: 1310, 6720
Christman, Eva-Marie A.: 4260
Christy, Jacob: 1540
Chua Qian Ling, Denise: 4023
Chubinski, Jennifer: 3160
Chu, Charlene H.: 1420, 6640, 9000
Chuengsatiansup, Komatra: 4700
Chui, Cheryl Hiu-Kwan: 9030
Chui, Helena: 9000
Chu, Li: 3190, 6170
Chun, Alice: 3390
Chunga, Richard E.: 2720
Chung, Edwin: 6390
Chung, Edwin K. H.: 3630
Chung, Gawon: 6060

Chung, Jane: 3670, 6070
Chung, Joe: 9030
Chung, Misook: 6280
Chung, Shang-En: 5110
Chung, Soondool: 6490
Chung, Tammy: 3860
Chung, Wonock: 6710
Chun, Sanghee: 6380
Chun, Yung: 9000
Churchill, Linda C.: 2290
Chyr, Linda C.: 6760
Chyu, Joanna: 6210
Ciaralli, Spencier R.: 6430
Ciccone, Isabella S.: 6110
Cicero, Ethan: 2390, 3290
Cichy, Kelly E.: 2440
Cidav, Tom: 4500
Cigolle, Christine: 6420
Cimarolli, Verena R.: 2720, 6210, 6760
Cinader-Galvan, Morgan: 6420
Ciolfi, Mary Lou: 6540
Cipher, Daisha M.: 1080, 6060, 6630
Claiborne, Alex: 9010
Clair, Catherine A.: 3330
Clark, Callahan: 6110
Clark, Daniel: 6270
Clarke, Anthony J.: 2290
Clarke, Barry: 1180
Clarke, Peter J.: 9000
Clarke, Philip B.: 4060, 6250
Clarke, Philippa: 1050, 2570, 3690
Clark, Jaylyn F.: 6180
Clark, Jeff: 3460
Clark, Josef P.: 1140
Clark, Katherine: 2610
Clark, Phillip G.: 6770
Clarkson, Benjamin: 3241
Clark, Sunday: 4900
Clay, Olivio J.: 6750
Cleary, Jennifer: 1290
Clem, Sarah E.: 6340
Clevenger, Carolyn: 5160
Click, Mary: 9010
Cline, J. Mark: 9010
Cline, Sarah M.: 9000
Cloud, Leslie J.: 6420
Clouston, Sean: 3440, 3510, 4700, 5173, 6200, 6440, 6680
Cloyes, Kristin G.: 1320, 2070, 2310, 2540
Coates, Emma: 6110
Coates, Martha C.: 5020, 9020
Cobb, Sandy: 9020
Cobb, Sharon: 9020
Coccia, Kathryn: 6720
Cochrane, Barbara: 1150
Cochran, Lelah: 4160
Coen, Paul M.: 3340, 4600, 9010, 9020, 9030
Coffman, Cynthia J.: 5083
Cohen Quay, Lisa: 6500
Cohen, Alan: 6140
Cohen, Dan: 4270, 9000
Cohen, Debbie: 9030
Cohen, Harvey J.: 3400
Cohen, Iris: 6070
Cohen, Ken: 6110
Cohen, Marc: 1260, 3320, 4270, 6140
Cohen, Wendy: 6060
Cohen, Yafit: 1300
Cohn-Schwartz, Ella: 2480
Colón, Lisa: 5150
Coldiron, Allyson M.: 6050, 6410
Coleman, Carissa K.: 2520, 2530, 4030, 5010
Coleman-Hulbert, Anna: 9010
Coleman, Max E.: 6810, 6820
Coleman, Rachel: 3540, 6270, 9030
Cole, Rachel C.: 9020
Cole, Robert N.: 6411
Cole, Steve: 3440, 4560
Coletta, Giulia: 3620
Colichino, Elena: 1350
Collado, Elias: 3483, 4021, 6740, 9020, 9030
Collie, Angel: 9000
Collins-Pisano, Caroline: 3481
Collyer, Taya: 5083
Compernolle, Ellen: 1230, 5150, 6440
Cong, Zhen: 1540
Coniff, Joshua: 4220
Connell, Cathleen: 2420, 3130
Connelly, Caitlin: 9020
Conner, Norma: 6700
Connolly, Marie-Therese: 4090
Conrad, Karen: 4900
Conrad, Ken: 4900
Conroy, Molly B.: 1120
Conti, Jennifer L.: 1040
Convery, Kimberly: 2130, 6110
Conway, Adam: 1320
Conway, Cathy: 6680
Conwell, Yeates: 4580, 6270
Conyers, Christian: 1170
Coogle, Constance L.: 2260
Cook, Diane J.: 6420
Cook, Summer B.: 6780
Coon, David W.: 2250
Cooper, Hannah LF: 9020
Cooper, Jasmine: 1240
Cooper, Lisa A.: 4680
Cooper, Lujira J.: 2540
Copeland, Molly: 1230
Cope, Marie T.: 6520
Corazzini, Kirsten N.: 1060, 3170, 6110, 6280, 9010
Corbit, Nancy: 5171
Corcoran, Mary: 9020
Cordell, Ashlee: 6150, 6370, 6390
Coresh, Josef: 2670
Corley, Connie S.: 6350
Corley, Robin: 9000
Cornell, David J.: 2610
Cornell, Portia Y.: 3130, 5081
Cornish, Stephen: 6780, 9010
Coromac-Medrano, Juliamaria: 6730
Cortes, Constanza J.: 5091
Cortes, Tiffany M.: 6140, 6320
Cosarderelioglu, Caglar: 6411
Cosentino, Stephanie: 4470, 6260
Cosenza, Carol: 9020
Costantini, Hiroko: 6310
Costello, Emily: 6120
Cotten, Shelia R.: 4020, 9030
Cottler, Linda B.: 9020
Cotton, Quinton D.: 3750
Cotton, Sam: 1110
Coughlin, Joseph: 3260, 3450, 4090, 6310
Coulourides Kogan, Alexis M.: 3390
Coulson, Alison: 2160
Coursaris, Constantinos: 4770
Covinsky, Ken E.: 5030
Covinsky, Kenneth e.: 5030, 6110, 6320, 6780
Cox Sullivan, Sheila: 6360
Cox, Brian: 6100
Cox, Keri: 6570
Cox, Nicholas: 2060
Coyle, Caitlin: 1170, 2010, 6090, 6700
Coyle, Caitlyn: 2050
Cozad, Aisha Bonner: 3700
Cozad, Hannah O.: 9000
Crabtree-Ide, Christina R.: 6430
Craft Morgan, Jennifer: 5173
Crandall, Carolyn J.: 4080
Crandall, Jason: 6570
Crane, Breanna M.: 3690, 3750
Crane, Michael: 1350
Cranley, Lisa: 1420
Cranston, Teresa A.: 6100
Crary, John F.: 6010
Crawford, Andrew: 3483, 4021, 6740, 9020, 9030
Crawford, Andrew Carl: 9030
Cremer, Svenja: 6270
Crespo-Fierro, Michele: 2130
Crews, Deidra C.: 2510, 4680
Criado Carrero, Ricardo: 4021, 9020, 9030
Crimmins, Eileen: 2080, 3260, 3440, 4560, 5000, 6010, 6710, 6830
Crittenden, Jennifer: 3540, 6270, 6540, 9030
Crivello, David A.: 4260
Croft, Janet: 3510, 4120, 6280
Crosby, Paris: 5140
Cross, Dori A.: 3130, 3482, 3660
Crosswell, Alexandra D.: 6830
Crouch, Adele: 3680
Crouch, Maria C.: 1380, 4710
Crouter, Scott: 9020
Crowder, Kim D.: 1420
Crowe, Michael: 4225, 6200, 6590, 6750
Crowley, Christopher: 6770
Crowley, Max: 4060
Cruickshanks, Karen J.: 2670
Cruz, Grace: 6600, 9030
Cruz-Oliver, Dulce: 6140
Cudjoe, Thomas KM: 2530, 2690, 3130, 6800
Cuellar, Alison E.: 3130
Cui, Can (Shirley): 9000
Cui, Chang-Yi: 1470
Cui, Ruifeng: 9010, 9030
Culberson, John: 6760
Cullen, Jennifer: 6510
Cullen, John: 2090
Culminskaya, Irina: 6010
Cummings, Steve: 3340, 6780, 9010, 9020
Cummings, Steven R.: 4600

Cummins, Phyllis: 4430, 4555, 6130, 6670, 6710
Cundiff, Jeanne M.: 9020
Cunich, Michelle: 6280
Cunningham, Chris: 2410, 3770
Cureton, Bianca R.: 9020
Curl, Angela L.: 5150, 6260, 6470
Currie, Katherine L.: 4150
Curtis, Brenda: 6170
Curtis, Danny: 6740
Curtis, Laura: 1030
Cutchin, Malcolm: 6650
Cutler, Ronald: 1200
Cvejkus, Ryan K.: 3400, 4600, 4810, 6450
Czaja, Sara: 2000, 3410, 4090, 6480, 6530, 9010, 9030
Czajka, Agnieszka: 4684
Düzel, Sandra: 1010

**D**

Dabelko-Schoeny, Holly: 2010, 3770, 4370
Dabiri, Sanaz: 6170
Dabrowski, Barbara: 3460
Dahal, Poshan: 6690
Dahal, Roshani: 9010
Daks, Jennifer: 2660
Dalal, Yamini: 1200
Dale, William: 2550
Dallas, Mark: 5001
Dallas, Sarah: 5001
D'Ambrosio, Lisa: 3260, 3450, 4090, 6310
D'Amico, Danielle: 4490, 6440
Damier, Sashah: 6220
Dammer, Eric: 6840
Damush, Teresa M.: 6390
Dandrea, Kaye R.: 6100
Danesh, Valerie: 6100
Dangerfield, Hannah J.: 6770
Dang, Hope: 6840
Dang, Linh: 1160, 4630
Dang, Stuti: 1330, 4010, 6420
Dang, Weiwei: 1470
Daniel, George: 6170
Daniel, Kathryn M.: 1080, 6630, 6770
Danielson, Ramona A.: 6280
Danilovich, Margaret: 4021, 4023, 4270
Danish, Madina: 9000
D'Antonio, Patricia M.: 2100, 3120, 4400, 4691
Daquin, Jane C.: 9000
Darby, Nina: 6530
Darling, Nancy: 6520
Darvishpoor Kakhki, Ali: 3620
Dash, Kim: 4890
Das, Sai Krupa: 2180
Dash, Sanjana: 9020
Das, Ranjit Kumar: 9020
Dassel, Kara B.: 3030, 3710, 9010
Dauenhauer, Jason: 2350, 9030
Daugherty, Ana M.: 6290
Daum, Christine H.: 6390, 6560, 6680
Davagnino, Judith: 6420
Davatzikos, Christos: 4200
Davenport, Chad: 1120
Davenport, Kathleen: 9030
Davey, Adam: 9030
Davidson, Patricia: 6140
Davidson, Patrick: 6290
Davies, Nathan: 3555
Davila, Heather: 4770
Davis, Amanda: 1180
Davis, Andra: 9000
Davis, Elizabeth: 2560
Davis, Jamaine: 1440
Davis, Kimberly D.: 2260
Davis, Linda: 4660
Davis, Rebecca L.: 6760
Davis, Shelly: 6180, 6280
Davis, Taylor: 4461
Davitt, Joan K.: 9000
Davlyatov, Ganisher: 3660, 4820
Davy, Philip MC: 3580
Dawkins, Emily: 6270
Dawson, Walter: 4790, 9000
Dayama, Neeraj: 6420
Dayanidhi, Sudarshan: 2500
Dayrit, Jessika G.: 6210, 9030
Day, Ryan: 9030
de Boer, Bram: 1160, 2090, 3555, 4300
de Bruin, Simone: 4550, 9020
De Diego Diaz, Ana: 9020, 9030
De Fries, Carson M.: 4240, 4430, 6500
De Geest, Sabina: 6270
de Klerk-Rubin, Vicki: 6000
de Leeuw, Sarah: 4020
De Lima, Bryanna: 9030
De Main, Atami: 6260
de Man-van Ginkel, Janneke: 6270
de Medeiros, Kate B.: 3585, 4180, 4670
de Paulo Couto, Clara: 2480
de Rosa, Cristina: 6280
de Vries, Brian: 6750
De Vries, Erica: 1160
Deal, Jennifer A.: 2270, 2530, 2670, 3560, 4050, 4330
DeAnda, Jaden: 6490
Dean, Elizabeth: 6010
DeAngelis, Reed: 6180
Dearborn, Lynne M.: 4170
Deardorff, W. James: 5030
Deason, Karley: 6000
Deason, Rebecca G.: 6160
DeCarli, Charles S.: 3600
DeCaro, Jason A.: 9020
DeCherrie, Linda: 6610
Decker, Autumn E.: 4900
Decker, Elyse: 4710
Deemer, David A.: 5172
Degenholtz, Howard B.: 1400, 2590, 3660, 9030
DeGennaro, Jennifer: 6560
DeGroot, Lyndsay: 3330
Deichert, Jerome: 6700
Deimling, Gary T.: 2640, 6430
del Pino, Homero E.: 3750
Delaney, Colin: 1200
Delano-Wood, Lisa: 9000
DeLauder, Rachel M.: 4021
Delbaere, Kim: 4340
Delgado, JoJo: 6100
Delgado-Rodriguez, Annaliet: 4750
Delhaes, Flavien: 6380
DeLiema, Marguerite: 3160, 4900
DeLiema, Marti: 6360
Deller, Juergen: 6700
Delvecchio, Julia: 3620
DeMarshall, Cassandra: 6411
Dembinski, Kimberly: 6780
Demeo, Gianna: 6770
Demeter, Naor: 1300
Demiris, George: 3670, 6390, 6730
Demuth, Ilja: 1010, 3070, 4040, 6010, 6641
Denhaerynck, Kris: 6270
Denning, Emily C.: 6340
Dennison, Christopher: 4070
Denny, Bryan: 3481
Denson, Kathryn: 6280
Depp, Colin A.: 2450
Derain, Lily: 6750
Desai, Pankaja: 9010
Desai, Shreya: 1330, 6430, 6530
Desai, Smit: 9020, 9030
DeSanto-Madeya, Susan: 6140
Deshmukh, Maya: 6110
Deshpande, Falguni: 9020, 9030
Desir, Marianne E.: 1330
Dessale, Shambel: 6330
Deters, Kacie D.: 1440
Devanand, Davangere P.: 2160
Devane, Karan S.: 9000
DeVengencie, Faith: 2340
Devereaux Melillo, Karen: 2610
Dey, Judith: 3640, 4350
Dey, Judy: 3640
Dezine, Marie L.: 6220
Dhakal, Usha: 9010
Di Gessa, Giorgio: 3210, 6310
Diane Ploch, Emily: 6310
Diaz, Arnold: 9020
Diaz, Fernando Erra: 3001
Diaz, Jose A.: 3770
Diaz-Ramirez, L. Grisell: 3690
Díaz-Santos, Mirella: 3750
Diaz-Valdes, Antonia E.: 6350
Diaz, Vivian: 9010
DiBello, Julia R.: 3230
Dichter, Melissa E.: 9000
DiCicco, Heather: 1340
Dickerson, Anne E.: 6470
Dickman Portz, Jennifer: 2280, 3360, 3930, 4650
Dickson, Chelsea: 9030
Dicks-Williams, Ramona: 6750
Dictus, Cassandra M.: 4360, 5010
Dieciuc, Michael: 1570
Diefenbach, Michael: 4700
Diehl, Christin: 1250
Diehl, Cody L.: 9000
Diehl, Manfred K.: 5110
Diekemper, Karla A.: 6220
Dieker, JoAnna L.: 6750
Dierckx de Casterlé, Bernadette: 6560
Dietrich, Mary: 1180
Digbeu, Biai: 9010
DiGirolamo, Grace: 5091
DiGuiseppi, Carolyn: 5171, 9030
Dikhtyar, Oksana: 3820, 4430
Dilanchian, Andrew T.: 1570, 3410
Dillon, Ellis: 1310

DiMaria-Ghalili, Rose Ann: 1120, 2610, 4870, 5020, 9020
Dinaj, Veronica: 6790
Ding, Hui: 6530
Ding, Jingzhong: 2241, 3400
Ding, Kedong: 9020
Ding, Tianli: 9010
DiNitto, Diana M.: 6350
Dinman, Michele: 3800
Dioh, Waly: 9010
Dionne-Odom, J. Nicholas: 6390
DiScala, Sandra: 6000
Disini, Bridget: 6500
Distefano, Giovanna: 3340, 9010
Dixon, Bradley J.: 6450
DJousse, Luc: 3600
Đoàn, Lan N.: 1480
Dobalian, Aram: 2660
Dobbins, Jessica: 3540
Dobbs, Debra: 2720, 3910, 4130, 4465, 4650, 4820, 6070
Dodge, Hiroko H.: 2450, 3580, 4870
Do, Elena: 9020
Dohan, Daniel: 9000
Doherty, Caroline: 3100
Dokos, Malinda: 6560
Dolan, Hanne R.: 9030
Dolansky, Mary: 1530
Dolbin-MacNab, Megan L.: 4060
Domingue, Ben: 3000
Domoto-Reilly, Kimiko: 6420
Donath, Carolin: 4300
Donehower, Allison K.: 1300
Dominguez, Mauricio: 5001
Dong, Fanghong: 6510
Dong, Juan: 6641
Dong, Linh: 6290
Dong, XinQi: 3870, 4700, 6480
Dong, Yu: 6780
Donlon, Timothy: 3580, 6010
Donnellan, Kelsey M.: 3010, 6060
Donnelly, Rachel: 4460
Donofrio, Abigail: 6770
D'Onofrio, Gail: 5100
Donohoe, Maria: 3240
Donohue, Erin: 6760
Donorfio, Laura KM: 3030, 9020
Dooley, Erin E.: 4050
Doraiswamy, P. Murali: 2241
Doran, Kevin: 1260
Dorr, David: 6340, 6740
Dorris, Jennie L.: 1340
Dorsey, Susan G.: 6641
Dos Santos, Camila Mello: 6100
Dosa, David M.: 3200, 4465, 4820
Douglas, Jason A.: 3440
Douglas, Natalie F.: 1270, 2340, 3780
Douglas, Sara: 6510
Dowd, Jennifer B.: 6050
Downer, Brian G.: 2580, 4225, 6300
Downer, Paige B.: 9020
Downey, Laura: 6280
Doyle, Cassidy: 1100, 3540, 6070
Doyle, Margaret F.: 6450, 6740
Doyle, Margaret: 5172
Doza, Adit: 4040
Dozier, Mary E.: 4021, 9000
Drabo, Emmanuel: 6760
Dragan, Deanna: 5082
Drake, Joshua: 5091
Drance, Elisabeth: 1420
Drazich, Brittany F.: 3750, 4760
Dreer, Laura: 6750
Drewelies, Johanna: 1010, 3070, 3150, 6641
Dreyer, Jan: 3090
Driesse, Tiffany M.: 4640
Driessnack, Martha: 3240
Driggers, Amelia: 6340
Driscoll, Monica: 3810, 9010
Driver, Jane A.: 3600
Dronamraju, Neha: 6270
Droste, Andrew P.: 9000
Drost, Jennifer: 1190
Drucker, Claire: 6770
Drummond, Neil: 6150
Dryden, Eileen M.: 1040
Duangjina, Thitinan: 3500
Duan, Haoshu: 6750
Duan, Hongzhe: 1390, 4290, 9000
Duan, Yinfei: 4210
Duara, Ranjan: 6140
Duarte, Andres: 6140
Dubal, Dena: 3001
Dube, Catherine E.: 9020
Dube, Jeanné: 6050, 6410
Duberstein, Paul R.: 4500
Dube, Victoria P.: 6090
Dubno, Judy R.: 9030
Duchesneau, Emilie: 4320
Duchowny, Kate: 3690
Duezel, Sandra: 1010, 3070, 4040, 6641
Duffy, Karen: 9020
Dufour, Alyssa B.: 3600
Dugan, Beth: 1090, 3100, 6090, 6200, 6510
Duggan, Katherine: 6580
Duggan, Mariu: 6270
Duggleby, Wendy: 1420
Duke Boynton, Felicia D.: 6010
Dulaney, Cynthia L.: 6790
Dulaney, Sarah: 6270
Dunbar, Annie Zean R.: 4430
Dunham, Katherine: 9020
Dunkle, Ruth E.: 6090, 6230
Dunlap, Janette: 6420
Dunlap, Pamela: 3690
Dunning, Lauren: 3540
Dunn, Nicole J.: 6780, 9010, 9030
Duodu, Vennesa: 6140
Dupre, Matthew E.: 1540, 5140
Dupuy, Emma G.: 6570
Durduran, Turgut: 6450
Durga, Aditi: 2130, 3060, 6110
Du, Shufa: 2080
Duthie, Edmund: 6280
Du, Yan: 4190, 4682
Dvorak, Katy: 3550
Dvorak, Robert: 4480
Dye, Cheryl: 4500
Dykstra, Taitea: 4461
Dys, Sarah: 2570, 3200, 4022
Dzierzewski, Joseph M.: 2030
Dzikowski, Natalie: 4620
Dziura, James: 5100

### E

E Kheirbek, Raya: 6350
Easter, Bashir: 6750
Eastman, Marisa R.: 4110
Easton, Scott: 4730
Eaton, Jacqueline: 4520, 9000
Ebeling, Peter: 5083
Ebili, Ujunwa: 2510, 6240
Eby, David W.: 3000, 6470
Echt, Katharina: 9010
Eckstrom, Elizabeth: 9030
Edelman, Linda S.: 2060, 2310
Edelstein, Barry: 9030
Edland, Steven: 6411
Edney, Sarah: 3620
Edwards, Chelsea: 5100
Edwards, Jerri D.: 9000
Egbuchiem, Henry: 2510, 6240
Egeberg, Eva: 9000
Egert, Michal: 1300
Ehlman, Katie: 6220
Ehrlich, Joshua: 3690, 4690, 6680
Eich, Teal: 6290
Eid, Fatima: 5060
Eifler, Morgan: 1030
Einstadter, Douglas: 4100
Eisenson, Howard: 9000
Eisenstein, Amy: 1080, 4590
Ejaz, Farida K.: 3920, 4770, 4900, 5083, 6150, 6370, 6390
Ekerdt, David J.: 6710
Ekram, A R M Saifuddin: 4320, 6320
El Shanawany, Perihan: 9000
El Sharu, Husam: 6590
Elam, Kit: 9020
Elayoubi, Joanne: 6290
Elbogen, Eric B.: 6170
Eldadah, Basil: 2160, 3080
Elder, Keith: 9010
Eldridge, Corinne: 9030
Elfenbein, Pamela: 2610
Eliopoulos, Elaine M.: 9010
Ellerby, Lisa: 6840
Ellington, Lee: 1320, 2070, 2540, 4530, 6310, 9000
Elliott, Christian: 9010
Elliott, Emily M.: 6830
Elliott, Michael: 2080, 6140
Ellis, Julie M.: 2160
Ellis, Julie: 4250
Ellis, Katrina: 4010
Ellis, Kirby B.: 9020
Elman, Alyssa: 4900
Elman, Jeremy A.: 6050, 6490
Elmore, Catherine E.: 3500, 6310
Elrod, Cathy: 9030
Elrod, Leigh: 2060
Elswick, R.K.: 6420
Elugbadebo, Olufisayo: 6100
Emdin, Abby: 9000
Emery-Tiburcio, Erin: 2030, 2560
Emlet, Charles A.: 4540
Empleo-Frazier, Ophelia: 5172
Emrani, Sheina: 6411

Endo, Yori: 5001, 6840
Eng, Cassy: 9000
Engeland, Christopher G.: 4610, 4620, 6290
Engel, Ilana J.: 6650, 6730
Engelman, Corinne D.: 2670
Engelman, Michal: 6710
Engel, Rafael: 6090
English, Tammy: 2470, 4570, 6170
Enguidanos, Susan: 3390, 4100, 6120, 6720
Ensrud, Kristine: 2290, 4080, 6320, 9030
Eoff, Elizabeth: 6130
Epel, Elissa: 3070, 3440
Epps, Fayron: 1170, 1380, 3240, 5160
Epstein, Mara M.: 2640
Eramudugolla, Ranmalee: 9000
Erich, Michele: 6350
Ericson, Tracy: 6540
Erlenbach, Emily: 9010
Ernsth-Bravell, Marie: 9020
Ernst, Joy S.: 6000
Ernst, Michael E.: 4320, 5083, 6320
Ersek, Mary: 1390, 1460
Ertle, Emilee: 6290, 9010
Erus, Guray: 4200
Escorcia, Wilber: 5001
Esiaka, Darlingtina: 6070, 6200, 6430
Eskandari, Anahita: 6170
Esmaeili, Roghayeh: 3620
Espejo, Edie: 4460, 5030
Espejo, Luis: 6840
Espeland, Mark A.: 3400, 3510
Espinosa, Oscar: 6120
Espinoza, Anna: 6770
Espinoza, Arturo I.: 9020
Espinoza, Sara E.: 4320, 6020, 6140, 6320, 6390
Esposito, Michael H.: 4110
Estabrooks, Carole A.: 2090, 4210, 4720
Estenson, Lilly: 9000, 9010
Estenson, Lilly H.: 3010
Estepp, Philip: 6780
Estus, Erica N.: 3780, 6400
Etchin, Anna: 2660, 6220
Evans, Daniel S.: 9010
Evans, Donald: 9010
Evans, Luke: 9010
Evans, Michele K.: 5060
Evans, Nate: 6740
Evelyn-Gustave, Allyson: 4680
Ewen, Heidi: 4520, 9030
Eyler, Lisa T.: 6050
Ezeamama, Amara E.: 4700

**F**

Fabelo, Humberto E.: 2700
Fabio, Casati: 4684
Fabius, Chanee: 4640, 6760
Fadel, Meghan: 6060
Fagundes, Christopher: 3481, 9010
Fahey, Margaret C.: 9000
Fairchild, Kaci: 6010
Fairchild, Thomas J.: 1250
Fairchild, Vickie: 4790
Fakha, Amal: 2090
Falkovic, Margaret B.: 1540
Falvey, Jason R.: 2580, 4220
Falzarano, Francesca: 2000, 6210, 6530, 6760
Fan, Erica K.: 3690
Fanfan, Dany: 4650
Fang, Tyson: 6310
Fang, Yuan: 6450, 6740
Farago, Fanni: 9020
Farah, Fatima: 6411
Farah, Kimberly: 2350, 3800
Farhat, Farah: 4340
Farina, Mateo: 1410, 1440, 2080, 3040
Farmer, Heather R.: 3440, 6290
Farnell, Yuhua Z.: 6010
Farrell, Barb: 6270
Farrell, Kurt: 6010
Farsijani, Samaneh: 3830
Fashaw-Walters, Shekinah: 2650, 4780, 6540
Fassi, Janelle E.: 1260, 6000
Faul, Anna: 1110
Faul, Jessica: 3000, 6010
Faulkner, Sandra: 4022, 9020
Faurot, Keturah R.: 4320
Fausto, Bernadette: 6070, 6200
Fauth, Elizabeth Braungart: 6410, 6560
Favez, Lauriane: 1530, 2090
Fazio, Sam: 4010, 4681, 6130, 9030
Federman, Alex: 6610
Fehland, Jess: 2160
Feitosa, Mary: 4600, 4810, 6010
Felber, Nadine Andrea: 6400
Felderhoff, Brandi J.: 1310
Feliciano, Leilani: 3115, 6170, 6820
Felix, Ashley: 2200
Felix, Holly: 6420
Felts, Adam: 6310
Feng, Guanggang: 6480
Feng, Qiushi: 1100
Feng, Xin: 6641
Feng, Zhanlian: 4350
Fennell, Gillian: 4580, 6440
Fennema-Notestine, Christine: 6050, 6490
Fenske, Diane L.: 9010
Fenstermacher, Erika A.: 9020, 9030
Fenton, Laura: 6580
Ferdows, Nasim B.: 4420
Ferguson, Giselle: 6550
Ferguson, Verna: 6720
Fernandez Cajavilca, Moroni: 9000, 9010
Fernandez-Barrena, Maite: 9000
Fernandez-Zapico, Martin: 9000
Ferraguzzi, Gaia: 5010
Ferraro, Kenneth F.: 4230, 5060, 6290, 6380, 6810
Ferreira Leitao Azevedo, Renato: 4190
Ferreira, Katelyn: 1350
Ferris, Chloe: 3400
Ferrone, Christine F.: 6770
Ferrucci, Luigi: 1470, 4200, 4230, 4340, 4600, 9010
Fetting, Hannah: 9020
Fick, Donna M.: 3720, 6270, 9030
Fielding, Roger A.: 3830
Field, Noelle: 2010
Fields, Beth: 1340, 4310, 6530
Fields, Noelle L.: 1080, 6060, 6530, 6630, 9030
Fiestosa, Mary F.: 4810
Filec, Sarah: 4681
Fillingim, Roger: 6510, 9020
Finan, Julia M.: 2050
Finch, Caleb: 9020
Finch, Holmes: 9010
Finch, Laura: 1230
Finegan, Kerry: 9030
Fine, Sydney: 9020
Fingerman, Karen L.: 1450, 2440, 3730, 6170, 6460
Finkel, Deborah G.: 3440, 6050, 9010, 9020
Finlay, Jessica M.: 1050, 2570, 3495, 4110
Finley, Erin: 1330
Finley, Margaret A.: 1120
Finnie, Dawne M.: 2160
Finsel, Julia: 6700
Fiocco, Alexandra J.: 4490, 6440
Fiori, Katherine L.: 3480, 6170
Fischer, Karen: 6110
Fisher, Abbey: 3520
Fisher, Kathleen: 1120, 5020, 9020
Fisher, Lauren: 6280
Fisher, Mary: 9000
Fish, Laura J.: 1540
Fisk, Calley: 9000
Fiske, Amy: 9000, 9010, 9020
Fiske, Sarah: 6740
Fiskum, Gary: 6641
Fitch, John: 6310, 9030
Fitzpatrick, Annette L.: 5010
Flaherty, Ellen: 4390
Flanagan, Jane: 2230
Flanagan, Margaret E.: 6411
Flatt, Jason: 2390, 3290, 3950
Fleischhacker, Alexander: 9030
Flessa, Sarah: 9000
Fletcher, Jason: 6110, 9000
Flett, Gordon L.: 4630
Flieger, Signe P.: 6360
Flood, Sarah: 6800
Flores, Eileen S.: 4691
Flores, Elena: 6180
Flores, Emily V.: 6290, 9030
Flores-Villavicencio, Maria Elena: 6290, 6380
Floyd, Alex: 1390
Flynn, Ann: 9000
Fogle, Stephen John: 1070, 2260
Foley, Kierra: 9030
Follis, Shawna: 9010
Fong, Joelle: 1100
Fong, Mei-Chia: 1190
Foppa Pedretti, Nicolo: 1350
Foradori, Megan: 1530
Ford, Ariel: 2130, 3060
Forman, Daniel E.: 4600
Forrester, Sarah: 1090
Fortinsky, Richard H.: 2150, 2510, 3310, 3680, 4280
Fortuna, Karen L.: 3481
Foster, D. Brian: 6411
Foster, Kristie: 4390
Fountain, William: 9010
Foutz-Lowe, Shelly: 9030
Fowe, Ibukun E.: 4020
Fowler, Michael: 4640

Fowler, NIcole R.: 6220, 6390, 6450, 6470, 6580, 9000, 9030
Fox, Aimee: 2250, 2280, 3930
Fox, Jessica: 4240
Framil, C. Victoria: 9000
Francisco, Raia: 9020
Francis, Erica: 3520
Francis, Heather: 6820
Francis, Howard W.: 9030
Francis, Jess: 1240
Francis, Sarah L.: 9000
Frangiosa, Theresa: 2160
Franke, Kevin: 3460
Frank, Robynn: 1480
Franz, Carol E.: 6050, 6490
Franzosa, Emily: 1040, 1160, 3660, 6530, 6610
Frase, Robert T.: 1290, 6520
Fraser, Sarah: 4340
Fratoni Souza, Kristin N.: 2350, 3780
Fratoni-Souza, Kristin: 6400
Frazier, Cleothia: 6600
Frazier, Hilaree: 6411
Frazier, Jefferson: 3481
Frechman, Erica: 1180
Freddolino, Paul P.: 4023, 4770, 6060
Fredericksen, Jessica: 6280
Fredrickson, Barbara: 4850
Fredrika, Opur: 1170
Fredriksen Goldsen, Karen I.: 1320, 4540
Freedman, Vicki A.: 4700, 5070
Freeman, Shannon: 2210, 2600, 3450, 6270
Freiria, Carol: 9020
Freitas, Sam: 6840
Frey, Joseph J.: 6120
Frey, Katelyn T.: 9000
Frey-Law, Laura: 6220
Friberg Felsted, Katarina: 1370, 4520
Frichembruder, Karla: 6100
Friedman, Elliot: 6080, 6790
Friedman, Esther: 6690
Friedman, Naomi: 9020
Friedmann, Erika: 9020
Friedrich, Anna-Carina: 4300
Fried, Terri: 3360
Friis, Marlene: 6830
Fritschi, Cynthia: 6080, 6300, 6780
Fritz, Heather A.: 6650
Fritz, Julie: 1120
Fritz, Michele C.: 4770
Fritz, Roschelle L.: 6420
Fritzson, Emily: 9000
Froberg, Ruthann: 4890
Fruhauf, Christine A.: 2250, 2280, 3710, 3930
Fry, Liam: 6260
Fuentes-Casiano, Edna: 6160
Fugate-Whitlock, Elizabeth: 4520, 6640
Fujiwara, Yoshinori: 6320, 6710
Fukui, Chie: 4440
Fukui, Sadaaki: 9010
Fulda, Kimberly: 6770
Fullen, Matthew C.: 4060, 6250
Fuller, Heather: 3480, 6230, 9020
Fuller, Kristi: 6090
Fuller-Thomson, Esme R.: 4225, 6200
Fulmer, Terry: 4890
Fulop, Tamas: 6140
Fulton, Janet: 9030
Fu, Mingqi: 6450
Funderburg, Allesia: 3550
Fung, Nicole Long Ki: 2480
Fung, Celeste: 1510
Fung, Helene H.: 2480, 3250, 6250
Fung, John Chye: 3450
Fung, Sai-fu: 6350, 6530
Fu, Rong: 6350
Fu, Xiaoyu: 6520

G

G. Cuevas, Pearl Ed: 3310
Gabor, Helena E.: 1220
Gabriel, Kelley Pettee: 2290
Gad, Dustin: 9000
Gadkari, Gauri: 5081
Gagnon, Christine: 6570
Gaillard, Trudy: 6140
Galambos, Colleen: 1460, 4250, 4830, 6750
Galea, Sandro: 6830
Gale, Kathleen S.: 9020
Galera, Leonor: 9010
Galik, Elizabeth: 3650, 3830, 4760, 6560
Gallagher, Elizabeth A.: 6480, 6830
Gallagher-Thompson, Dolores: 1330, 6430, 6750
Gallagher, Virginia T.: 9010
Gallant, Noelle: 5172
Galligan, Catherine J.: 2710
Gallo, Joseph: 6800
Gallo, Linda: 9000
Galloway, Rebecca: 4150
Galo, Caroline: 9030
Galucia, Natalie: 3800
Galvin, Angeline: 4810
Galvin, James E.: 1030, 6070, 6720
Gamaldo, Alyssa A.: 2380, 3270
Ganci, Aaron: 9000
Gan, Daniel Rong Yao: 3450, 4590, 4860, 6290, 6500
Gandy, Maribeth: 9000
Gan, Siqi: 4460, 5030
Gao, Ran: 6110
Gao, shuhong: 6110
Gao, Sibo: 6460
Gao, Xianqi: 9010
Gao, Xiaojun: 6430
Garbarino, Jason: 1070
Garbarino, Lisa: 2490, 6060
Garcia Morales, Emmanuel: 3560, 4050, 6710
Garcia, Catherine: 1480, 4225, 6200
Garcia, Giovanni: 6140
Garcia, Linda: 6150, 6210
Garcia, Marc A.: 2510, 4420
Garcia Morales, Emmanuel: 4050
Garcia, Odalis: 6580
Garcia, Patricia: 6140
Garcia, Sandra: 1330, 6420
Garcia, Y. Evie: 9030
Gardenhire, Douglas S.: 6780
Gardier, Dawn: 6140
Gardiola, Andrea: 6230
Garell, Catia: 6230
Garr, Andrea: 6750
Garren, Patrik A.: 6100
Garrido, Melissa: 3200
Garrison, Betsy: 6200
Garrity, Katherine: 6270
Garron, Angelina: 4570
Gartlan, Olivia: 6790
Garvan, Cynthia: 2300
Gary, Katharine: 6060
Gasdaska, Angela: 3160, 3640, 4350
Gaspar, Phyllis M.: 9020
Gasser, Susan M.: 1200
Gassoumis, Zach: 3010
Gaster, Barak: 5010
Gately, Megan E.: 1040, 6420
Gatz, Margaret: 3510, 6050
Gaugler, Joseph E.: 1050, 1170, 1480, 1560, 2130, 2160, 3020, 3310, 4060, 4220, 5160, 6060, 6070, 6540, 9000
Gaurke, MaryKatherine A.: 4180
Gautam, Ramraj: 2610
Gayda, Mathieu: 6570
Gay, Emma: 4600
Gaziano, J Michael: 3600
Geary, Carol R.: 4590
Gebeyaw, Getachew: 6330
Gee, Gilbert: 4780
Gee, Nancy R.: 9020
Gehlot, Vijay: 9030
Geissal, Erik: 6610
Geisser, Sophia R.: 6110
Gellert, Paul: 6110
Gellis, Zvi: 4750
Gendron, Tracey: 3670, 9000, 9010
Geng, Jing: 6210, 6750
George, Joanna: 6390
George, Kristen M.: 3600
George, Mollie J.: 6090
George, Rosmy: 4870
Gepner, Adam D.: 5050
Gerhardus, Ansgar: 5170
Gerhart, Olga: 4660
Gershon, Robyn: 6090
Gerstorf, Denis: 1010, 1020, 3070, 3150, 4570, 6210, 6390, 6580, 6641
Getchell, Nancy: 4021
Getz, Marjorie A.: 6000
Ghaemmaghami, Sina: 5091
Ghaiumy Anarsky, Reza: 4020, 9030
Ghanbari, Mohsen: 5170
Ghattas, Catherine: 6641
Ghazi Saidi, Ladan: 1270, 3780
Ghiasi, Akbar: 4820
Ghimire, Saruna: 6750, 9010
Ghosh, Bhaswati: 2501, 6840
Ghosh, Dibendu: 6350
Ghosh, Ritam: 5090
Giasson, Hannah L.: 2220, 6640
Gibbons, Laura E.: 3510
Gibbs, Lisa: 2070
Gibson, Allison: 1380, 2460, 4790, 4870, 6060, 6720
Gibson, Regina V.: 6130, 6180
Gibson, Samantha: 9030
Gieniusz, Marzena: 9000, 9010
Giffuni, Jamie: 5050
Giles, Sadie: 6210, 6750

Gillespie, Suzanne: 2660
Gilligan, Megan: 1290, 2440, 4070, 4460, 6520
Gills, Joshua: 6450, 6510, 9000
Gill, Thomas M.: 2760, 5100
Gilmartin-Thomas, Julia F.: 6320
Gilmore-Bykovskyi, Andrea L.: 2160, 3750, 4610, 6310
Gilpin Macfoy, Fiona: 6170
Gilsanz, Paola: 3600
Gimenez-Santana, Alejandro: 6200
Gimie, Afnan M.: 4220
Gimm, Gilbert: 3130, 3870, 4510
Gincel, Dan: 6320
Ginsburg, Liane: 4720
Giordani, Bruno: 4700, 6290
Giorgadze, Nino: 6840
Girdwood, Tyra: 3482
Girling, Laura: 9020
Girma, Meron: 9020
Girouard, helene: 6740
Gitlin, Laura N.: 2150, 2420, 2610, 3310, 4870
Giuliani, Cristina: 6010
Gladyshev, Vadim: 1140
Glaser, Kathryn: 6430
Glasheen, William: 3540
Glassner, Ashlie A.: 2420
Gleason, Kathy: 3360
Gleason, Shayna: 3100, 6700
Gleissner, Laura: 3610, 6380
Glenn, Jordan: 6450, 6510, 9000
Glenn, Lee: 9020
Glicksman, Allen: 4360
Glover, Sabrina: 6770
Gluck, Mark: 6070, 6200
Glueckauf, Robert L.: 4681, 9030
Glymour, Maria: 3510, 3690
Glynn Hames, Alexandra: 9010
Glynn, Nancy W.: 4120, 4600, 4810, 9030
Gmelin, Theresa: 9030
Gnanasekaran, Gowrishankar: 6270
Gobbens, Robbert: 4550
Godde Chrisco, Kanya: 6080
Godek, Eileen: 4023
Godina, Sara: 1050, 9030
Godsey, George A.: 6411
Goebel, Jan: 3070
Goetz, Vanaditz: 1270
Goh, Kim Huat: 4220
Goh, Veronica Shimin: 2150
Goidel, Kirby: 2250
Goldberg, Allon: 6510, 9030
Goldberg, Anne: 6210
Goldberg, Jaime: 6520
Golden, Jeannette: 2690
Golden, Robyn L.: 2560
Golden, Shelley: 5060
Golding, Hannah: 1470
Goldman, Alyssa: 5150
Goldman, Roberta E.: 6770
Goldstein, Daniel R.: 3241
Goldstein, Jenna: 4690
Goler, Timothy: 6200
Golub, Justin S.: 3600
Gomes Da Rocha, Carla: 6380
Gomez, Rowena: 6010, 6170, 9020
Gonyea, Judith G.: 4430, 6730
González, cesar: 6330
González, Hector: 9000
González, Kevin: 9000
Gonzales, Ernest: 3890, 6630, 6680
Gonzalez Bautista, Emmanuel: 4290
Gonzalez, Alexander: 4510
Gonzalez, Joanna: 6140
Gonzalez-Lopez, Paloma M.: 2160
Gonzalez-Prado, Roberto: 9000
Gonzalez, Richard: 2420, 2440, 4010, 4700
Gonzalez, Stephanie: 9020
Goode, Collette: 6270
Goodman, Michael: 3290
Goodpaster, Bret: 3340, 4340, 4600, 9010
Goodwin, Paige: 9030
Goodwin, Rebecca M.: 4530, 6310
Goodyear, Kimberly: 4580
Goo, Kelly: 9000
Gooneratne, Nalaka: 6600
Gopalakrishna, Ganesh: 9000
Gopalakrishnan, Chandrasekar: 6110
Gopalan, Shyamie: 4620
Gopher, Daniel: 6420
Gorbenko, Ksenia: 1160, 3660, 6610
Gorbunova, Galina: 1000
Gorbunova, Vera: 2501, 6840
Gordon, Barbara: 1110, 1320
Gordon, Kristina C.: 5090
Gordon-Larsen, Penny: 2080
Gordon, Peter: 9000
Gordon, Stacey: 6630
Gore, Janelle: 1170
Gore, John: 4461
Gore, Rebecca J.: 2710
Gorospe, Myriam: 1470
Goss, David: 4790
Gossner, Jacob: 6410, 6560
Gotanda, Hiroshi: 5083
Gothard, Sarah: 4790, 6080, 9000
Gothelf, Isabel: 1320
Gothe, Neha P.: 5082, 5130, 9010
Goto, Jun: 6240
Gottesman, Elaine: 4900
Gottesman, Eve: 6220
Gough Courtney, Margaret: 6080
Gould, Christine: 6310
Goulet, Joseph: 6770
Gourley, Amy N.: 6810
Gouskova, Natalia: 3482, 3510, 9000
Gowda-Sookochoff, Rory E.: 9000
Gowin, Lizzie: 9000
Goyal, Parul: 6740
Gozalo, Pedro: 3200
Gräßel, Elmar: 4300
Grünjes, Carlotta: 1020
Graber, Meghan: 5001
Grace, Rachel: 6780
Grady, Alexandra: 9020
Grady, James: 2150
Graf, Allyson S.: 4520
Graff, Sarah: 1200
Graf, Sylvie: 2480
Graham, David: 1280
Graham, Eileen K.: 2140, 2620, 4620
Graham-Engeland, Jennifer E.: 4620, 6290
Graham, Helen L.: 9010
Graham, Lyndsey N.: 2140
Graham, Robert J.: 2020
Granados Unger, Federico: 4120, 6780
Grant, Claire: 4010, 6130, 6310
Granville, Lisa: 6420
Graton, Margaret: 9020
Graupner, Jeffrey: 6220
Gravenstein, Stefan: 9030
Graves, Jessica: 9010
Graves, Jillian: 9010
Gray, Ja'Lynn: 3330
Gray, Michelle: 6450, 6510, 9000
Gray, Nicholas: 6290, 9020
Gray, Taylor R.: 6120
Greaney, Jody: 3730
Green, Aval-Na'Ree S.: 4150
Greenberg, Phyllis A.: 6000, 6220
Greenberg, Sherry A.: 1120, 1250, 1530
Greenberg, Steven: 3580
Greenfield, Emily: 1170, 2010, 2570, 4860, 5080
Green, Geoff: 6320
Greenhalgh, Ella: 9010
Green-Harris, Gina: 3750
Green, Shoshana: 9000
Greer, Faith: 4520
Gregor, Neil: 4260
Greiner, Chieko: 6100, 6110, 9000
Grelle, Kaitlin: 6160
Grenier, Amanda: 6640
Grewal et al., Karl S.: 9000
Grey, Tom: 9010
Grieb, Suzanne: 2690
Griffin, Angel: 4681
Griffin, Joan M.: 2000, 2160, 3390, 6530
Griffin, Meridith: 3620
Griffin, Patrick: 1140
Griffin, Shannon: 2020
Griffin, Terry: 1420
Griffith, Christopher X.: 6820
Griffith, Eric E.: 6210
Griggs, Stephanie: 2200
Grillo, Anthony: 6840
Grimes, Amanda: 9010
Grimm, Kevin E.: 6490
Griswold, Michael: 2670, 3110, 4200, 6780
Grošelj, Darja: 1210
Groarke, John: 3830
Groden, Sheryl: 4023, 9030
Groen-van de Ven, Leontine: 4550
Groenvynck, Lindsay: 1160
Gross, Alden L.: 2200, 2270, 3510, 6740
Grossberg, George T.: 6260
Gross, David: 1380
Gross, Evan: 6480
Grossman, Robin: 4225, 6200
Groulx, Mark: 3450
Groves, Elizabeth: 9030
Growdon, Matthew E.: 5030, 6110
Growney, Claire M.: 2470, 6170

Grudzien, Adrienne: 9000
Gruenewald, Tara L.: 6670
Grühn, Daniel: 3190
Grund, Ethan: 3241
Gruneberg, Audrey: 5000
Gruss, Valerie: 3500
Gryglewicz, Kim: 6350, 9020
Guasco, Rick: 4023
Guerbaai, Raphaelle Ashley: 6270
Guest, Aaron: 3420, 3710
Gugala, Zbigniew: 1120
Guggenheimer, Joshua D.: 9010
Gugliucci, Marilyn R.: 1370, 3800, 6100, 6270
Guidry, Hayden: 2501, 6840
Guillory, Logan A.: 6410
Gu, Lin: 5050
Gunn, Grayson: 1170
Guo, Ellen: 9000
Guo, Fangqi: 3440
Guo, Man: 1090, 1130, 6500
Guo, Xueying: 6770
Guo, Yi: 4040
Guo, Yingqi: 9030
Gupta, Riddhi: 2550, 6440
Gurinovich, Anastasia: 2501, 4810, 6010
Gurlu, Merve: 9020
Gur-Yaish, Nurit: 1160, 2680
Gusmano, Michael K.: 1260, 2040, 4500
Gustafson, Deborah: 3760
Gustavson, Allison: 3310
Gutierrez, Angela: 6140
Gutierrez, J Antonio T.: 5050
Gutierrez, Mariela: 4225, 6300, 9010
Gutierrez-Ramirez, Paulina: 4190
Gutkin, Michael: 6840
Gutman, Gloria M.: 1210
Gu, Xiuzhu: 6700
Guzzardo, Mariana T.: 6210
Gwee, Xinyi: 4023

**H**

Haapala, Heidi: 2500
Haardoerfer, Regine: 9020
Haas, Allen: 5100
Haase, Claudia: 3150, 4570, 4850
Haas, Steven A.: 1090
Habegger, Alex: 6740
Habermann, Elizabeth: 6450
Habiballa, Leena: 3241
Habib, Semper: 4555, 6380
Hackett, Sara E.: 1490, 2070, 2720, 4465
Hackney, Madeleine: 9010
Haddad, Yara: 4100
Hadley, Evan: 4810
Hado, Edem: 2650
Hageman, Sally: 6400
Hagen, Erika W.: 4560
Hager, David: 6140
Haggar, Katherine: 4860
Hägg, Sara: 3590
Haghighat, Misha D.: 4170
Hagler, Donald J.: 6050
Hahne, Jessica: 4480, 6520
Haibach-Beach, Pamela: 2530
Haileyesus, Tadesse: 4100
Hajdar, Melisa: 6230
Hakimjavadi, Ramtin: 1510
Halaszynski, Jaime J.: 9000
Haley, William E.: 2720, 3910, 4650, 6810
Halladay, Christopher: 3130
Hall, Charles: 4700
Hall, David: 9010
Hall, Katherine S.: 2660, 4260, 5050, 6170
Hall, LaToya N.: 6480
Hall, Marissa: 5060
Halpin, Sean N.: 2640, 3550
Halter, Colt M.: 6290
Haltermann, William: 3640
Halvorsen, Cal J.: 1080, 9030
Ham, Carolyn R.: 1060
Hamel, Kate: 6250
Hamermesh, Daniel: 2410
Hamers, Jan P.: 1160, 2090, 5100, 6560
Hamersky, Carol: 9000
Hamilton, Jill: 9020
Hamilton, Lucas J.: 2400, 3570, 6810
Hamilton, Tonua: 1120
Hamlat, Elissa: 3440
Hamler, Tyrone: 6820
Hamm, Callie: 6570
Hamm, Jeremy: 6580
Hammond, Robert: 3540
Han, Benjamin H.: 2245, 2760
Hancock, Cynthia R.: 4520
Hancock, David W.: 6480
Hancock, Laura M.: 2670
Handing, Elizabeth: 3115, 3250
Handlery, Reed: 6740
Han, Duke: 1430, 4070, 6580, 9030
Handzo, George: 6270
Hanes, Douglas W.: 6200, 6680
Han, Gang: 6310, 9030
Han, Gyounghae: 2440, 3220, 6520, 6670
Han, Hae-Ra: 3330, 3560, 4680
Han, Heather: 4520
Hanis, Jen: 6440
Han, Ling: 6770
Hanlon, Alexandra L.: 9000
Hanlon, Samuel: 6270
Han, Molly: 1570
Hannan, Marian T.: 2290
Hannan, Mary: 6411
Han, Sae Hwang: 1100, 3730, 6170, 6520
Hansen, Jared: 1330
Hansen, Lissi: 6830
Hansen, Thomas: 2410
Han, Seung Yong: 9020
Hansmann, Kellia J.: 5172, 9020
Hanson, Andrew: 6450
Hanson, Heidi A.: 4530
Hanson, Paige: 2520
Hansson, Isabelle: 4620
Han, Sua: 6170, 6780
Hantke, Nathan: 1340
Happ, Mary Beth: 3360, 5090, 6310
Haque, Raza: 2210, 9020
Hardiman, Madison: 2501
Harding, Alyson: 3690, 9030
Hardin-Sigler, Kristen: 6160
Hardman, Alisha: 6280
Hare, Joshua M.: 6320
Hargraves, J. Lee: 9020
Haring, Nicole: 4670
Haring, Rodney: 6430
Harkins, Kevin: 4461
Harlow, Sioban: 2080, 6140
Harnish, Autumn: 4510
Harrington, Candace C.: 2150
Harrington, Erin E.: 6290, 9020
Harrington, Karra D.: 3250, 6790
Harris, Daniel: 9000
Harris, Jordan: 6090
Harris, Maurita T.: 3670, 6300
Harris, Melissa L.: 6420, 9000
Harris, Melodee: 6180
Harrison, James: 9000
Harrison, Krista L.: 5030
Harrison, Sean L.: 6390
Harrison, Stephanie L.: 2290, 4080
Harrison, Theresa: 3740, 9030
Harrison, Tracie C.: 3483
Harris, Rebekah: 4260, 5050
Harris, RianSimone: 1450
Hartford, Emily: 9030
Hartman, Mikael: 3390
Hartmann, Christine W.: 1040
Hartman, Sheri J.: 9000
Hart, Sara: 9000
Harvey, Idethia S.: 6650
Harzelak, Jaroslaw: 4600
Hasan, Danish: 6180
Hasan, Maryam: 9000
Hasche, Leslie: 2310, 4240, 4430
Hasdemir, Dilara: 1150
Hashemi, Mahdi: 3200
Hasper, John: 5091
Hassan, Mahdi: 6740
Hass, Zach: 4900, 6630
Hastings, Susan N.: 5083
Hata, Toshiki: 6710
Hathaway, Zachary: 5020, 9020
Haughtigan, Kara: 9030
Hauser, Elliott: 1570
Hausman, Hannah: 1470
Haverhals, Leah M.: 2660, 2720, 6210
Hawes, Frances M.: 5140, 6140
Hawkley, Louise: 1230, 6440
Hawley, Chelsea: 4770
Hawthorne-Crosby, Brieanna: 9010
Hayashida, Cullen T.: 6580
Hayat, Shabina: 2270
Hayden, Kathleen M.: 2670
Hayduk, Leslie: 4210
Hayes, Emily J.: 6580
Hayes, Michael: 3160
Hayes, Susan L.: 5081
Haynes, Laura: 9010
Hays, Garrett: 5091
Hayslip, Bert: 6750
Hazimeh, Fatima: 3860
Hazuda, Helen: 6140
Hazzan, Afeez A.: 2350, 2530, 6280
Hazzan, Joyce: 6280
Head, Elizabeth N.: 2490
Head, Robert: 6170
Heatwole Shank, Kendra: 6300
Heavner, Mojdeh: 6350

He, Daqing: 6150
Hedge, Eric: 4120, 6780
Heffernan, Kristin: 2350
Heffner, Michelle: 6090
Hege, Adam: 4820
Heid, Allison R.: 6130
Heideman, Kristin: 6210, 6411
Heij, Ward: 1120
Heilbrunn, Emily S.: 3520
Heilman, Keri: 1550
Heimler, Stephanie R.: 4461, 6010
Hein, Maria: 2520, 5010
Heintz, Hannah: 2030, 2660
Heinz, Melinda S.: 3610, 6380
Heisel, Marnin J.: 4630
Heislbetz, Claus: 1270
He, Linchen: 4330
Helppie-McFall, Brooke: 9020
Helsinger, Abigail L.: 4555, 6130, 6670, 6710
Hemingway, Dawn: 4020
Henage, Cris: 1110
Henderson, Charles R.: 4225, 6180
Hendricksen, Meghan: 1090, 1530
Hendrie, Hugh C.: 9020
Hendrix Rogers, Anita: 1530
Hendry, Phyllis: 9030
Henegan, James: 3110, 4200
Henley, Laura: 4790
Henneberry, Kathleen: 9020
Henne, Jeff: 1320
Hennessa, Alexandra: 2010, 6760
Henrich, Christina M.: 3115
Hensley, Mark C.: 9020
Hensley, Robert B.: 6810
Heo, Jinmoo: 6380
Hepburn, Kenneth: 3240, 4030, 5160
Hepple, Russell T.: 3340, 6780, 9010, 9020
Heppner, Alexandra: 2340, 4360, 6130
Heppner, Marie-Marleen: 1270
Herbert, Brittney-Shea: 6220
Herbert, Christopher: 3900
Herd, Pamela: 2400
Hermanns, Melinda: 9010
Herman, William: 2080
Hernandez Chilatra, Jessica A.: 9000
Hernandez de Jesus, Alma V.: 9000
Hernandez, Manuel E.: 6740
Hernandez, Olivia: 5020
Hernandez-Swift, Karla: 4010
Herr, Keela: 6310
Herrmann, Francois: 6380
Herrmann, Lynn K.: 6160
Hersch, Gayle I.: 6760
Hertzog, Christopher: 6710
Herz, Jasmin: 4461
Heslop, Thomas: 3000
Hess, Rachel: 1120
Hess, Thomas M.: 2480
Heston-Mullins, Jennifer: 3180
Heussner, Pia: 5110
Heyn, Patricia C.: 1060, 3350, 3410, 4400, 4860, 9030
Hiatt, Shirin O.: 4790, 6830
Hicken, Bret: 9020
Hickman, Louis: 2280
Hickman, Ronald: 2200
Hickman, Susan: 4100, 6770, 9030
Hicks Patrick, Julie: 6140, 6200, 9000
Hicks, Hilary J.: 6570
Hiddemann, Wolfgang: 5110
Higbie, Anna: 6390
Higgins-Chen, Albert T.: 1140
Hill, Andrea M.: 5173
Hill, Carl V.: 9020
Hillhouse, Andrew: 6010
Hill, Linda: 5171, 9030
Hillman, Vanessa: 9000
Hill, Patrick L.: 2430, 4620, 6580
Hilmer, Sarah: 2090
Hilsabeck, Tyler AU: 6010
Hines, Anika L.: 4680
Hinrichsen, Gregory: 4250
Hinton, Ladson: 3310, 4700
Hinyard, Leslie: 6720
Hiroto, Kimberly: 3420
Hirsch, Jen: 4270, 9000
Hirschman, Karen: 6200, 6280, 9000
Hirst, Roxana: 4150
Hisamatsu, Yasushi: 6450
Hitchcock Noel, Polly: 1330
Hladek, Melissa: 2200, 4680
Hoben, Matthias: 4210, 4720, 5010, 6760
Hobgood, Sarah: 6320
Hochberg, Marc: 6780
Hochgraeber, Iris: 3090
Hodes, Richard: 3080, 3280
Hodgdon, Barbara T.: 6750
Hodgson, Nancy: 3310, 3680, 3930, 6110, 6300, 6390, 6510, 6600, 9000, 9030
Hoe, Deborah: 3390
Hoey, Jesse: 9000
Hofer, Molly: 5082, 5130
Hofer, Scott M.: 1020, 2140, 3440, 3510, 3590, 5084, 6290, 6440, 6580, 9020
Hoffman, Adar: 6580
Hoffmann, Anna-Louisa: 3090
Hofmann, Brittany: 3480
Hoft, Daniel: 3600
Hogans, Beth B.: 6220
Hogentogler, Ellie: 3520
Holaday, Louisa: 4130
Holden, Richard J.: 3060, 6270, 9000
Holden, Samantha: 9000
Holland, Diane: 3390, 6110
Holland, Elizabeth: 9000
Hollander, Matthew: 9020
Holland, Peter: 9000, 9010
Holland, William: 9030
Holle, Bernhard: 3090
Holley, Lyn M.: 4710, 6700
Hollingshaus, Michael: 4530
Hollingsworth, Emily: 6270
Holloway, Jeremy C.: 6370
Holmes, Ann: 6760
Holmes, Ashleigh: 6280
Holmes, Rachel: 4290
Holstad, Marcia: 3760
Holtgrave, Peter: 6060
Holt, Heidi L.: 2490, 6060
Holt-Lunstad, Julianne: 6790
Holtzer, Roee: 3250, 4340
Homan, Patricia: 6180, 9020
Honda, Miwako: 6000
Hong, Chelin: 6420
Hong, Joanna H.: 3730
Hong, Michin: 3220, 6120, 6160
Hong, Seunghye: 3220
Hong, Soyun: 6400
Hong, Sungjae: 1240, 9030
Hood, Nancy: 3520
Hoogeboom, Thomas: 1120
Hooker, Karen: 1080, 2280, 3490, 5000
Hooper, Savannah: 6020
Hooyman, Nancy: 6260
Hoppmann, Christiane: 1020, 2480, 3150, 6210, 6390, 6580
Hoque, Masudul: 3720, 6060
Horhota, Michelle: 6640
Hornbuckle, Lyndsey: 4030
Hornstein, Erika: 4680
Horstman, Molly J.: 4683, 6320
Hossain, Lubna: 3240
Hosseini, Maryam A.: 3880
Hosseini, Shera: 6000
Hostetler, Andrew J.: 2610
Hougaard, Ben: 9020
Hougaard, Benjamin T.: 6170
Hough, Katrina: 2690
Hou, Su-I: 1480, 2290, 4450
Hou, Yifei: 6520
Howard, annie Green: 2080
Howard, Marjorie: 9000
Howard, Michelle: 6000
Howe, Judith L.: 3420, 6220
Howell, Abigail: 1380
Howell, Britteny M.: 2260, 6270
Howington, Marshall: 6010
Hoy-Ellis, Charles: 4540
Hoyle, Jessica N.: 2050
Hryhorenko, Jennifer: 5091
Hsieh, Jessica: 6230, 6480, 6690
Hsu, Hui-chuan: 6800
Hsu, Jean W.: 2241
Hua, Cassandra L.: 4465, 5081
Huang, Alison: 2530
Huang, Jing: 4080
Huang, Liming: 6200, 6300
Huang, Pai--Chuan: 4450
Huang, Shijiao: 6010
Huang, Shuo: 4730
Huang, Weiliang: 6641
Huang, Wenxuan: 3260, 6730
Huang, Xiuxiu: 6150
Huang, Ying: 3550
Hubbard, Jennifer L.: 1110
Hubbard, Zachary L.: 3690
Huber, Jessica: 9030
Huber, Lesa: 6770
Hubner, Sarah: 6090, 6390
Hudgins, Kristen: 2020
Hueluer, Gizem: 1020
Huffman, Kim: 2241
Huffmyer, Mark: 6060
Hufnal, Shelby: 5020, 9020
Huggins, Madison L.: 3500
Hughes, Anne K.: 1400, 4770
Hughes, Brian P.: 2501, 6270
Hughes, Brian: 6840
Hughes, Jaime M.: 4770
Hughes, Kelly L.: 4350
Hughes, M. Courtney: 6750
Hughes, MacKenzie L.: 6710

Hughes, Matthew L.: 2750
Hughes, Robin E.: 1530
Hughes, Susan: 4800
Hughes, Tiffany F.: 1280
Hughes, Timothy: 9000
Hughson, Richard: 4120, 6780
Huh, Jimi: 5140
Huis in het Veld, Judith: 4550
Hu, Jie: 9020
Hu, Jinxiang: 4030
Hulko, Wendy: 4020
Hull, James: 6690
Hu, Luxin D.: 4060
Humber, Marika B.: 1330, 6310
Hu, Mei-Chen: 3600
Hu, Mengyao: 4700, 5070, 1540
Hung, Chang-Chiao: 6140
Hung, Jui-Ying: 4682
Hung, Lillian: 1550, 3450, 4590, 9000, 9010
Hung, William: 1040, 1350, 6320
Hunt, Angela: 6540
Hunter, Melissa: 3640, 4350
Hunt, Lauren: 3680, 4460, 5030
Huo, Meng: 3730, 4170, 4460
Huppert, Theodore J.: 4340
Huque, Md Hamidul: 9000
Hu, Rita X.: 1290, 2220, 6230
Hurley, Shannon: 9000
Huseth-Zosel, Andrea: 3480, 9020
Hussaini, Husna: 4160, 9020
Hussain, Sultana Monira: 4683, 5083
Hu, Wangyang: 4023
Hu, Xujing: 6110
Huynh, Grace: 4680
Hu, Yuanyuan: 6230
Hu, Yue: 3250, 6250
Hwang, Ann: 6140
Hwang, Charles: 5001
Hwang, Ula: 5100
Hwang, Woosang: 1450, 6520
Hwang, Yeji: 5020, 6300, 6470, 9020
Hynes, Mary T.: 6380
Hyun, Jinshil: 2140, 9010

I

Iaboni, Andrea: 9000
Iacob, Eli: 3500
Iaconi, Alba: 4210
Ibrahim-Bacha, Kareem: 6140
Ibuka, Yoko: 6300
Ickert, Edmund: 1280
Igarashi, Heidi: 6210
Iglesias, Pablo: 6411
Ihara, Emily S.: 2260, 3130, 4510, 6280
Iijima, Katsuya: 6240
Ikeda, Naoki: 4440
Ikeuchi, Tomoko: 6390, 6400, 6580
Ikram, Arfan: 5170
Ilardo, Joan: 2210, 6180
Ilavarasan, Abinaya: 6830
Ilori, Folasade: 6170
Imamura, Tomoaki: 2580
Im, Eun Ok: 9030
Imus, Philip: 1010
Infante, Kimberly: 9020
Infurna, Frank: 4060, 6490
Ingeborg Berg, Anne: 4620
Ingle, Pilar: 4240, 4650, 6720
Ingram, Cory: 3390
Ingvalson, Stephanie N.: 4220
Inman, Christina: 6840
Inman, Morgan: 6250
Inoue, Megumi: 2260, 3200, 4510
Insel, Kathleen C.: 4190
Intharakasem, Sirirat: 3840
Intrator, Orna: 1330, 6320, 6420, 4683
Intrieri, Robert C.: 9030
Iny, Nicole: 6100
Inzitari, Marco: 6450
Iob, Eleonora: 3210
Irani, Elliane: 2200, 3630, 6410
Irimia, Andrei: 9000
IRIMIA, ANDREI: 9000
Isaacowitz, Derek: 2330
Isaacs, Lauren: 4240
Isaranuwatchai, Wanrudee: 9030
Ishado, Emily: 1150
Ishioka, Yoshiko: 6580, 6810
Ishitake, Tatsuya: 6450
Islam, Farhana: 3260
Islaya, Cesz: 6210, 9030
Israel, Sarah: 6790
Issakainen, Mervi: 2460
Itoh, Sakiko: 6390, 6400
Ittermann, Till: 1010
Iveniuk, James: 2550, 6440, 6550
Iwamoto, Miyuki: 6000
Iwarsson, Susanne: 4220
Iwasaki, Yoshitaka: 6170
Iyer, Lakshmi: 4820
Iyer, Sowmya S.: 6310

J

Jablonski, Rita: 1390
Jaboob, Saida M.: 6830
Jacelon, Cynthia: 6740, 9030
Jack, Jr., Clifford: 2670
Jackson, Eva M J: 2490, 3510, 4120, 6060, 6280
Jackson, Kathleen: 2330
Jackson, Kathryn: 4620
Jackson, Piper: 2210
Jackson, Rebecca: 2200
Jacobs Dodson, Lindsey: 6220
Jacobs, Christine: 3600
Jacobs, Jeremy M.: 6610
Jacobs, Josephine C.: 1330
Jacobs, Mary Lindsey: 2030
Jacobson, Lauren: 9000
Jacobson, Mireille: 3200, 9010
Jahn, Georg: 6250
Jahoor, Farook: 2241
Jain, Akanksha: 6430
Jain, Felipe A.: 4190
Jain, Jennifer: 3540, 9030
Jain, Mohit: 4461, 6010
Jain, Shalini: 9000, 9010
Jain-Washburn, Ethan: 6010
Jajodia, Anushka: 3330
Jaldin, Michelle: 6570
Jallow, Fatoumata: 6770
James, Bryan D.: 2140, 4620
James, Dara: 9000, 9020
James, Dawn: 6740
James, Jennifer: 5120
Jang, Jae Young: 6450, 6510, 6740
Jang, Jieun: 5084
Jang, Jiyoon: 9000
Jang, Yuri: 2420, 3160, 4370, 5000, 6180, 6490, 6810
Janke, Megan C.: 3270
Jankowski, Thomas B.: 4590, 6400
Jansen, Carl-Philipp: 2450
Jansen, Taylor: 3100, 3320, 6090, 6510
Janssen, Daisy: 6270
Janssen, Leah: 9030
Jao, Ying-Ling: 2530, 3830, 6110, 9000
Jarosek, Stephanie: 9010
Jarrín, Olga F.: 4500
Jarrott, Shannon E.: 1080, 2350, 3780, 3890, 5000
Jarvis, Megan: 6110
Jason, Kellyn: 9010
Jason, Kendra: 6180, 6750
Javeed, Ashir: 3590
Javier, Noelle Marie: 4250
Jayakody, Oshadi: 6780
Jayani, Reena V.: 6740
Jeamjitvibool, Thanakrit: 6411
Jeanblanc, Alexandra: 3115, 5140, 6090, 6820
Jean-Louis, Girardin: 6680
Jean-Pierre, Sarai N.: 9030
Jeffrey, Kullgren: 3495
Jenkins, Emerald: 4680
Jenkins, Hailey-Thomas: 6500
Jennings, Lee A.: 5172
Jennings, Stephen: 5050
Jen, Sarah: 2540, 6070, 6190
Jensen, Adelaide: 6290
Jensen, Christine J.: 6070
Jeong, Chung Hyeon: 4370, 5150
Jeong, Ha Na: 6320
Jeong, Mijin: 6070
Jeong, Sohyun: 9020
Jepson, Laura: 9020
Jessee, Lisa: 9030
Jessup, Nenette M.: 9020
Jeste, Dilip V.: 3760, 6200
Jester, Dylan J.: 4465, 6200
Jiang, Hantao: 9030
Jiang, Jingjing: 3200
Jiang, Kening: 2670, 3560, 4050
Jiang, Nisi: 9010
Jiang, Shumin: 6480
Jiang, Xin: 4040
Jiang, Yanping: 3860, 6200, 6290
Jiao, Yuqin: 3115
Jia, Yuane: 5130
Jicha, Gregory: 1380, 4790, 4870, 6060, 6720
Jimenez Zambrano, Joyce: 6720
Jimenez, Daniel E.: 6170
Jing, Bocheng: 5030
Jin, Yichen: 4200
Ji, Tong: 4683, 9020
Jiwani, Rozmin B.: 6390
Ji, Yuelyu: 6150
Jock, Janet: 9010
Johanessen, Aud: 3610
Johanning, Jason: 6740
Johansson, Linda: 9020
John, Karen: 1480

John, Oliver P.: 2620
John, Samantha: 6060
Johnson, Adrienne L.: 4682
Johnson, Alisha H.: 3483
Johnson, Brittany: 9030
Johnson, Daniel: 6350
Johnson, Elma B.: 3310
Johnson, Erin: 9020
Johnson, Erin P.: 3500
Johnson, F. Bradley: 6010
Johnson, Florence U.: 2420, 6310
Johnson, Fred: 9000
Johnson, Gerhardt: 6420
Johnson, Haley: 6510
Johnson, Ian: 4380
Johnson, Julene K.: 9000
Johnson, Justin: 1190
Johnson, Kim G.: 6450
Johnson, Kimson E.: 9010, 9020
Johnson, Mark: 5001
Johnson, Matilda: 3260
Johnson, Rachel: 6470, 9030
Johnson, Theodore M.: 9010
Johns, Shelley A.: 9000
Johnston, Daphne M.: 1380
Johnston, Jeanne: 9020
Johnston, Julia: 1380, 6060
Johnston, Maxwell: 2501, 6840
Johri, Mohini: 9030
Jolliff, Anna: 9010
Jones, Brian D.: 4160
Jones, Brittany: 4540
Jones, Chelsey: 9000
Jones, Clifford: 6100
Jones, Faith M.: 3460
Jones-Harden, Brenda J: 4225, 6180
Jones, Megan: 6450, 6510, 9000
Jones, Miranda: 2070
Jones, Patricia L.: 1440, 3080
Jones, Randy: 2070
Jones, Rebecca: 2540
Jones, Richard: 3510
Jones, Susan: 9030
Jones, Tessa: 2130, 6110
Jonsson-Funk, Michele L.: 4320
Joo, Heesoo: 6400
Joo, Susanna: 6390, 9030
Jopp, Daniela S.: 2440, 6200, 6380, 6480, 6580, 6790, 6830
Jor'dan, Azizah: 9010
Jordan, Kala v.: 9000
Jordan, Sarah R.: 4260
Jordan, Tamara: 3770
Joseph KJ, Vinod: 1060
Joseph, Carly A.: 4110
Joshi, Anupam: 6790
Joshi, Shantanu: 9000
Jox, Ralf J.: 2680, 3720, 6730
Joyce, Jillian L.: 6260
Joyce, Melinda: 9030
Jubanyik, Karen: 5100
Ju, Catherine H.: 6520
Judd, Suzanne: 1050
Judge, Katherine: 4010, 6130, 6310, 6410, 9000, 9020, 9030
Ju, Eunae: 2070
Juliano, Celina: 3810
Julian, Vallejo: 5001
June, Andrea: 1070, 1420, 3800
Jung, Hailey H.: 4540
Jung, Heeeun: 9020
Jung, Hye-Young: 2090
Jung, Jong Hyun: 6240
Jung, Karen: 2330
Jung, Woosub: 6420
Junqueira Vasques, Ana Carolina: 9020
Jurgens, David: 4630
Juris, Jill: 2350, 3270, 3780
Ju, Sehyun: 6240
Justice, Amy C.: 9010
Justice, Jamie N.: 2241, 2760, 3400, 4691, 9010
Justinas, Narbutas: 3590
Jutkowitz, Eric: 4580, 5130, 6230, 6360, 6540
Kühn, Simone: 1010, 4040

**K**

Kaasalainen, Sharon: 3555
Kabaya, Sonoko: 9000
Kabeto, Mohammed U.: 3040
Kabudula, Chodziwadziwa Whiteson: 3040
Kadey, Kylie: 6450
Kado, Deborah: 2290
Kaeberlein, Matt: 6840
Kahana, Eva: 2640, 4905, 6170, 6200, 6390, 6690
Kahana, Jeffrey S.: 6390
Kahn, Kathleen: 3040
Kahya, Melike: 3482
Kai, Ichiro: 3010
Kajiyama, Bruno: 6750
Kakarla, Prabhash: 9000, 9010
Kakinuma, Tomohiro: 2580
Kalaw, Karel: 9010
Kales, Helen: 2420, 2530
Kalesnikava, Viktoryia A.: 5060
Kalies, Christian H.: 6010
Kallianpur, Kalpana J.: 3580
Kallmi, Selmi: 3850
Kalvesmaki, Andrea: 4010
Kalyani, Rita R.: 1010
Kamal, Arif: 6390
Kamalyan, Lily: 2450
Kamkhoad, Donruedee: 6450
Kanagawa, Hotinpo Sky: 3481
Kanaya, Reiko: 6110, 6450, 6770, 9030
Kane, Alice E.: 1140
Kane, Kevin: 2290
Kane, Maureen A.: 6641
Kang, Bada: 6110, 6280
Kang, Chaehyeon: 6420
Kang, Ezer: 9010
Kang, Hyun: 6280
Kang, Jee eun: 6790, 9010
Kang, Karmeet Kaur: 3310
Kang, Miseon: 9030
Kang, Suk-Young: 6400
Kannan, Lakshmi: 4800, 6780
Kann, Michael R.: 6260
Kan, Winnie: 9010
Kao, Hsueh-Fen S.: 6140
Kao, Yu Hsiang: 4060
Kapahi, Pankaj: 6010, 6840
Kapasi, Alifiya: 2140
Kaplan, Daniel B.: 9030
Kaplan, Robert: 4330
Kapoor, Shaunik: 3670
Kappari, Sai Raj: 3260
Kappler, Caitlin B.: 5083
Karakaslar, Onur: 3001
Karakida, Maki: 6200
Karalija, Nina: 9010
Karam, Rita: 4555, 6130, 6670, 6710
Karasik, David: 3400
Karasik, Rona J.: 3030, 3420, 6000, 6220
Karbakhsh, Mojgan M.: 1210
Kåreholt, Ingemar S.: 3440, 6200, 6450, 6760
Karel, Michele J.: 6110
Karia, Francis: 6450
Karikari, Grace: 6350
Karim, Saleema: 6420
Kari, Nitya: 1320
Kariuki, Jacob K.: 9030
Karki, Dinesh: 6390
Karlamangla, Arun S.: 6440, 6580
Karlawish, Jason: 3680, 3930
Karmacharya, Isha: 2020, 6750, 9010, 9030
Karmonik, Christof: 3481
Karnati, Yashaswi: 6510
Karraker, Amelia W.: 2550
Karr, Justin E.: 6590
Karvar, Mehran: 6840
Karvonen-Gutierriez, Carrie: 2080, 6140, 6650, 9020
Kaskie, Brian P.: 2245, 3660
Kasler, Kamryn: 2340, 4360
Kasseye, Eyayu: 6330
Kassim, Adetola: 6740
Kastner, Keri: 2590
Kastner, Monika: 6380
Katagiri, Keiko: 6700
Katsev, Danielle: 1120
Katsumata, Asako: 3920, 4440, 6630
Kattari, Leo: 1400
Katz, Benjamin: 2580, 6290, 6440
Katzel, Leslie: 5050, 6220
Katz, Gabrielle R.: 6360
Katz, Jesse: 9000
Katz, Maya: 2720
Katz, Mindy J.: 6290
Katzorreck, Martin: 1020
Kaufman, Brystana G.: 3390
Kaufman, Jennifer e.: 3760
Kaufmann, Christopher N.: 2170, 4080, 4560, 4840
Kaup, Migette L.: 9030
Kaur, Harmanpreet: 6770
Kaushal, Navin: 3630, 6080, 6570, 6740
Kavalieratos, Dio: 4530
Kavanagh, Colleen: 6430
Kavasch, Barrie: 9010
Kavcic, Voyko: 6090, 6290
Kayali, Nahla: 3430
Kaye, Jeffrey: 1340, 2450, 4790, 6080, 6450, 9000
Kaye, Jesse T.: 4682
Kaye, Lenard: 3540, 6540, 9030
Kayser, Jay R.: 6580
Kazembe, Neo: 6240
Kazmer, Michelle M.: 4681
Kearney, Leanne: 6280
Kearns, William D.: 3540
Keatings, Margaret: 6270
Keefe, Bronwyn: 6130
Keel, Pamela K.: 6020
Keenan, Teresa A.: 3495
Keesee, Francesca: 6280
Kehler, Dustin S.: 9030
Keilman, Linda: 1400
Keith, NiCole R.: 6080

Kelekar, Uma: 9030
Kelfve, Susanne: 6200
Kellar, Garrett: 1280
Keller, Abiola: 6750
Keller, James: 4021
Keller, Krystyna R.: 6170
Keller, Michelle S.: 5083
Kelley, Amy S.: 4250, 5030, 5070
Kelley, Amy: 3080, 3280
Kelley, Casey J.: 9030
Kelley, Jessica A.: 4070
Kelley, Lynette: 6420
Kelley, Megan: 2340, 6130
Kelly-Adams, Deirdre: 6230, 6690
KELLY, AMBER L.: 5060
Kelly, Brenna C.: 4530
Kelly, Christopher M.: 6700
Kemp, Candace L.: 5173, 6760
Kendrick, Alexis: 6740
Kennedy, David: 6690
Kennedy, Katherine A.: 3950, 5081, 9030
Kennedy, Meaghan A.: 1040
Kennedy, Richard E.: 6340
Kennedy, Teri: 6120
Kenny, Brooks: 6140
Kerdic, Richard: 2090
Kern, Kathy L.: 9000
Kerns, Lucy: 1280
Kero, Katherine: 6090, 6290
Kerr, Margaret: 4240
Kesler, Shelli R.: 9020
Kessler, Jamie: 3040
Kew, Chung Lin: 2250, 6310, 6710, 9030
Ke, Weiming: 5173
Key, Kent: 6180
Kezios, Katrina: 9010
Khalil, Hussein M.: 6510
Khamzina, Madina: 2050, 6390
Khan, Amina: 9020
Khan, Amna: 3480
Khan, Anam M.: 3690
Khan, Arooj: 4080
Khan, Mushira: 6180, 9010
Khan, Nibraas: 5090
Khan, Shehroz: 6640, 9000
Khavjou, Olga A.: 3640
Khemai, Chandni: 6270
Khumrungsee, Montri: 2610
Khyzr, Maryam: 5001
Kiasat, Shadi: 6450
Kibe, Lucy: 9020
Kidana, Kiwami: 6240
Kiecolt-Glaser, Janice K.: 3150, 4470
Kiefer, Jamie: 9020
Kiekhofer, Rachel: 6270
Kiel, Douglas: 3400
Kiely, Kim: 4022
Kietzman, Kathryn G.: 3770
Killian, Tim S.: 6200, 9000
Kilpela, Lisa S.: 6020
Kim, Hyunji: 3190
Kimball, Patricia F.: 4090
Kim, Boeun: 2510
Kim, Bohyun: 9020
Kim, Bon: 3220, 6670
Kim, BoRin: 4370, 5150, 6800, 9000, 9010, 9020
Kim, Catherine S.: 3140
Kim, Chang Oh: 6320
Kim, Christina: 4240
Kim, Dae H.: 5084, 6110, 6641, 9000, 9020
Kim, DaEun: 9010
Kim, Dahye: 4170
Kim, Eun-Kyeong: 2450
Kim, Eunkyo: 6270
Kim, Eunkyung: 6400
Kim, Eunyoung: 6280
Kim, Giyeon: 6240, 6340, 6820
Kim, Gwang Suk: 6320
Kim, Ha-Neul: 1170, 4023, 4270, 6060, 9000
Kim, Harper S.: 2501
Kim, Heejung: 6400, 6420, 9030
Kim, Hongjik: 6240
Kim, Hyeon Jung: 6170, 6290
Kim, Hyoun K.: 6390, 9030
Kim, Hyunjee: 5081
Kim, Hyun-Jun: 4540
Kim, Jeehoon: 6400
Kim, Jeungkun: 6400
Kim, Jinhyun: 6810
Kim, Jiwon: 3020
Kim, Joo Ri: 6320
Kim, Joohyun: 2440
Kim, Jung Ki: 2080, 6710
Kim, Junhyoung: 3220, 6170, 6780, 9020
Kim-Knauss, Yaeji: 2480
Kim, Kyeongmo: 6120, 6610
Kim, Kyoungmi: 1470
Kim, Kyung Moo: 9020
Kim, Kyungmin: 1320, 2040, 2160, 2440, 3220, 4170, 4460, 6480, 6520, 6670, 6750, 6830
Kim, Laurie: 6420
Kim, Mackenzi: 9020
Kim, Meeryoung: 6670, 6810
Kim, Miji: 6450, 6510, 6690, 6740, 6780, 9020
Kim, Min Jung: 6080, 6300, 9010
Kim, Minji: 6320
Kim, Miyong T.: 6810
Kim, Narae: 3200
Kim, Patricia: 2300
Kim, Patricia S.: 6530
Kim, Seiyoun: 2090
Kim, Seo Yun: 9030
Kim, Seon: 2700, 6610
Kim, Seoyoun: 3160, 4660
Kim, Seulgi E.: 4683
Kim, Sohyun: 4870
Kim, Sooyoung: 5090
Kim, Steffi M.: 1380, 3790, 4710
Kim, Su Yeong: 4730, 6180
Kim, Sue: 6320
Kim, Sung Kyeom: 9010
Kim, Yeoju: 6240
Kim, Yesol: 6400
Kim, Yi Hyun: 6420
Kim, Yijung K.: 2440, 3730, 6480, 6830
Kim, Yongseop: 6780
Kim, Yoon-Myung: 6390
Kim, Youn Kyoung (Lily): 6500
Kim, Yulri: 9010
Kincaid, Reilly: 6520
Kindratt, Tiffany B.: 6310
King, Barbara: 6641
King, Ben: 3540
King, Christina: 3241
King, Gregory: 9010
King, Jennifer: 6430
King, Sharla: 6390
King, Sina J.: 4620
Kinkade, Emily E.: 6230, 6640, 9020
Kinney, Jennifer M.: 2440
Kinosian, Bruce: 1330
Kinzig, Elizabeth: 3530
Kipnis, Jonathan: 4461
Kirch, Matthias: 3495
Kirkegaard, Allison: 6690
Kirk, Jennifer M.: 6740
Kirkland, James L.: 2760
Kirk, Laura: 9000
Kishimoto, Kyoko: 3420
Kitamura, Akihiko: 6710
Kitase, Yukiko: 5001
Kitner-Triolo, Melissa: 9020
Kitsiou, Spyros: 6780
Kittle, Krystal R.: 1320, 2390
Kivi, Marie: 4620
Kiyoshi-Teo, Hiroko: 9030
Klaiber, Patrick: 2430
Klassen-Ross, Tammy: 2210
Klee, Matthias: 6150
Kleineidam, Luca: 2140
Klepacz, Laura M.: 2470, 9020
Kleszynski, Keith: 3610, 5172
Kline, Danielle: 4650
Klinedinst, Jennifer: 6641
Klinger, Christopher: 6230, 6480, 6690
Klopack, Eric T.: 3440, 4560, 6010
Knebl, Janice A.: 1110, 1250
Knebusch, Clare: 6160
Knepple Carney, Amy: 9000
Knight, Jamie: 2140, 6290
Knight, Rebekah: 3190
Knimbeng, manka: 3310
Knopman, David S.: 3560, 6780
Knopp-Sihota, Jennifer A.: 6270
Knox, Janice: 4681
Knox, Katie: 2520
Knurek, Sean: 6180
Kobayashi, Lindsay C.: 1050, 2270, 3040, 3495, 4110, 9010
Kobayashi, Masaki: 6000
Kodali, Maneetha: 6180
Koehler, Kerstin: 3090
Koehly, Laura M.: 1480, 3240, 6170
Koerner, Susan S.: 6240
Ko, Eunjung OS: 5090
Koeuth, Sokha: 3310
Koffer, Rachel E.: 2220, 3070
Ko, Fred: 1350, 6320
Kohanski, Ronald A.: 3080
Koh, Eun: 6480
Koh, Jemima: 3820, 6330
Kohn, Jordan N.: 6200
Koi, Janice Jue Xin: 6411
Koiler, Reza: 4021
Koirala, Binu: 6140
Koizumi, Naoru: 3200
Kok, Almar A. L.: 9020
Kolanowski, Ann: 2160, 3310, 3720, 9030
Kolodisner, Jude G.: 2250
Kolodziejczak, Karolina: 3150
Kolodziejczyk, Ida: 6510
Kolpakov Nikitin, Stanislav: 6411
Koltermann, Ken: 6420
Koltz, Daniel J.: 6760
Koltz, Rebecca L.: 6760

Komalasari, Renata: 6110
Komatsu, Ayane: 6210, 6250, 6760
Kong, Dexia: 3500, 9030
Kong, Jooyoung: 4730, 6520
Kong, Lan: 3520
Kong, Taylor: 9030
Konomos, Michael: 2640, 3550
Konrad, Thomas R.: 9010
Kontra, Joseph: 6100
Koo, Hyun Jung: 2420, 5070
Kooiman, Nia: 3520
Koon, Lyndsie M.: 4160
Kootar, Scherazad: 9000
Kopera-Frye, Karen: 1480, 6100
Kops, William: 9030
Koren, Chaya: 1300
Koren, Yael: 4640
Koreshi, Shanika Y.: 3900
Kornadt, Anna E.: 2750, 3490
Kossowska-Kuhn, Dorota: 9020
Kostyniuk, Lidia P.: 3000, 6470
Koszalinski, Rebecca: 1060
Kotecho, Messay G.: 6330, 6630
Kotwal, Ashwin A.: 2550, 2690
Koufacos, Nicholas: 6220
Koumoutzis, Athena: 2440, 3180, 4010
Kovaleva, Mariya: 5160
Kovtun, Mikhail: 6110
Kowalchik, Kalei H.: 3520
Kozlov, Elissa: 1310, 6390
Kraemer, Sandra: 6170, 6580, 6790
Krakauer, John W.: 3750
Kramer, Jenna: 4555, 6130, 6670, 6710
Kramer, Philip A.: 9010
Kraschnewski, Jennifer L.: 3520
Krasnansky, Katie: 4680
Krasniewski, Linda: 1470
Krassikova, Alexandra: 6270
Kratzer, André: 4300
Kraus, William E.: 2241
Kravatz, Nigel L.: 9020
Kravchenko, Julia: 1000, 3720, 6110
Kremen, William S.: 6050, 6490
Krendl, Anne C.: 3570, 6290, 6810
Kresevic, Denise M.: 9030
Kriebernegg, Ulla: 3585
Krishnan, Girish: 9020
Kritchevsky, Stephen B.: 2241, 2300, 2530, 3340, 3400, 4120, 4600, 6641, 9000, 9010
Kroeber, Maria M.: 6000, 6220
Krok-Schoen, Jessica L.: 2200, 3550
Krook, Melissa: 6790
Kropp, Denise: 3530
Krupp, Anna: 9020
Kryscio, Richard: 4870
Kubala, Andrew G.: 4080
Kuball, Tim: 6250
Kube, Erin R.: 2030
Kubzansky, Laura D.: 4690
Kuchel, George A.: 3001, 4410
Kuipers, Allison L.: 4810, 6450
Kulbok, Pamela: 1030
Kulkarni, Rajendra: 3200
Kulkarni, Snehal S.: 9030
Kullgren, Jeffrey: 3495
Kulminski, Alexander: 6010, 6411, 9030
Kumar, Amit: 4780
Kumar, Premranjan: 2241, 9010
Kumar, Priyanka: 3560
Kumar, Raj G.: 5030
Kumar, Sharma: 4190
Kumar, Sidharth: 6390
Kunkel, Miranda C.: 1530, 2340, 4360
Kunkel, Suzanne: 2010, 2020
Kunkle, Rachel: 6280
Kunz Lomelin, Alan Adolfo: 6630
Kunzmann, Ute: 1020
Kuo, Tony: 6060, 6220, 6280
Kuo, TsuAnn: 4440, 4450
Kuo, Yong-Fang: 5100, 9000, 9010
Kurazume, Ryo: 6000
Kurth, Maria: 5140, 6210, 9020
Kushnir Groman, Tamara: 3390
Kusmaul, Nancy: 4510, 4830, 6720
Kusters, Cynthia D.: 4560
Kutcher, Katie: 4520
Kuwayama, Sayaka: 9000
Kuy, Kakada: 6200
Kuzmik, Ashley: 2230, 4760
Kwak, Jung: 6270
Kwan, Joseph Shiu Kwong: 4684, 5084
Kwan, Rick Yiu Cho: 3840
Kwekkeboom, Kristine: 6280
Kwok, Wai-wai: 9020
Kwon, Eunsun: 9010, 9030
Kwon, Jenny: 9000
Kwon, Simona C.: 1480
Kwon, Soonhyung: 4190
Kyriakos, Markides: 6210
Löckenhoff, Corinna: 6210, 6690
López-Anuarbe, Mónika: 6310
López-Ortega, Mariana: 3020

L

Lach, Helen W.: 6100
Lachman, Margie E.: 2140, 2430, 6440, 9030
Lachs, Mark: 4090, 6480, 9000
LaCroix, Andrea Z.: 4560, 6020, 9030
LaCroix-Fralish, Michael: 9010
Ladda, Shawn: 3680
Ladd, Ilene G.: 4150
Laditka, James N.: 2050
Laditka, Sarah B.: 2050
LaFave, Sarah: 9010
Laffer, Alex: 6570
Lafronza, Vincent: 6120
Lai, Daniel WL: 4090
Laine, Abby J.: 3850
Lai, Patrick Ho Lam: 9030
Lakadamyali, Melike: 1200
Lalani, Nasreen: 9030
Lalji, Kahir: 2600
Lamba, Shiv: 6420
Lambert, Dawn: 1310
Lamerato, Lois: 9020
Lam, Justin W.: 9000
LaMonte, Michael J.: 9030
Lampe, Nik M.: 2390, 4681, 9030
Lampkin, Cheryl: 3495
Lampraki, Charikleia: 6380, 6580, 6790
Landes, Scott: 2660
Landi, Francesco: 3830
Landman, Bennett A.: 4200
Landreth, Gary: 6220
Lane, Nancy E.: 6200
Lane, Sandi J.: 4820, 6090
Langa, Kenneth M.: 1050, 2270, 3000, 3130, 4690, 6150
Langan, Erica: 3390
Lange-Maia, Brittney: 6650
Lang, Frieder R.: 2480, 2750
Langhi Prata, Larissa: 6840
Lang, Samantha: 9010
Lanham, Holly: 4210
Lanigan, Kerry: 4010, 6130
Lanternier, Paula: 6090
Lapane, Kate: 2640, 6770, 9010, 9020
Lapid, Maria I.: 2160
LaPierre, Tracey: 2720
Laporte-Uribe, Franziska: 3610
Laraia, Barbara: 3070, 3440
Larkey, Linda K.: 9020
Larkina, Marina: 2220, 6050
Larned, Joshua: 3483, 4021, 6740, 9020, 9030
Laryionava, Katsiaryna: 5110
Lassell, Rebecca KF: 2130, 6110
Latham-Mintus, Kenzie: 5080
Lathrop, Laura: 9000
Latimer, Abigail: 3930
Latimer, Nia: 3140
Latkin, Carl: 2690, 6800
Latshaw, Emily: 5090
Lauber, Mckenzie: 6280
Lau, Bobo Hi Po: 4022, 4684, 5084
Lauderdale, diane: 1230
Lau, Jackson: 6641
Laurant, Miranda: 4720
Lavalley, Ryan: 4360
LaValley, Susan A.: 6430
Lavender, Kathryn: 6830
Lavery, Angela: 3610
Lawhorne, Larry: 1060
Lawlor, Brian: 2690
Lawrence, Carly W.: 9030
Law, Royal: 4100
Lawson, Kimberly: 4650
Lawson, Olivia: 6270
Layman, Shannon: 4510
Leach, Carrie: 4590, 6400
Leach, Heather J.: 2280, 3930
Leahy, Suzanne: 6220
Leal, Isabel: 6180
Leaphart, Desirae: 4240
Leavey, Gerry: 2690
LeBlanc, Brian: 4270
LeBlanc, David J.: 3000
LeBrasseur, Nathan: 2241, 3241
Lecchino, Catia: 6570

Lech, Sonia: 6110
Ledbetter, Leila: 9020
Le, Dung D.: 6300
Lee, Alexandra K.: 6110
Lee, Amanda: 3050
Lee, Anna K.: 2380
Lee, Bih-O: 6140
Lee, Cathy C.: 4260
Lee, Chae Man: 3100, 6090, 6510
Lee, Changhan D.: 4001
Lee, Changmin: 6390
Lee, Chiyoung: 9030
Lee, Chung Eun: 9020
Lee, Daehyun: 6450, 6510, 6740
Leedahl, Skye N.: 2350, 3780, 6400
Lee, Deborah: 3450
Lee, Dylan: 6210, 9020
Lee, Edward B.: 6010
Lee, Ellen E.: 9000
Lee, Eun Jeong: 6480
Lee, Gina: 4140, 6810
Lee, Haena: 6240
Lee, Hannah: 5070, 6310
Lee, Harold: 4690
Lee, Hee Y.: 3220, 6100, 6180
Lee, Hye Soo: 6210, 9020
Lee, Hyo Jung: 6620
Lee, Hyunmin: 6500
Lee, Jane S.: 6690
Lee, Janet Lok Chun: 3840
Lee, Jeannie K.: 4190
Lee, Jennifer: 3050
Lee, Jeong Eun: 2200
Lee, Jessica: 6770
Lee, Ji Hyun: 1240
Lee, Ji Yeon: 6320, 6770
Lee, Jihye: 9030
Lee, Jinkook: 2270
Lee, JuHee: 6280
Lee, JuHyeong: 6240
Lee, Jung-Ah: 2070
Lee, Jungjoo: 6170
Lee, Kathy: 6530
Lee, Kelsey: 6010
Lee, Kyung Hee: 6320, 6390, 6770, 9010
Lee, Kyungmi: 6390
Lee, Lewina O.: 2620
Lee, Lewis H.: 3220
Leeman, Jennifer: 3482
Lee, Megan: 9020
Lee, Mi-Jung: 6780
Lee, Minhong: 6120
Lee, Mo-Yee: 4370
Lee, Rain: 6420
Lees Haggerty, Kristin: 4890
Lee, Sang E.: 6160
Lee, Sang Hwa: 9000
Lee, Sanghee R.: 6380
Lee, Sei: 6110
Lee, Seoyeon: 6750
Lee, Shinduk: 6310, 9030
Lee, Soo-Jeong: 2710
Lee, Soomi: 1100, 2620, 4080, 4560, 6200, 6600, 6710
Lee, Sophia: 3260
Lee, Sun Ah: 5060
Lee, Sunghee: 6420
Lee, Sunghoon I.: 9030
Lee, Susan: 4160
Lee, Trevor: 1330
Lee, Yeonjung: 4022, 4250, 4750
Lee, Yeonjung Jane: 3220, 6180
Lee, Yeseul: 9030
Lee, Yookyong: 3290
Lee, Yooumi: 3220
Lee, Youjung: 3740
Lee, Youran: 9020
Leff, Bruce A.: 1160, 5030
Legault, Véronique: 6140
Leger, Kate A.: 2430, 3730
Leggett, Amanda N.: 2420, 3481, 5070, 6310
Lehman, Chloe: 9000
Lehning, Amanda: 4510
Leipzig, Rosanne: 4250
Leis, Aleda: 6650, 9020
Leiser, Scott: 6010
Leist, Anja K.: 6150
Leist, Christine P.: 6350
Leithaus, Merel: 2090
Le, Joie: 3260
Lekhak, Nirmala: 4905, 6170, 6200
Lemaster, Philip: 6490
Lemieux, Mackenzie: 4461
Lenart-Bugla, Marta: 5170
Lenchik, Leon A.: 9030
Lendon, Jessica P.: 6100, 6540
Leng, Iris: 2241
Lenoir, Kristin M.: 4150
Lensing, Shelly Y.: 6360
Lenze, Eric: 3850
Lenzen, Natalie: 4050
Le, Ocean: 6530
Leonard, Natalie: 2420
Leoutsakos, Jeannie-Marie: 4050
Lepore, Michael J.: 6110, 9000
Lera, Lydia: 6320
Lerner, Alan: 6160
LeRoy, Angie: 9010
Lesandrini, Jason: 6760
Leshchyk, Anastasia: 2501, 4810, 6010
Leslie, Kathleen: 6640
Lessem, Rachel N.: 3390, 4270, 4590
L'Etoile, Noelle: 6411
Letts, Elyse: 6560, 6680
Leung, Dara Kiu Yi: 9020
Leung, Kevin: 6100
Levasseur, Melanie: 6140
Leveille, Suzanne: 1280, 4640, 6200, 9010
Levenson, Robert W.: 4681, 4850, 9000
Levi, Jonathan: 9010
Levine, David: 3660
Levine, Hannah: 1550
Levine, Morgan: 1140
Levin, Michael: 6410
Levkoff, Sue E.: 4700
Levy, AnneMarie: 2460
Levy, Becca: 3890, 5110
Levy, Joseph F.: 2580
Levy, Laura: 9000
Levy, Shellie-Anne: 3251
Lewin, Peter A.: 5020
Lewinson, Terri: 2570, 4380
Lewis, Jordan P.: 2310, 3790, 4010, 4710
Lewis, Nathan A.: 2140, 6290, 6580
Lewis, Stuart: 4090
Lewis, Tene: 2080
Leykum, Luci K.: 1330, 4010
L'Heureux, Tanya: 1420
Li, Angela: 6120
Liang, Jiaming: 1500, 2420
Liang, Peichao: 9030
Liang, Xiaohui: 4640
Lian, James: 4022
Liao, Hsiao-Wen: 9010
Liao, Xu: 9030
Liao, Yo-Jen: 2530, 6110, 9000
Libon, David J.: 6411
Li, Chengdong: 4190
Li, Chien-Ching: 6430, 9010
Li, Chin-Shang: 6110, 6280, 6510
Li, Choi Ha: 1500
Lichtenberg, Peter A.: 3251, 3800, 4090, 4230, 4400, 4870, 6290, 6480, 6580, 9030
Lichtenstein, Maya L.: 4150
Li, Cun: 5001, 9010
Liddy, Clare: 1510
Lieberman, Lauren: 2530
Liebman, Michael: 4360
Liebzeit, Daniel J.: 9020
Liechty, Toni: 3270, 6170, 6710
Li, Fanfan: 1130, 6450
Lighthall, Nichole: 2290, 3840
Light, Michael A.: 4380
Lightner, Joey: 9010
Li, Grace: 6210
Li, Guohua: 5171
Ligus, Kaleigh: 9000
Li, Hui-Ling: 6530
Li, Jie (Jenni): 3230
Li, Jing: 5030
Li, Jingyi: 1150
Li, Juan: 1130, 6140, 6450
LI, Junxin: 3330, 4080, 4640, 6600
Li, Kaigang: 5110
Li, Ke: 4240, 6680
Likos, Kelly: 2390
Li, Lun: 4250, 4750, 6530
Li, Lydia: 3010
Lima Passos, Valeria: 5100, 6560
Lim, Aaron: 6580
Lim, Emily: 6810, 9020
Li, Mengchi: 4080, 4640
Li, Meng-Hao: 3200
Li, Mengting: 3860
Li, Mengze: 4810, 6010
Li, Minghui: 3230
Li, Mingyang: 3540
Li, Mirandy: 4060
Lim, Jue Tao: 3390
Lim, Julian: 9030
Lim, Junyub: 6820
Lim, Keith: 9030
Lim, Ki: 4160
Lim, Kia Yee: 3820
Lim, Michelle H.: 6790
Lim, Miranda: 2450
Lim, Nicole: 3620
Lim-Soh, Jeremy: 4170, 6710
Lim, Swee Ho: 3390
Lin Gomez, Scarlett: 3690
Lin, Carol: 5083
Lincoln, Karen D.: 3420, 3890
Lindauer, Allison: 4790, 6080
Lindberg, Brian W.: 1110, 2740, 3120, 3300, 3495, 3960
Lindberg, John E.: 2710
Linden, Anna: 6310, 9000, 9010
Lindenberger, Ulman: 1010, 3070, 4040

Linden-Carmichael, Ashley: 6260
Lind, Kimberly A.: 6430
Lind, Majse: 6300
Lindmark, Ulrika: 9020
Lindquist, Lee: 1030
Lindsey, Ronald W.: 1120
Lin, Frances: 4241
Lin, Frank R.: 2670, 3560, 4050, 4680
Lin, Haiqun F.: 4500
Lin, I-Fen: 1300, 1450, 4070
Lin, Jielu: 1480, 6170
Lin, Joshua K: 6641
Link, Christopher D.: 9010
Links, Paul S.: 4630
Lin, Pay-Shin: 9010
Lin, Rachel R.: 6140
Lin, Ruei-Lung: 6411
Lin-Schweitzer, Anna: 9020
Lin, Shayne S.: 9020
Lin, Shih-Yin: 2130, 3060, 6110
Lin, Sunny: 6540
Lin, Tianze: 4040
Lin, Xin Yao: 9030
Lin, Yan: 3320, 9020
Lin, Yen: 3120
Lin, Yi Chun: 5090
Lin, Yuxin: 6130
Lin, Zhixuan: 6250
Lin, Zhiyong: 4010, 4460, 6750
Lin, Zhuoer: 5010, 6450
Liotta, Madeleine M.: 4240
Liou, Chih-ling: 9010, 9020
Liou, Shyhnan: 2480
Lipnicki, Darren M.: 4340
Lipsitz, Lewis A.: 3482, 3510, 3600, 9030
Lipton, Richard B.: 6290
Li, Qinghua: 4350
Li, Qiwei: 4640
Li, Sha: 4682
Li, Shijie: 9020
Li, Shiyu: 4190
Lithgow, Gordon: 9010
Little, Kristina: 6500
Little, Milta O.: 6450
Litzelman, Kristin: 4240, 4510, 6280
Liu, Chang: 1400
Liu, Chiung-ju: 6740, 6770
Liu, Chun: 2241
Liu, Chunyu: 9020
Liu, Darren: 1070, 6090
Liu, Difei: 5171
Liu, Fangyu: 4120, 4330
Liu, Haiyong: 3100
Liu, Hao (Howe): 6780
Liu, Hui: 1230, 2400
Liu, Huiying: 6530
Liu, Jason Jiashuan: 3483
Liu, Jinyu: 3010, 4240, 6230, 6720
Liu, Kristy: 6780
Liu, Li-Chuan: 6000
Liu, Lili: 6390, 6560, 6680
Liu, Liu: 3670
Liu, Michelle: 9010
Liu, Mingxin: 6140
Liu, Pan: 4290, 4683, 9020
Liu, Pi Ju: 6630
Liu, Pi-Ju: 4900
Liu, Rui: 3290
Liu, Ruotong: 6750, 9030
Liu, Sizhe: 1080
Liu, Tianyin: 3481, 9020
Liu, Wei-Cheng: 6700
Liu, Wen: 2520, 2530, 3450, 3830, 4870, 9000
Liu, Xiaoji: 9030
Liu, Yongmei: 3400
Liu, Xinran: 6130
Liu, Yin: 4560
Liu, Yinghao: 9010
Liu, Yingxu: 9000, 9010
Liu, yongmei: 3400
Liu, Yujun: 6750
Liu, Yunda: 9030
Liu, Yuqi: 9030
Liu, Zhijian: 1130, 6140
Liu, Zhong-xu: 6290
Liu, Ziming: 9000
Liu, Zuyun: 6411
Livermore, Michelle M.: 9020
Li, Wenjun: 2290
Li, Xiaoli: 3540, 6540
Li, Xiaoqian: 6411
Li, Ying: 9020
Li, Yixia: 2510
Li, Yue: 4580, 6140
Li, Yuelin: 9010
Li, Yun: 4290, 4683, 9020
Li, Zhuoying: 6641
Lkhagvasuren, Khorolsuren: 4684
Llaneza, Danielle H.: 4691, 6390
Lobchuk, Michelle M.: 1420
Lockery, Jessica: 5083
Lock, Sarah L.: 1430
Loeb, Susan: 6700
Loerzel, Victoria: 2290, 3840
Loewenstein, David: 4090
Loganathar, Priya: 9000
Logan, Paige E.: 3570
Logsdon, Rebecca: 3000
Logue Cook, Rachel N.: 9000
Logue, Melissa: 6740
Loh, Feng-Hua: 9010
Lohman, Kurt: 3400
Lohman, Matthew: 6320
Loika, Yury: 6010
Loiko, Elena: 6010
Lokanathan, Tharshini: 2290, 3840
Lomeli, Sindy: 3390, 6120
Longo, Valter: 2180
Lo, On-Yee: 3482
Lopes Deliveira, Thais: 3590
Lopes, Maria: 2620
Lopez Mendez, Mauricio: 6360
Lopez Paz, Cinthie: 6060
Lopez, Acacia: 3740, 6520, 6580
Lopez, German: 9030
Lopez, Maria J.: 4500
Lopez, Noelle: 3190
Lord, Justin: 3660, 4820
Lorenz, Karl: 6430, 6530
Lorenzo, Erica C.: 9010
Lorenz, Rebecca A.: 6100, 6510
Lorraine, Mion C.: 5090
Losinski, Genna: 6570
Lou, Vivian Wei Qun: 3840
Louras, Peter: 6010
Lou, Vivian Wei Qun: 3840, 4090, 6530
Louwagie, Katie W.: 4220, 6070
Lou, Yifan: 3010, 4240, 6720
Love, Catherine: 2410, 3770
Lowers, Jane: 4530
Lowther, Johnna: 1420
Lox, Dennis M.: 6641
Lucas, Kaitlyn: 6410
Lucchini, Roberto: 1350
Lu, Chi-pang: 4450
Luchsinger, Jose A.: 2160
Luciano, Michelle: 6450
Lücke, Anna Jori: 1020
Łuc, Mateusz: 5170
Ludlow, Jared A.: 6510
Luezas-Shamakian, Lorella: 4100
Luft, Benjamin: 4700
Lui, Kitty K.: 9000
Lui, Li-Yung: 4120
Lujan, Irene: 3460
Lu, Kaiwei: 6320, 6780
Lu, Kevin: 3230
Luk, James Ka Hay: 4022, 4684, 5084
Lukkahatai, Nada: 3330, 4080
Lum, Hillary: 4280, 6210, 6420, 9000
Lum, Terry Y.: 3481, 9020, 9030
Lundgren, Dan: 6760
Lund, Jennifer L: 4320
Lunetta, Kathryn L.: 3400, 6450, 6740
Lun, Janetta: 1220
Luo, Hao: 6110
Luo, Huabin: 3870
Luo, Jiangtao: 3540
Luo, Jing: 2620, 4620
Luo, Liying: 6610
Luo, Minxia: 1020, 2450
Luong, Gloria: 2280, 2470
Luo, Yan: 6100, 6180
Luo, Ye: 2080
Lu, Peiyi: 3500, 9010, 9030
Lu, Shiyu: 9030
Lustgarten, Michael: 4810
Lu, Yi: 6210
Lu, Yvonne Y.: 9020
Lu, Zhaohui: 6100
Lydon, Elizabeth A.: 6290, 6300, 6390
Lyketsos, Constantine G.: 4050
Lyles, Courtney: 6270
Ly, Monica: 9000
Lynch, David H.: 2080, 4640, 6780
Lynch, Delanie: 9000
Lynch, Kristine E.: 6320
Lynch, Morgan E.: 6290
Lynott, Jana: 3100
Lyons, Amy: 6770
Lyons, Karen S.: 3240, 6520
Ly, Timothy K.: 9020
Lytle, Megan N.: 9020
Márquez, Freddie: 9000
Mäki-Petäjä-Leinonen, Anna: 2460

**M**

Macarenko, Yulia: 9030
MacCarthy, Daniel: 6320
MacCormack, Jennifer K.: 4570
Macdonald, Birthe: 1020
MacDonald, Catherine: 9000
MacDonald, Stuart: 6590
MacGregor, Tara: 6270
Ma, Chenjuan: 3950, 4370
Mack, Deborah: 6770, 9010
Mackenstadt, Darby: 6790
Mackenzie, Corey: 6790
Mackiewicz, Marissa K.: 6000, 6320
Mack, Wendy: 9020

Mack, William: 9020
Maclagan, Laura: 9000
MacLean, Alyssa: 6440
MacLean, Maria C.: 6250, 6640
MacNeil, Amanda M.: 9000
MacNeil, Andie: 4090, 4225, 6200, 6480
Madarasmi, Saira: 4190
Madden, Kenneth M.: 3150, 6580
Madera, Sonia: 6420
Madero, Erica: 6510
Madison, Annelise A.: 4470
Madison, Barqueisha: 6760
Madrid Fuentes, Diana A.: 9000
Maeda, Harumi: 4470
Maercker, Andreas: 5100
Ma, Flora: 2030, 3420
Magana, Maribel: 3750
Magee, Catherine: 6280
Mage, Susanna M.: 4510, 6310
Magnuson, Justin: 1110
Mahmood, Atiya N.: 6500
Mahmoudi, Elham: 3130, 4040, 6060
Mahon, Elizabeth D.: 6440
Mahoney, Martin C.: 6430
Maierhofer, Roberta: 4670
Mailhot, Tanya: 3720
Main, Eve: 9030
Mair, Christine A.: 4690, 4860
Majid, Marjan: 4050
Makaroun, Lena K.: 4900, 9000
Makhnevich, Alex: 4700
Mak, Wingyun: 6100, 9010
Malani, Anurag N.: 9020
Malani, Preeti: 3495
Malani, Revati N.: 9010
Malatyali, Ayse: 4500
Malhotra, Atul: 9000
Malhotra, Rahul: 2150, 3160, 4170, 6710
Ma, Lina: 4290, 4683, 9020
Maliski, Sally L.: 6120
Mall, Neshay: 9030
Malone, Michael: 6280
Malone, Susan K.: 1130
Ma, Luyan: 6170
Maly, Monica: 3760
Ma, Melissa: 9000
Mamo, Sara K.: 6270
Manalel, Jasmine: 1290, 3760
Manchella, Mohit K.: 3570, 4860, 6820
Mandelblatt, Jeanne S.: 1100, 3400
Mandile, Noah: 9030
Manella, Kathleen J.: 2060
Manggaard, Jennifer: 6110
Manheim, Chelsea: 2660, 2720
Manietta, Christina: 3090
Manini, Todd M.: 2170, 4840, 6320, 6510
Manisha, Sengupta: 6100, 6540
Manja, Veena: 6530
Manjourides, Justin: 5084
Manly, Jennifer: 4470
Mann, Frank D.: 3510, 6440
Manning, Carol: 1030, 9010
Manning, Kenneth: 5050
Manning, Lydia k.: 6580
Mann, Jim: 4860, 9000, 9010
Mann-Krzisnik, Dylan: 3630
Manoharan, Sneha: 6390
Manor, Brad: 3482, 3510, 9010, 9030
Manrique Espinoza, Betty Soledad: 4290
Mansion, Natalie: 3670
Mansoor, Marrium: 6440
Mao, Hui-Fen: 6350
Mao, Weiyu: 1540, 3870
Mao, Ziling: 3830
Marano, Thomas: 6100
Marashi, Sheida: 2460
Marasinghe, Himasara: 6150
Maras, Jessica: 2430
Marcano Ocasio, Claudia Beatriz: 3483
Marcano, Claudia: 4021
Marceau, Kristine: 6790
Marchal, Noah: 4021
Marches, Radu: 3001
Marcinek, David: 9010
Marconi, Vincent C.: 9010
Marcus, Michael S.: 4880
Mardini, Mamoun T.: 4840, 6320, 6510
Margalef, Carole: 9010
Margheim, Lynn: 5171
Margolis, Rachel: 4070, 6300, 6310, 9020
Margrett, Jennifer: 9020
Mariani, Diane: 2560
Mariani, Jean: 9010
Marilyn, Rantz: 1460, 4021
Marinez, Julia: 3010
Marini, Christina M.: 2660, 3480, 6170, 6210, 9030
Marino, Francesca: 4330, 4600
Marino, Victoria R.: 2620
Markert, Dana: 1170
Markides, Kyriakos S.: 3240, 6300
Markids, Kyriakos: 3020
Markkanen, Pia K.: 2710
Markus, Marcello: 1010
Markwardt, Sheila: 6740
Marmo, Suzanne: 3290
Marnfeldt, Kelly A.: 3010, 9000
Marottoli, Richard: 5100, 5172
Marquez, Carlos: 6320
Marquez, David X.: 4800, 6570
Marquez, Eladio: 3001
Marroig, Alejandra: 9020
Marrone, Nicole: 3560
Marroquin-Serrano, Madeleine: 6000
Marrs, Sarah: 2260, 9000
Marsack-Topolewski, Christina: 3860, 9010
Marshall, Ami: 5172
Marshall, Gillian L.: 6180
Marshall, Lynn M.: 6320
Marshall, Shawn: 6170
Marshall, Victoria: 6020
Marsh, Courtney A.: 6120
Martens, PhD, Nancy L.: 6420
Martí Haidar, Yamile M.: 2700
Martin, Damali N.: 3430
Martin, Diane J.: 1370, 3140, 9000
Martin, Dominique E.: 9010
Martin, Emily T.: 9020
Martinez Lenis, Vanessa: 6250
Martinez-Amezcua, Pablo: 4050
Martinez, Beatrice: 6770
Martinez, Iveris L.: 5084
Martinez, Sujey: 3750
Martin, Mike: 2450, 6380
Martino, Alexa: 1240
Martin, Peter: 3580, 4022, 4140, 4684, 5084, 6810
Martin, Rolf J.: 9010
Martins-Klein, Bruna: 6210, 6411
Martire, Lynn: 6210, 6250
Martorella, Geraldine: 4490
Marx, Katherine: 3310
Marziliano, Allison: 4700
Masaki, Kamal H.: 3580
Maslow, Katie: 4150
Mason, Aleigha: 1530
Mason, Lisa Reyes: 6500
Mason, Tyler: 6580
Masoud, Sara: 2420
Massa, Fernando F.: 9020
Masse, Sybil: 1160, 3660, 6610
Massihzadegan, Setarreh: 6700
Massimo, Lauren M.: 1270, 3680, 3930, 5030, 6280
Mast, Benjamin T.: 3251, 6290, 9010
Ma, Stefan: 9020
Mastel-Smith, Beth: 9010
Masternak, Michal: 9000, 9010
Masterova, Kseniya S.: 6740
Mastrandrea, Carmelo: 4120, 6780
Mathavan, Nandini: 6590
Mathis, Kenneth: 4490
Ma, Tianzhou: 2300
Matos-Moreno, Amilcar: 4905, 6790
Matsumoto, Yoshiko: 4470
Matsushita, Kunihiro: 4330
Matta, Sonali: 5173
Matta, Tara D.: 3360
Mattek, Nora: 4790
Matteson, Tyler J.: 3190
Matthews, Alicia K.: 6430
Matthews, Karen A.: 3070
Mattos, Meghan: 9010, 9020
Matusovsky, Bri: 2690
Mauldin, Rebecca: 9030
Maung, Kenwin: 6330
Maurer, Jürgen: 2680, 3720, 4480, 6550, 6730
Mauss, Iris B.: 2620
Mau, Theresa: 3340, 9010, 9020
Maxfield, Molly: 9000, 9020
Maxi, Andrea: 6420
Maxwell, Cathy A.: 1180, 3450
Maxwell, Christopher D.: 1390
Maxwell, Colleen: 9000
Mayeda, Elizabeth R.: 3510, 3600
Ma, Ying: 6500
May, Jennifer: 9000, 9020
Maynard, Madison H.: 9030
May, Peter: 3200
Ma, Yu: 6100
Mazan-Mamczarz, Krystyna: 1470
Mazzola, Paolo: 5010

Mbao, Mbita: 6130
McAdams-DeMarco, Mara: 4680
McAlister, Jennifer: 6280
McAndrews, Carolyn: 5172, 9020
McAtee, Robin: 6000, 6130, 6210
McAuley, Edward: 9010
McBride, A. Camille: 6770
McCabe, Donna: 2130
Mccaffery, Jeanne: 3400
McCain, Dextiny: 2380
McCall, Stephen: 2710
McCann, Brandy Renee: 9020
McCarthy, Catherine: 9020
McCarthy, Ellen: 9000, 9020
McCarthy, Michael J.: 9030
McCauley, Brenna: 1470
McClain-Moss, Lisa: 6320
McClean Cooke, Wendy: 4030
McCleary, Lynn: 1420
McClellan, Angela: 5173
McClintock, Martha K.: 1230, 6110
McCloskey, Rose: 3555
McClure, Erin A.: 9000
McClure, Leslie: 4870
McConnell, Eleanor S.: 1130, 6110, 6280, 6450, 9010, 9020, 9030
McConnell, William R.: 4860
McCray, Rachel: 6680
McCulloch, Charles E.: 6320
McCurry, Darcy S.: 4150
McDaniel, Jodi: 5090
McDaniel, Kennedy A.: 4680
McDarby, Meghan: 2030, 6730, 9010, 9030
McDowell, Cynthia: 6590
McDuffie, Danielle L.: 1490
McEniry, Mary: 4225, 6200
McFarland, Lisa: 9020
McGaffigan, Erin: 4270, 6120
McGarry, Brian E.: 6140
McGee, Jocelyn S.: 5172, 5173, 6650
McGill, Cassandra: 9000
McGill-Scarlett, Cordy: 4681
McGilton, Katherine: 3170, 6270
McGinn, Michael: 6790
McGovern, Ian: 6100
McGowan, Deric: 4200
McGrew, Kathryn: 4430
McGuire, Lisa C.: 1560, 2490, 3120, 3510, 4120, 5010, 6060, 6280
McGuire, Patricia: 3930
McHugh, John P.: 3130, 3482
McHugh, Molly: 9000
McInnes, Keith: 9010
McIntyre, Alissa C.: 6100
McKay, Tara: 1320
McKelvey, Karma: 5083
McKenzie, Brennen: 9010
McKibbin, Christine: 3460
McKiddy, Sarah: 1150
McKinney, Brandee: 6410
McLaren, Jaye: 1040
McLaughlin, Jessica K.: 2720
McLaughlin, Sara: 9010
McLennan, Lauren K.: 6680
McLeod, Don: 4720
McLeskey, Nanci K.: 2060
McMahan, Christopher: 4500
McManus, Benjamin: 3000
McMillan, Corey T.: 6010, 6140
McMillan, Ryan: 5091
McMurray, Josephine J.: 2460
McNair, Bryan: 6280
McNally, James: 6830
McNamara, Kelsey C.: 6790, 9010
McNamara, Maeve: 1140
McNeil, John J.: 4320, 4683, 5083
McNicoll, Lynn: 9030
McPhee, Patrick: 2500
McPherson, Rachel: 6170
McPhillips, Emily: 9020
McPhillips, Miranda: 3310, 4080, 6300, 6600
McQuade, Laura: 3380
McQuaid, Tina A.: 6130
McQuaige, Mary: 5171
McQuarrie, Angelica: 3620
McRae, Thomas D.: 3830
McSpadden, E.J.: 9020
McSweeney-Feld, Mary H.: 9000
Meador, Lauren: 6470
Medcalf, Robyn: 9030
Medina, Luis D.: 1440, 6060
Meeks, Huong: 4530
Meeks, Suzanne: 2330, 3910, 9010
Meer, Margarita: 1140
Mehegan, Laura: 1430, 6150
Mehling, Wolf: 3050
Mehrabi, Fereshteh: 2580
Mehranfard, Danial: 6320
Mehta, Jade S.: 6590
Mehta, Neil: 4905, 6210, 6330, 6790
Mehta, Ranju: 6750
Mehta, Rhea: 6780
Mei, Donghui: 9010
Meier, Clément: 2680, 3720, 6550, 6730
Meier, Helen: 3430, 6010
Meier, Tabea: 3150, 4570
Meijers, Judith: 1160, 3555, 4720, 6270, 9010
Meinertz, Naomi R.: 2440
Meinow, Bettina: 6200
Mejía, Shannon T.: 1240, 2050, 2220, 3490, 9020, 9030
Mejia Arango, Silvia: 4225, 6300
Melendez, Robert: 2570
Melgar Castillo, Andrea I.: 4220
Melis, Rene: 5170
Mellencamp, Kagan A.: 1300
Melters, Daniel: 1200
Melton, Colleen C.: 6500
Meltzer, Gabriella Y.: 4110, 6090
Melvin, Tonisha: 3330
Mendenhall, Alexander R.: 3810
Mendes de Leon, Carlos: 4905, 6790
Mendez, Jennifer C.: 6220
Mendez, Nicole: 6720
Mendiratta, Priya: 6180
Mendoza Ruvalcaba, Neyda Ma.: 6290, 6380
Mendoza, Lisandra: 6140
Mendoza, Mayra: 2420
Mendoza, Nancy: 3740
Menec, Verena: 6790
Mengel-From, Jonas: 4810, 6411
Menges, Carolyn: 6420
Meng, Hongdao: 3540, 3910, 4650, 6070, 6290
Menne, Heather: 4770
Menon, Shreya: 6460
Meraz, Rebecca: 5173, 6650
Merchant, Anwar: 6320
Mercury, Monica: 2410
Merdjanoff, Alexis A.: 6090
Merianos, Ashley L.: 2250, 6710
Merickel, Jennifer: 6420
Merkin, Sharon Stein: 6440
Mernitz, Sara E.: 6190
Merrilees, Jennifer: 4681, 4850
Merten, Natascha: 2670
Meszaros, Katherine: 9000
Metcalf, Emily E.: 1040, 6420
Metcalf, Judith: 3540
Methot, Stephen: 1200
Metzelthin, Silke F.: 6270
Meyer, Anthon: 6270
Meyer, Cassie: 5083
Meyer, Kayla: 6570
Meyer, Kylie: 2070, 2420, 4510, 4790
Meyer, Michelle L.: 9030
Meyers, Jeremy: 6840
Meyer, Sonya: 9020
Mezuk, Briana: 1160, 4630, 5060
Miao, Hongyu: 4490
Michael, Cherish: 6250, 6470
Michael, Esposito: 1050
Michalek, Joel: 6320
Michelet, Mona: 1190, 6800
Mickle, Angela: 2300
Mielenz, Thelma: 5171
Mielke, Michelle: 6450, 6780
Migliaccio, John N.: 4880
Migneault, Deborah: 1310
Mignot, Emmanuel: 4560
Migovich, Miroslava: 5090
Miguel-Cruz, Antonio: 6390, 6560
Mikton, Christopher: 9000
Milani, Sadaf A.: 6710
Miles, Toni: 4460
Milidonis, Mary: 6420
Milisen, Koen: 6560
Miljkovic, Iva: 4340, 6450
Millar, Roberto J.: 1250
Millenbah, Ashley N.: 6070
Miller, III, Edgar R.: 4680
Miller, Carrie A.: 6650
Miller, Edward A.: 1260, 2040, 3320, 6620
Miller, Emika S.: 3360, 4650, 5090
Miller, Erin: 6220
Miller, Hillary: 6010
Miller, James W.: 2280
Miller, Julie: 3450, 4090, 6310
Miller, Katherine: 6110
Miller, Luke R.: 6830
Miller, Lyndsey: 4790, 6080, 6520

Miller, Matthew: 3050
Miller, Maura: 6000
Miller, Michael E.: 2530
Miller, Sally M.: 3450
Miller, Sara: 6260
Miller, Sarah J.: 9010
Miller, Vivian J.: 6090
Mills, Carol: 6760
Mills, Sarah: 5060
Millstein, Joshua: 3510
Milnamow, Mary: 6390, 9030
Mimbs, Hope: 6250
Minard, Charles G.: 2241
Minch, Kendra: 9010
Min, Deborah: 3330
Mindlis, Irina: 3115
Mingo, Chivon A.: 4555, 6180
Min, Judy: 1320
Min, Jungkyung: 6650
Minor, Lisa: 6360
Mintus, Kenzie: 3690
Mintzer, Jacobo': 2245
Minyo, Morgan J.: 9020
Miranda, Fabio: 3880
Mirza, Noeman: 4020
Mirza, Raza: 6230, 6480, 6690
Mitchell, Barbara: 6530
Mitchell, Brendan A.: 4200
Mitchell, Jamie: 6180
Mitchell, Kimberly M.: 1250
Mitchell, Lauren L.: 4220
Mitchell, Susan: 1530
Mitin, Natalia: 2241
Mitteldorf, Josh: 5082
Mitzner, Tracy L.: 4160
Miwa, Hiroyasu: 6390, 6400
Miyawaki, Christina E.: 6530
Mizuki, Maiko: 6240
Mlinac, Michelle E.: 2030
Mobley, James: 2501
Mock, Colleen: 6550
Mockler, David: 3200
Moeller, Stacey: 6060
Moen, Phyllis: 6800
Moermans, Vincent R.: 6560
Moffat, Scott D.: 6710
Moffit, Reagan: 4600
Mogle, Jacqueline: 2430, 2470, 2530, 3520, 3730, 6260, 6290
Mohr, David C.: 9030
Mois, George: 1570, 3410, 6000, 6390
Molina, Anthony J.: 4461, 6010, 9010
Molinari, Victor: 6540
Molinsky, Jennifer: 3900, 4380
Moll, Allison C.: 6440
Molnar, Lisa J.: 3000, 6470
Molony, Sheila L.: 4681
Monaghan-Geernaert, Pamela: 2210
Monahan, Patrick O.: 6280
Monaro, Sue R.: 2090, 6280
Monheit, Alan C.: 1260
Monin, Joan K.: 3360, 4850, 9000
Monroe-Lord, Lillie: 6210
Monroe, Todd B.: 3360, 4650
Montano, Anna Rae: 4770
Montano, Monty: 6641
Montelpare, William: 6250
Montepare, Joann M.: 1070, 2350, 3420, 3800, 6000
Montesano, Rachel: 4681
Montgomery, Lucas: 5080
Monti, Stefano: 2501
Montminy, Joe: 4681
Montoro-Rodriguez, Julian: 6750
Montoya, Ana: 9010
Moo, Lauren R.: 1040, 6420
Moone, Rajean: 1320, 2060, 6360
Moonesinghe, Dilhara: 9030
Mooney, Kathi: 1320, 2540
Moon, Heehyul: 4420
Moore, Alison M.: 9000
Moore, Ann Z.: 4200
Moore, Carley: 6230, 6690
Moore, David J.: 3760, 9020
Moored, Kyle D.: 3690, 3750, 4600
Moore, Jade T.: 6360
Moore, Miranda: 1170
Moore, Raeanne C.: 2450
Moore, Reese: 2340
Moorman Li, Robin: 9030
Moorman, Sara M.: 2400, 3570
Moppett, Iain: 9030
Moradoghli, Fereshteh: 3620
Morales Mejia, Yenifer L.: 6050, 6410
Morales, Knashawn H.: 6180
Moray, Juno: 6480
Moreland, Briana: 4100
Morelli, Alexandra: 2350
Moreno, Monica: 4010, 6130, 9030
Moreno, Oscar: 6140
Morey, Miriam C.: 4260, 5050, 6170
Morgan, Amanda: 6300
Morgan, Brianna: 1440, 3680, 3930, 4610, 9000, 9030
Morgan, Damian: 2460
Morgan, Gail D.: 6750
Morgan, Jennifer C.: 6760
Morgan, Kerri: 2050
Morgan, Matthew R.: 2700
Morissette, Lee F.: 4390
Morlett Paredes, Alejandra: 9000
Morley, John: 3600
Moro, Tatiana: 6740
Moro, Teresa: 2560
Morrill, Kristin A.: 6430
Morris, Brian J.: 3580
Morris, Emily P.: 1240
Morris, Kevin: 9020
Morris, Michael: 6420
Morrison, Sean: 4460, 5030
Morrow-Howell, Nancy: 3800, 9010
Morse, Janice M.: 1120
Mortensen, W Ben: 6500
Mortman, Nadav J.: 6641
Morton, Patricia M.: 5060
Mor, Vincent: 4780
Moskowitz, Judy: 2300
Mosley, Thomas: 2670, 3110, 3560, 6780
Mosqueda, Laura: 5100, 6580
Moss, Karen O.: 3360, 4650, 5090
Mossman, Audrey: 4680
Mota, Miriam: 6450
Motel-Klingebiel, Andreas: 2410
Moulder, Robert Glenn: 1020
Mouton, Charles: 5100
Mo, Vennisia: 6170, 9020
Moxley, Jerad: 3410, 9030
Moye, Jennifer: 2030, 2660, 3850, 6220
Moyo, Patience: 4580
Mpofu, Elias: 1090, 6110, 9020
Mroczek, Daniel K.: 2140, 2620, 4620
Mroz, Emily L.: 1490, 3360, 6340
Mroz, Tracy: 6260
Muñoz, Elizabeth: 3890, 4730, 6180, 9000
Muñoz-Lobato, Fernando: 6411
Mu, Christina X.: 4080, 4560, 6200, 6600
Mudar, Raksha A.: 3410, 6290, 6300, 6390
Mueller, Christine: 3170, 1060, 1460
Mueller, Kimberly D.: 2160
Mueser, Kim: 6170
Mui, Ada C.: 6500
Mukhi, Priya: 6310
Mukhtar, Yerkebulan: 4684
Mulcahy, John: 5130, 6540
Mulhorn, Kristine A.: 2260, 2680, 6220
Mullen, Nadia: 6170
Mullholand, Mary Ellen: 6390
Mullin, Donncha S.: 6450
Mullins, C. Daniel: 4220
Mullins, Lindsay: 6770, 9000
Mumma, Kara T.: 4160, 9030
Mundadan, Reanne G.: 6460
Mundy, Lindsey M.: 1240, 9020
Mungas, Dan M.: 3510, 3600
Muniz Terrera, Graciela: 2140, 3510, 5084, 6440, 6450, 9020
Munkácsy, Erin: 2501
Mun, Kyung-Ryoul: 9020
Munly, Kelly A.: 2050, 9000
Munoz, Richard A.: 1330
Munsterman, Ellen: 9000
Murabito, Joanne M.: 3400, 6450, 6740, 9020
Muramatsu, Naoko: 3890
Murata, Chiyoe: 6250
Murayama, Hiroshi: 6280, 6800
Murff, Harvey: 6740
Murillo, Fernando: 5120
Murillo, Rosenda: 2170
Murphey, Yi Lu: 4700
Murphy-Boucher, Michele: 9030
Murphy, Dallas J.: 6780, 9010
Murphy, Ian E.: 2010
Murphy, James D.: 6200
Murphy, Kyle A.: 4160
Murphy, Sara: 1250
Murphy, Terrence E.: 3110
Murray, Anne: 5083
Murray, Briana: 9000
Murray, Laura: 6160, 6460
Murray, Sarah: 6100
Musallam, Noura: 6210

Musey, Paul: 9010
Musich, Shirley: 6170
Musil, Carol M.: 3115, 4060, 5140, 6090, 6820
Musi, Nicolas: 6020, 6320
Musolino, Teresa: 4023
Mussa, Kadija: 9030
Muss, Hyman: 6420
Mustafa, Muhammad Ibrar: 6690
Mutambudzi, Miriam: 6300
Mutchler, Jan: 6090, 6700
Mutoh, Tatsushi: 9000
Muto, Yoko: 6280
Mutter, Justin: 9020
Mwendwa, Denee: 6170
Myagmarjav, Sugarmaa: 4684
Myck, Michal: 2410
Myers, Dennis: 5173
Myers, Dillon: 6390
Myers, Evan R.: 9030
Myers, Jennifer: 6450, 6510
Myers, John: 1130
Myers, Julie: 1060
Myers, Sara: 6740

**N**

Na, Annalisa: 1120, 4870
Nabe-Nielsen, Kirsten: 6200
Nabors, Emily: 3900, 6230
Nadash, Pamela: 1260, 2040, 3320, 3940, 4130
Nadorff, Danielle K.: 2040, 3740, 6520, 6580
Naganathan, Vasi: 2090, 6280
Nagel, Corey L.: 6340, 6450, 6740
Nagele, Robert G.: 6411
Naglie, Gary: 6170
Nagwani, Amit Kumar: 9010
Nahabedian, Anissa: 6350
Nahm, Eun-Shim: 5171
Nah, Suyoung: 6210, 6250
Naik, Aanand D.: 4683, 6320
Naioti, Eric: 6320
Najafzadeh, Mehdi: 6641
Najarian, Cyrus: 3130, 6060
Naka, Alexander: 9010
Nakagawa, Kazuma: 3580
Nakagawa, Takeshi: 6210, 6250, 6760
Nakamoto, Isuzu: 6280
Nakamura, Masahide: 9000
Nakanishi, Yasuhiro: 2580
Nakase, Taizen: 9000
Nakashima, Taeko: 6120
Nakayama, Riko: 6240
Nakazawa, Atsushi: 6000
Nalawade, Vinit: 1390
Nam, Youngwon: 6520
Naranjo Rincon, Samuel D.: 6290
Narayanan, Nandakumar: 9020
Narine, Ameesha: 4555, 6380
Narine, Donnette: 4555, 6130, 6670, 6710
Narita, Miki: 6320
Naruse, Masatoshi: 9010
Nasar Kabir, Zarina: 6760
Nassauer, Joan I.: 6490
Natale, Ginny: 5173
Nathanielsz, Peter: 5001, 9010
Nathan, Kobi: 6270
Naughton, Michelle: 2200
Navarrete, Viridiana: 9010
Nawaz, Huma: 6320
Naylor, Mary: 6200, 9000
Nazroo, James: 9020
Nderitu, Sara: 3160
Nearing, Kathryn A.: 4260, 6420
Nease, Donald: 9000
Nebenzahl, Paul: 6540
Nedlund, Ann-Charlotte: 2460
Neelamegam, Malinee: 3550
Neff, Donna F.: 6140
Nehrkorn-Bailey, Abigail M.: 2040, 5110
Nekolaichuk, Erica: 4090
Neller, Sarah: 2310, 9000
Nelligan, Labrini: 6270
Nelson, Christi L.: 4730
Nelson, Christi: 4540
Nelson, Christopher: 6010
Nelson, Ehlana: 3040
Nelson, Eve-Lynn: 6650
Nelson, H. Wayne: 9000
Nelson, Ian M.: 5081
Nelson, James: 9010
Nelson, Katie: 3330, 6140
Nelson, Mark R.: 4683, 6320
Nelson, Matt: 1530, 3180
Nelson, Monica E.: 6600
Nelson, Niccole A.: 4555, 6380
Nelson, Sarah: 6270
Nelson, Toben F.: 6510
Nemati, Donya: 3630, 6080
Nemeth, Samuel: 6810
Neppl, Ronald: 5001
Nesser, Whitney: 9020
Netter, Molly: 1060
Netto, Jair: 6840
Netzer, Giora: 6350
Neugroschl, Judith: 5100
Neumann, Johannes: 4683
Neupert, Shevaun D.: 1290, 2140, 2470, 2750, 3490, 6710
Newman, Anne: 3340, 3400, 3830, 4120, 4340, 4410, 4683, 4810, 9010, 9020, 9030
Newman-Casey, Paula Anne: 9000
Newman, David N.: 4220
Newman, Jasmine: 3520
Newman, John C.: 4410
Newmyer, Lauren: 4070
Newsham, Tina: 4520, 6350, 9030
Newsom, Jason T.: 6340, 6450, 6740
Newton, Nicky: 3480
Ng, Crystal: 1100, 1450, 3730
Ng, Derek K.: 2270
Ng, Jenny Hong Yu: 6840
Ng, Kwun Kei: 6411
Ng, Michelle: 4570
Ngo, Van: 9000
Ngo, Victoria: 1330, 6310
Ng, Ted Kheng Siang: 2050, 3115
Ng, Tze Pin: 3450, 4023
Nguyen, An T.: 3750
Nguyen, Andrew H.: 1040
Nguyen, Annie: 4070, 6580
Nguyen, Duy: 3290
Nguyen, Giang: 3770
Nguyen, Uyen-Sa: 2080
Ng, Weiwen: 6540
Ng, Yan Er: 3241
Ng, Zoe Ziyi: 3250, 6210
Nichenko, Anna S.: 5091
Nicholas, Jennifer S.: 4190
Nichols, Amy: 4021
Nichols, Emily: 9020
Nichols, Emma: 2270
Nichols, Eunice Lin: 1080
Nichols, Madeline J.: 6520, 6580
Nicholson, Jody: 9000
Nicklas, Barbara J.: 2241, 4600, 6641
Nicolay, Sarah: 3530
Nicosia, Francesca M.: 3050
Nicot-Cartsonis, Mathilda: 9010
Nicotera, Nicole: 4430
Niedernhofer, Laura: 2501, 6840
Nielsen, Lisbeth: 3080
Nielsen, Natalia: 9020
Nieman, Carrie L.: 3560, 4050, 4680
Nijhuis-van der Sanden, Ria: 1120
Nikitin, Jana: 2480, 2750
Nikolich-Žugich, Janko: 6010
Nikpour, Jacqueline A.: 1530
Nilsen, Charlotta: 3440, 6200, 6450
Nimrod, Galit: 6390
Ninteau, Kacy: 6540
Nishioka, Yuichi: 2580
Nishizuka, Chiharu: 6220
Nitanai, Ryoichi: 6240
Nix, Caitlyn A.: 4021, 9000
Niznik, Joshua: 9030
Nketsiah, Ebow T.: 9000
Nkimbeng, Manka: 1050, 1480, 3310, 6540
Nkwata, Allan K.: 1410, 4700
Noblitt, Jessica A.: 6190, 6250, 6520
Noblitt, Paul: 6650
Noda, Tatsuya: 2580
Noel, Polly H.: 1330, 4010
Noel, Sabrina E.: 2610
Nofuji, Yu: 6320, 6710
Noguchi, Taiji: 6210, 6250, 6760
Noh, Hyunjin: 3220
Noh, Ji Yeon: 6010
Nohria, Raman: 1540
Nolan, Timiya: 2200
Nolte, Julia: 6520
Noppert, Grace A.: 6050
Norbeck, Cassandra S.: 9010
Nordheim, Johanna: 6110
Noriega de la Colina, Adrian: 3630, 6740
Norris, Haley: 1320
Norris, Madison: 9030
Norris, Nia C.: 6750
Norton, Edward: 3130
Norton, Peter G.: 4210, 4720
Nouryan, Chris: 9000, 9010
Novak, Joshua: 4850, 6410
Nowaczynski, Aliya: 4090
Nowakowski, Alexandra C.: 4681
Nowels, Molly: 1310
Numata, Hanako: 4440
Nuno, Miriam: 6110
Nuspl, Megan: 6270
Nwakasi, Candidus: 5171, 6430

Nwanaji-Enwerem, Ugoji: 9000
Nyamathi, Adey: 2070
Nyberg, Lars: 3590
Nygaard, Marianne: 6010
Nygard, Louise: 2460
Nyman, Rosita: 6640
Nyquist, Linda: 6470
Nyrop, Kirsten: 6420
Nyrup, Rune: 6640
O'Connor, Melissa: 6100, 9030
O'Donnell, Lauren: 9000

**O**

O'Barr, Gregory W.: 3460
Öberg, Peter: 1300
Obiagwu, Joseph: 4560
Obomsawin, Anik: 4490
O'Brien, Catherine: 9010
O'Brien, Christopher T.: 6640
O'Brien, Emma M.: 6280
O'Brien, Erica: 2470
O'Brien, Kelly M.: 2490
Occean, James: 1200
Ocegueda, Omar: 6010
Ochoa-Lopez, Andrea P.: 1440
O'Connell, Denise: 6270
O'Connell, Megan E.: 9000
O'Connell, Tom: 5001
O'Connor, Daniel: 6210
O'Connor, Deborah: 4860
O'Connor, Maureen K.: 1040
O'Connor, Melissa L.: 6780
O'Conor, Rachel: 1030, 4681
Oczkowska, Monika: 2410
Odden, Michelle: 1000, 3400
Odd, Kaleena: 2140
O'Dea, Eireann: 4860, 6500
Odgen, Lydia: 2050
Odom, Erica L.: 4120
Odom, Teresa L.: 6360
O'Donnell, Arden E.: 4430, 6730
O'Donnell, Jerry: 9020
O'Donoghue, Jennifer: 9010
Ofori, Edward: 9020
Ofotokun, Ighovwerha: 3760
Ogawa, Elisa: 5050
Ogawa, Kenichi: 6450
Ogbuoji, Osondu: 9030
Ogino, Ryogo: 6240
Ogle, Destiny: 6520
Ogletree, Aaron M.: 2580
O'Grady, Thomas: 6090
Ogunjesa, Babatope: 6530
Ogunrinde, Joyce O.: 6300
O'Hara, Courtney: 9000
O'Hare Kelly, Erin: 6420
Oh, Esther S.: 4050
Oh, Gahee: 9020
Oh, Janet: 1080
Oh, Jeewon: 1240, 2280
Oh, Minsuk: 6510, 6650
Oh, Sungho: 6100, 6390
OKADA, Shinichi: 9010
Okahashi, Sayaka: 6210
Okeahialam-Iheanyi, Chidi: 6390
O'Keefe, Patrick: 3440, 3510, 6440
Okoye, Safiyyah M.: 2580
Okuzono, Sakurako S.: 4690
Oladejo, Dianne: 4681
Olanisa, Olawale: 2510, 6240
Olanrewaju, Sherif A.: 6700
Olatunji, Samuel A.: 9020
Olds, Danielle: 2720
Oleary, Bryant: 1280
Oliva, Anthony A.: 6320
Olivari, Benjamin: 3510, 4120, 5010, 6280, 6680
Oliveira, Eduardo: 6700
Oliveira, Iara R.: 3640, 4350
Oliver, JoAnn S.: 6100
Oliveros, Gianna: 6770
Olivia, Landon: 1300
Oliviera, Iara: 4350
Olivieri-Mui, Brianne: 5084
Olmos, Brenda: 1060
Oloo, Alice: 6530
Oloruntoba, Oluyomi: 6710
Olsen, Bonnie: 4890, 5173
Olson, John D.: 9010
Olson, Marin G.: 3740, 6520, 6580
O'Malley, Kelly: 2660, 4770, 6220
Omar, Zaliha: 1060
Omenyi, Chiazam L.: 4680
Omeragic, Faris: 6470
Omobowale, Olubukola C.: 6100
O'Muircheartaigh, Colm: 2550
Omura, John D.: 2490, 4120, 6280
O'Neill, Desmond: 3380, 3585, 9010
O'Neil, Moira: 2100
Ong, Anthony D.: 2300, 3730, 4620
Ong, Lydia Q.: 2430
Onken, Brian: 9010
Ono, Mayuko: 6580
Opsasnick, Lauren: 1030
Opute, Amaka E.: 9030
Orange, Joseph: 6160, 6460
Orchard, Suzanne: 5083
Orekoya, Afolabi: 2510, 6240
Orewa, Gregory N.: 3660, 4820
Orji, Gloria: 6430
Orkaby, Ariela R.: 3600, 4683, 5084, 6320
Orlina, Edmund Cedric A.: 3483
Orlovsky, Irina: 6210, 6411
Ornstein, Katherine A.: 1160, 1190, 1350, 4460, 4530, 4860, 5030, 5070, 6310, 6530
Orom, Heather: 6510
Orsega-Smith, Elizabeth: 4021
Orsulic-Jeras, Silvia: 6150, 6370, 6390
Ortega, María: 1550
Ortenberg, Elia K.: 1220
Orth, Jessica: 1390
Ortiz, Cristina: 6100
Ortiz, Kasim: 2390
Ortmeyer, Heidi K.: 5050
Orwoll, Eric: 9030
Ory, Marcia: 6310, 9030
Osada, Hisao: 6580
Ó Súilleabháin, Páraic S.: 4620
Oshana, Jodie: 6210
Osha, Peyton: 9020
O'Shea, Brendan Q.: 2270, 4110
Osiecka, Zuzanna: 6070, 6200
Osokpo, Onome H.: 6200, 9000
Osorio Castaneda, Camilo A.: 6090
Ospina-Romero, Monica: 1050
Østbye, Truls: 1540
Osteen, Kathryn: 6650
O'Sullivan, Julie L.: 6110
O'Sullivan, Roger: 1270, 2690, 3380
Osuna, Margarita M.: 6830
Osvold, Lisa: 3460
Oswald, Austin: 2390, 2540
Otgon, Saranchuluun: 4684
Otto, Robin B.: 9030
Overbeck, Kevin: 6560
Owen, Donna: 6760
Owoaje, Eme T.: 3900
Owsiany, Montgomery T.: 9010, 9030
Owusu, Cynthia: 6510
Oya, Akari: 3480
Oyebode, Abiodun O.: 6500
Oyebode, Jan: 1270
Oyinlola, Oluwagbemiga: 6100
Ozdemir Van Dyk, Semra: 3160
Ozgur, Tosun: 6530

**P**

P. Corona, Ligiana: 9020
Pace, Sarah: 9030
Pacheco, Kelly: 5150
Packard, Benjamin: 6270
Padeken, Jan: 1200
Padron, Dilianna: 6140
Pageau, Félix: 6400
Pagedar, Nitin A.: 3550
Pagoto, Sherry: 6450, 6650
Pai, Manacy: 2400
Pajerski, Dawn: 9030
Pajewski, Nicholas M.: 4150
Pak, Karla: 6480
Palacio, Ana: 6420, 6140
Palan Lopez, Ruth P.: 1530
Palen, Ted: 3360
Palgi, Yuval: 1160
Pallaki, Muralidhar: 9030
Palmer, Janice L.: 6100
Palmer, Lauren: 3160
Palmer, Nathaniel: 9000
Palm, Rebecca: 3090
Palms, Jordan D.: 1240
Palta, Priya: 4050
Panchagnula, Sree Saroj Sainath: 9000
Pan, Diane: 9000
Pandya, Naushira: 6220
Pan, Fifi: 9010
Pang, Celeste: 6480
Pangle, Amanda: 6130
Panicker, Amina: 1120
Panier, Hunter A.: 9010
Pankey, Arianna: 1470
Pankow, James S.: 2670, 3400
Pan, Men-An: 4450
Pan, Stephanie: 3400
Pantalon, Michael: 5100
Pantleay, Harveen: 4510
Pan, Wei: 6110, 9030
Pan, Xi: 2080
Pan, Yiming: 4290
Paoletti, Jensine: 9010
Paolillo, Emily W.: 2450
Paone, Deborah: 4310
Papa, Carol-Lynne: 4890
Pappadis, Monique R.: 5100

Pardasani, Manoj: 3290
Parekh, Rupal M.: 6250
Parham, Cheryl: 4260
Parianos, Mary: 3483, 4021
Parinandi, Narasimham: 5090
Parish Johnson, Julie: 1330
Park, Amy: 1350, 6320
Park, Catherine: 4683, 6320, 9020
Park, Chan Mi: 9000
Park, Cherrie: 3740
Park, Crystal: 9000
Parker Oliver, Debra: 6730
Parker, Andrew: 6440
Parker-Autry, Candace: 6780
Parker, Daniel: 2241
Parker, Danitra: 2501
Parker, Elizabeth A.: 5050
Parker, Lauren J.: 3310
Parker, Madison: 9030
Parker, Martha: 6710
Parkes, David: 1430
Park, Geunhye: 1210, 4021
Park, Grace: 1180
Park, Hayoung: 6390
Park, Hong-Jae: 4430
Park, Hyeyoung K.: 9030
Park, Hyunjung: 6810
Park, Jeongsoo: 2480
Park, Jihye: 4320
Park, Jina: 4370, 5150
Park, Joon-Hyuk: 2290, 3840
Park, JuYoung: 1030, 1550, 2210
Park, Juyoung: 6180, 6810
Park, Ki Tae: 3220
Park, Mei Linn N.: 3220
Park, Min Kyoung: 9000
Park, Mina: 9020
Park, Moonkyoung: 6320, 9010
Park, Nan Sook: 3220, 6490, 6810
Park, Pil: 9010
Park, Seho: 6580, 9000
Park, Sojung: 4370, 5150, 6240, 6800, 9000, 9010, 9020, 9030
Park, Soobin: 6800, 9000, 9020
Parks, Reid: 9010
Park, Taeyoung: 6690, 9030
Park, Yoobin: 6830
Parmar, Jasneet D.: 1420
Parnia, Sam: 2160
Parraga, Carla B.: 6140
Parr, Aubrey: 9030
Parslow, Chad: 3330
Parsons, Kelly: 1380, 6060, 6720
Parsons, Pamela L.: 2260
PART Working Group: 6010
Partap, Angie: 3000
Parthasarathy, Sairam: 6510
Pasatiempo, Ana Maria: 6210
Pascual-Leone, Alvaro: 3482, 3510
Pasquini, Giancarlo: 6550
Passos, Joao: 3241
Patel, Darpan: 6390
Patel, Monika: 9030
Patel, Prakash: 9020
Patel, Sandip Kumar: 3241
Patel, Sanjay R.: 2620, 4080
Patel, Sheena: 9020
Patel, Susan: 5050
Patel, Sweta: 3830, 4465
Pate, Melannie: 6640
Pathiravasan, Chathurangi H.: 9020
Pat, Lian Ying Chun: 4684, 5084
Patorno, Elisabetta: 6110, 6641
Patrick, Julie H.: 2040, 9020
Patskanick, Taylor: 3260, 3450, 4090
Patterson, Sarah E.: 5070, 6310
Patti, Gary J.: 4810
Paudel, Anju: 2230
Paul, Constanca: 6560
Paulsen, Adam J.: 2670
Paulson, Daniel: 4480, 9030
Paulson, Sally: 6450, 6510, 9000
Paul, Sudeshna: 6310
Pauly, Theresa: 3150, 6210, 6390
Pavlidis, George: 2410
Pavlovic, Noelle: 3330
Pavon, Juliessa: 9030
Pawlowicz, Abigail: 5171
Payne-Foster, Pamela: 6100
Payne, Laura: 5082, 5130, 6170
Peña, Miguel: 6510
Peach, Laura: 6270
Pearce, Rahul C.: 6050
Pearsall, Vesselina: 3241
Pearson, Megan: 4260, 5050
Peckham, Allie: 9000
Peden-McAlpine, Cynthia: 9000
Pedersen, Ingeborg: 4300
Pedersen, Megan: 6780
Peeler, Anna: 6140
Peeri, Noah: 2080
Pees, Suzanne: 9020
Pei, Heming: 9010
Pei, Yaolin: 1130, 1540, 3481
Pelton, Leslie: 2560
Pencina, Karol: 6641
Pendedo, Frank: 9000
Pendergrast, Claire: 4770
Peng, Chagmin: 2040
Peng, Changmin: 9020
Peng, Lei: 9010
Peng, Man-Man: 4682
Peng, Siyu: 6690
Peng, Siyun: 3570, 6220
Penna, Victoria: 6470
Penney, Lauren S.: 1330, 4010, 6390, 6420
Peppard, Paul E.: 4560
Perales Puchalt, Jaime: 2070
Perez Montes, Viviana: 9010
Perez, Emily M.: 2260
Perez, G. Adriana: 3060, 6300, 9000
Perez, Hector: 6390, 6560
Perez, Janelle: 4210
Perez, Viviana I.: 1470
Perianayagam, Arokiasamy: 1060
Perissinotto, Carla: 2690, 6790, 9010
Perissi, Valentina: 4001
Perkhounkova, Yelena: 2520, 5010
Perkins, Anthony: 6580
Perkins, Fatima: 9020
Perkins, Molly M.: 3290, 3760, 4030, 5173, 9000, 9010, 9020
Perls, Thomas T.: 2501, 4810, 6010
Perone, Angela: 2730, 4820, 6060
Perreault, Hannah: 4340
Perre, Taylor: 6720
Perry, Brea L.: 3570, 4860, 6220
Perry, Katrice M.: 6180
Perry, Marieke: 5170
Perry, Tam E.: 3860, 4380, 6180
Perweiler, Elyse: 6560
Perzynski, Adam T.: 2640, 4100
Pessin, Lea: 4070
Pestine-Stevens, Althea: 2010
Pesut, Barb: 3500
Peszka, Jennifer: 6210
Peters-Beumer, Lisa: 3000
Petersen, Curtis: 6780
Petersen, Ron: 6780
Peters-Nehrenheim, Viktoria: 3610
Peterson, Andrew: 2160
Peterson, Colleen M.: 4220, 6510
Peterson, Donna: 6280
Peterson, Jennifer: 2260
Peterson, Lindsay J.: 2720, 4465
Peterson, Mark: 2500
Peterson, Matthew: 1060, 9030
Peterson, Natasha L.: 2200, 4905, 6790
Peterson, Rachel L.: 3600
Peterson, Ryan A.: 6470, 9030
Peters, Ruth: 9000
Petkus, Andrew J.: 3510
Petrillo, Kelsi: 6510
Petrovčič, Andraž: 1210
Petrovsky, Darina: 6110, 6600
Pfeffer, Bruce: 6100
Pfund, Gabrielle N.: 2430, 4620
Pham, Catherine: 3050
Phelan, Elizabeth: 6260
Philipp, Ian: 6010
Phillips, Drystan: 6830
Phillips, Karon L.: 3120, 6350
Phillips, Kristin: 6420
Phillips, Patrick: 9010
Phillips, Stuart: 3620
Phillips, Will: 4520
Phinney, Alison: 4860
Phongtankuel, Veerawat: 9030
Piazza, Jennifer R.: 2430, 2470, 2660, 3730
Picard, Martin: 4001
Picchiello, Matthew C.: 6160
Piccinin, Andrea: 2140, 6290
Picella, David V.: 9030
Pickering, Andrew M.: 2501, 4461, 5091
Pickering, Carolyn E.: 1390, 2250, 6110, 6390, 6590, 6750, 9000
Piechota, Amanda: 3360, 4850, 9000
Piedra, Lissette M.: 2550
Piercy, Cameron W.: 1570
Pierett, Jason: 3540
Piersol, Catherine: 2150
Pike, Christian: 9000, 9020
Pilla, Scott: 3400

Pillemer, Karl A.: 2000, 4090, 6310, 6480, 6520, 6530, 6690
Pimentel, Camilla M.: 1040, 4770
Ping, Yongjing: 1000
Pinheiro, Cristina N.: 6590
Pini, Lorenzo: 1030
Pinkert, Christiane: 3090
Pinto, Jayant M.: 1230, 3560, 6110
Pipinos, Iraklis: 6740
Pirtskhalava, Tamar: 6840
Pivodic, Lara: 1270
Plamondon, Katrina: 4020
Plante, David T.: 4560
Plassman, Brenda L.: 4420
Plaza-Rodriguez, Alma: 6411
Pless Kaiser, Anica: 2660, 6220
Plotzke, Michael R.: 1310, 6720
Plude, Dana: 1220
Plummer, Sherry: 4890
Plys, Evan: 4650
Pluth, Jack: 5001
Pocnet, Cornelia: 6380
Podgorski, Carol: 4860
Podlesek, Anja: 6090
Poe, Abigail: 2250, 6170
Poghosyan, Lusine: 1530
Pohl, Janet S.: 2250
Pohnert, Anne: 1530
Polivka, Larry: 4130
Polk, Thad A.: 4040
Pollino, Madison: 4022
Polson, Clay: 5173
Pomeroy, Mary Louise: 3130
Ponce, Julian: 6350
Pond, Brittney L.: 9020, 9030
Pondikos, Maria: 6420
Pond, Richard S.: 6350
Pongan, Lauren: 6530
Pool, Natalie: 9030
Poon, Cecilia: 2030, 3850
Poon, Lydia: 6390
Pope, Caitlin N.: 2450, 3000, 4225, 6060, 6200, 9020
Popejoy, Lori: 3170, 6270
Pope, Natalie E.: 5080
Popescu, Mihail: 4021
Poplawski, Antonina: 6400
Popov, Vladimir: 9000
Porterfield, Shirley L.: 9010
Porter, Kristie A.: 3640, 4350
Porter, Michelle M.: 6780, 9010, 9030
Portugal, Gabriela: 9010
Possin, Katherine: 6270
Post, Wendy: 3400
Poteat, Tonia: 1400
Potempa, Kathleen: 4870
Poulos, Michael: 6840
Poulsen, Rachel A.: 6420
Pourkhalili, Azin: 5171
Powell, Danielle: 2670
Powell, Nathaniel: 2560
Powers, Becky: 6320
Powers, Daniel: 6260
Powers, John David: 3360
Powers, Sara M.: 6150, 6370, 6390, 6530
Pradhan, Rohit: 3660, 6420
Prasad, Anyah: 2400
Prasun, Marilyn: 9030
Preddie, Alaina: 6390
Preisser, John: 1160
Prendergast, Alice: 6090
Prentice, Kailee: 6050, 6410
Prescott, Mike: 6500
Pressman, Peter: 4850
Presti, Peter: 4160
Pretzer-Aboff, Ingrid A.: 6420
Prevodnik, Katja: 1210
Prevratil, Michael: 9020
Price, Ashley N.: 6710
Price, Catherine C.: 4840, 6320
Price, Debora: 6310
Price, Elvin: 6320
Prideaux, Matt: 5001
Prigerson, Holly G.: 1490
Primack, Abby S.: 3810
Prina, Matthew: 1000
Priyambada, Priya: 6180
Prochaska, John: 9020
Procter-Gray, Elizabeth: 2290
PROMISE Study Group: 3160
Proper, Karin: 9020
Prost, Stephanie Grace: 5120
Proulx, Christine M.: 6680
Pruchnicki, Alec: 9010
Prusaczyk, Beth: 3310
Pruzin, Jeremy: 9000
Prvu Bettger, Janet: 4260
Puca, Annibale: 6010
Puckett, Amanda: 4461
Puerto, Geraldine: 6140
Puga, Frank: 2250, 6170, 6390
Pugh, Jacqueline A.: 1330
Pugh, Kelly: 6740
Pugh, Kendra: 1040
Pugh, Mary Jo: 1330
Pukhraj, Ambri: 6430, 6530
Pullen, Carly: 6140
Pullon, Emerald: 5173
Punke, Elizabeth: 3460
Punksungka, Wonmai: 4555, 6130, 6670, 6710
Puntambekar, Mehak: 9010
Purol, Mariah: 2280
Purwins, Daniel: 3090
Putnam, Michelle: 2050
Puts, Martine: 1420
Puurveen, Gloria: 3500
Pyles, Karly: 6580
Pynoos, Jon: 3900, 6230

**Q**

Qiao, Yujia: 4600, 9030
Qin, Tian: 4700
Qin, Weidi: 1160, 3860, 6730
Qin, Yongmei: 6490
Qin, Yue: 6710
Qiu, Paddy: 6120
Qiu, Xiao: 4820
Qi, Xiang: 1130, 1540, 3115, 3481, 3870
Quach, Emma D.: 1040
Quach, Lien: 2080
Qualls, Sara: 6750
Qualter, Pamela: 2690
Quashie, Nekehia T.: 4690, 6300
Quevedo, Alvaro: 9000
Quijano, Keara: 9000
Quillen, Ellen E.: 9010
Quinn, Margaret M.: 2710
Quinn, Natalie: 4520
Quiñones, Ana R.: 1090, 2300, 3310, 6340, 6450, 6740
Quinones-Visot, Emily: 6390, 9030
Quintero Silva, Laura: 9020
Quintero, Yadira Y.: 6080
Qu, Shan: 1070, 9020

**R**

Rababa, Mohammad: 6640
Rabinowitz, Jill A.: 4330
Rachmuth, Lisa: 9000
Raciti, Audrieanna E.: 6770
Radhakrishnan, Kavita: 6260, 9020
Rados, Robert: 6090
Raetz, Jaqueline G.: 5010
Rafford, Meghan: 2250
Rafie, Carlin: 5171
Rahemi, Zahra: 4500
Rahimighazikalayeh, Gelareh: 9010
Rahman, Anna: 3390
Rahman, Hafizur: 6740
Rahman, Momotazur: 4780, 5081
Raimondo, Marianne: 6810
Rainville, Chuck: 6150, 6480
Rait, Greta: 3555
Rajagopalan, Subashini: 5070
Rajagopal, Shilpa: 6270
Raji, Mukaila: 9000, 9010
Raj, Minakshi: 9020
Ramachandran, Vasan S.: 3400
Ramadhani, Widya A.: 4170, 6240, 6300
Ramalingam, Pradeep: 6840
Ramasamy, Ramalakshmi: 6641
Ramasubramanian, Ramya: 6010
Ramaswamy, Ravishankar: 4250
Ramdas, Kevin N.: 6320
Ramesh, Durga: 9020, 9030
Ramirez Gomez, Liliana A.: 4190
Ramirez, Javier E.: 6420
Ramirez, Madieska: 9000, 9010
Ramirez, Mildred: 2160
Ramirez-Ruiz, Mara: 6170
Ramirez-Sanchez, Israel: 9010
Ramirez, Valerie: 4190
Ram, Nilam: 3150, 4570, 6170
Ramos, Katherine: 2300
Ramos, S. Raquel: 2130
Ramos, Sofhia V.: 9010
Ramsdale, Erika: 6270
Ramsey, Jennifer: 6750
Ramsey, Jon: 1470
Randa, Cari: 3450
Randall, George K.: 6620
Randhawa, Paavan: 1550
Rangghran, Parisa: 6641
Ranka, Sanjay: 6510
Rankin, Katherine P.: 9000
Ransome, Yusuf: 9020
Rao, Sunil V.: 5050
Rapoport, Andy S.: 4021, 4023, 4270
Rapoport, Mark: 6170
Rasekaba, Tshepo D.: 3550
Ratcliff, Ellen: 9010
Rathbun, Alan: 6780
Rathfon, Megan: 6460
Ratnayake, Maggie: 9010
Rauer, Amy: 3480, 4030, 6170
Rauktis, Mary: 6680
Rausenberger, Alex: 5001
Ravichandran, Sathyabaarathi: 3001
Ravitch, Sharon: 3680, 3930

Raymond, Jennifer: 4390
Razavi, Marianne: 2550
Razjouyan, Javad: 4683, 6320, 9020
Reaves, Steven: 2560
Reblin, Maija: 2070, 4510
Rebok, George: 2530, 4680, 5110
Reckrey, Jennifer: 1180, 1190, 5030, 5070, 6530, 9030
Reczek, Rin: 4225, 6200
Redd, Doug: 6770
Redfern, Mark S.: 3690
Redman, Leanne: 2180
Redmond, Leslie C.: 6270
Redondo, Andres: 9020, 9030
Reed, Nia: 6280
Reed, Nicholas S.: 2530, 2670, 3560, 4050, 6710
Reel, Candice D.: 2030
Reese, Jordan: 4150
Reese-Melancon, Celinda: 9020
Reese, Melanie: 4023
Reeves, Alexis: 2080, 6140
Reeves, Jennifer: 6270
Reeves, Mathew: 4770
Regenbogen-Weiss, Sandy: 9000
Rehman, Aziz: 6600
Reid, Cary: 2300, 5150, 6310, 9010, 9030
Reid, Christopher M.: 4320
Reider, Lisa: 2580
Reid, Kerry: 4620
Reilly, Dermot: 2690
Reilly, Elizabeth: 9000
Reilly, Paula: 9000
Reinhardt, Joann P.: 6100
Reisdorf, Bianca C.: 1210
Reither, Eric N.: 4560
Remillard, Elena T.: 4160, 9030
Rendell, Peter: 9020
Renna, Megan E.: 4470
Rennke, Stephanie: 6460
Rentscher, Kelly E.: 3400
Reres, Adam: 6700
Resciniti, Nicholas V.: 1000, 6320
Resnick, Barbara: 2230, 3090, 3650, 3830, 4760, 6170, 6350, 9020
Resnick, Neil: 3350
Resnick, Susan M.: 3510, 4200, 4330, 4340
Reuben, David B.: 4150
Revenson, Tracey: 3115
Reyes, Adriana M.: 4420
Reyes, Laurent: 3290, 5080
Reyes, Mariana: 6060, 6220, 6280
Reynolds, Addam: 5130
Reynolds, Chandra A.: 9000, 9010, 9020
Reynolds, Christina: 2450
Reynolds, Courtney: 4770, 4900, 5083
Rhoads, Timothy W.: 1140
Rhodes, Annie S.: 1110, 2510, 6140, 9010
Rhodes, Shanae: 2420
Rhodus, Elizabeth: 1380, 4790, 4870, 6060
Rhoten, Bethany: 1180
Riaz, Muhammad: 6280
Richards, Bradley: 1310
Richards, Kathy: 6260, 9020
Richards, Nicole E.: 3730
Rich, Jodi: 9030
Richman, Alice R.: 3100
Richmond, Therese S.: 6390
Rich, Stacey D.: 3550
Rick, Chelsea: 6270
Rickenbach, Elizabeth: 1260
Riediger, Michaela: 2470
Riemenschneider, Falk: 3620
Riffin, Catherine: 2000, 6310, 6530, 6690
Rikhtehgaran, Farinaz: 6500
Riley, Alicia R.: 4480
Ring, Lauren: 4360
Rink, Andrea: 5172
Ripat, Jacquie: 6780, 9010
Risacher, Shannon L.: 3570, 6820
Risbud, Rashmi D.: 1330, 6430, 6530
Ritche, Christine S.: 5070
Ritchie, Christine S.: 5030
Ritter, Ashley Z.: 4360
Rittiwong, Tanapa: 6770
Rivera Almaraz, Ana: 4290
Rivera-Hernandez, Maricruz: 1090, 4780
Rivera, Rebecca L.: 6270
Rivera-Torres, Solymar: 6130
Rizer, Sandra: 6260
Rizzo, Matthew: 6420
Rizzuto, Debora: 3590
Roach, Ashley: 1530
Roache, Tavia: 9000
Roach, James: 1250
Roach, Lauren: 4225, 6200
Robbins, Hilary: 9000
Robbins, Nathaniel M.: 2670
Robbins-Panko, Jessica C.: 6370, 6830
Robbins, Paul: 2501, 6840
Roberson, Tonya: 4681
Roberto, Karen A.: 1220, 3240, 9020
Roberts, Amy R.: 4830, 6260
Roberts, Ellen: 1110, 9030
Roberts, Eric: 9010
Roberts, Jimmie: 9030
Roberts, Josephine: 6080
Roberts, LeAnna: 6180
Roberts, Lisa: 9000
Roberts, Mitchell: 6390
Robertson, Andrew D.: 4120, 6780
Robertson, Anna C.: 6170
Roberts, Shelby: 6060
Robert, Stephanie A.: 5172, 9020
Roberts, Tonya J.: 3520, 9010
Robinette, Jennifer W.: 3440
Robinson-Ector, Kaitlynn: 2300
Robinson, Erin L.: 1210, 3760, 4021
Robinson, Kristen: 3160
Robinson-Lane, Sheria G.: 2420, 3495, 3890
Robison, Julie: 1310, 2040, 2150
Robison, Raele: 3830
Robitaille, Annie: 6150, 6210
Robling, Alexander G.: 6840
Röcke, Christina: 1020, 2450, 3150
Rockwood, Kenneth: 1180
Rodakowski, Juleen: 1340
Rodgers, Joeseph: 3440, 3510, 6440
Rodin, Rebecca: 4460, 5030
Rodi, Scott: 4390
Rodríguez Díaz, Melina: 6290, 6380
Rodriguez, Diana: 5110
Rodriguez, Elisa M.: 6430
Rodriguez-Gonzalez, Maribel: 6000
Rodriguez, Gustavo: 6140
Rodriguez, Miriam J.: 6140
Rodriguez, Misha: 4360
Rodriguez, Sonia L.: 6010
Roe, Lauren S.: 2290
Roes, Martina: 1270, 3090, 3610, 4270, 4610
Roessler, Meghann E.H: 6820
Rogers, Casey: 9010
Rogers, Geoff: 4090
Rogers, Stephanie: 6270, 9000
Rogers, Wendy A.: 1570, 2050, 3410, 3670, 4160, 4190, 6000, 6240, 6300, 6390, 9020, 9030
Rogozinski, Amos: 2680
Rogina, Blanka: 5001
Rogus-Pulia, Nicole: 3830
Rohner, Ronald P.: 1290
Rohner, Shauna L.: 4225, 6580, 5100
Roldan, Lanyce: 6100
Rollandi, Isabel Olivia: 9030
Romanelli, Meghan: 4540
Roman, Youssef: 6320
Romeo, Evelyn: 9000
Romero, Cristy: 5171
Romero, Ryan: 6140
Rommerskirch-Manietta, Mike: 3090
Romo, Rafael: 1030
Ronneberg, Corina R.: 6620
Rooks, Ronica N.: 2730, 3430
Roopsawang, Inthira: 3840
Roose, Steven P.: 3600
Root, Eve: 6130
Roquet, Angélique: 6380, 6580, 6790
Rorai, Vanessa: 6180
Rosaldo, Manuel: 6700
Rosano, Caterina: 1050, 3250, 4340, 6450, 9030
Rose, Andrew: 6620
Rosebush, Christina E.: 1480, 4060, 6540
Rose, Jacob: 3241
Rose, Johnie: 6510
Rose, Karen M.: 3360, 4650, 5090
Roseman, Jeffrey: 5173
Rose, Miriam: 4770, 4900, 5083, 6370
Rosemond, Cherie: 4360
Rosenberg, Molly: 3040, 9010
Rosenberg, Walter: 2560
Rosenblum, Sara: 9020
Rosendaal, Nicole: 3200, 5081
Rosenkranz, Susan: 6830
Rosen, Sonja: 5083
Rosenstein, Olivia: 1120
Rosen, Tony: 4900, 9000, 9030
Rosich, Rosellen M.: 1380, 4710
Rosko, Ashley: 3550
Rosland, Ann-Marie: 9000
Ross, Clifford: 4225, 6200

Rosselli, Monica: 4220
Ross, Lesley: 3270, 9020
Ross, Leslie: 6530, 9030
Ross, Madeline: 9000
Rosso, Andrea L.: 1050, 3250, 3690, 4340, 4600, 6450, 9030
Ross, Sarah E.: 1110, 1250
Ross, Serena: 9000
Ross, Webster: 3580
Rosteius, Katharina: 4300
Rote, Sunshine: 3020, 3240, 4420
Roth, Adam R.: 4860
Roth, David L.: 2630, 5110
Rothermund, Klaus: 2480
Rothschild, Anthony J.: 9010
Rotondi, Armando: 9010
Round, Teri: 5172
Rowan, Noell L.: 1320, 6350
Rowe, John W.: 6330
Rowe, Meredeth: 6760
Roy, Deepa: 9020
Roy, Indrakshi: 4780
Roy, Upal: 9020
Rozario, Philip A.: 2240
Rubinstein, Stephanie R.: 6440
Rubtsova, Anna: 3760
Rudolph, James: 3830, 3130
Rudroff, Thorsten: 2245
Rudy, Ellen T.: 6790, 9010
Ruffin, Adrienne: 6760
Ruggeri, Siena: 3940
Ruggiano, Nicole: 6750, 9000
Ruggiero, Laurie: 4021
Ruiz, Martha: 6510
Rule, Payton: 6160
Runge, Katherine: 2690
Rupper, Randall: 1330
Rupprecht, Fiona S.: 2480, 2750
Rurka, Marissa: 4070
Rusch, Amber V.: 6200
Rush, Jonathan: 2430, 2470, 3730, 6290, 6580
Rush, Kathy: 4020
Russell, Andrea: 2280
Russell, Dan: 9020
Russell, David: 1190
Russel, Wynfred: 1050, 1480, 6540
Russ, Tom: 6450
Rutherford, Bret R.: 3600
Ruth, Terrance: 9030
Rutkowsky, Jennifer: 1470
Rutledge, Emily: 6390, 6560
Ryan, Alice: 6780
Ryan, Cayley E.: 6610
Ryan, Joanne: 4320, 6320
Ryan, Lindsay H.: 3430, 3480
Rydberg Sterner, Therese: 3590
Ryd, Charlotta: 2460
Rymaszewska, Joanna: 5170
Ryou, Yeon Ji: 4140, 4905, 6790
Ryser, Valerie-Anne: 4480, 6550
Ryskina, Kira: 2090
Ryu, Ahyun: 6320
Ryu, Byeongju: 4370, 5150, 6240, 9000, 9010, 9030
Ryu, Jinhyung: 9020
Ryuno, Hirochika: 6100, 6110
Sörensen, Silvia: 9010

S

Sabat, Steven R.: 1380
Sabharwal, Anup: 9000
Sabino-Laughlin, Eilleen: 2070
Sabra, Ahmad: 1350
Sabuncu, Crim: 9030
Sacco, Paul: 4510
Sachdev, Perminder S.: 4340
Sachs, Barrie: 9010
Sachs, Greg: 4100, 6280
Sadak, Tatiana: 1150, 6260
Sadarangani, Tina: 2130, 3060, 9020
Sadeq, Nasreen: 6000
Sadowski, Cheryl: 6270
Saeidzadeh, Seyedehtanaz: 4720
Saenz, Joseph: 3040, 4420, 9000, 9020
Sagong, Hae: 6180
Saguez, Rodrigo: 6320
Sahawneh, Fursan: 9020
Sahli, MIchelle W.: 4023
Sahoo, Nirakar: 9020
Sahoo, Shalini: 6490
Saifuddin, Fatema: 6411
Saini, Manik: 2600
Saito, Tami: 6210, 6250, 6280, 6760
Saito, Yasuhiko: 6290, 6600, 9030
Sajatovic, Martha: 6160, 6510
Sajwani, Zara: 6770
Sakamoto, Mariko: 4860, 9000
Salafia, Caroline: 9000
Salas, Aileen: 4225, 6580
Salas, Joanne: 3600
Saleem, Ahsan: 6270
Saleem, Zainab: 6640
Salem, Yasser: 6780
Saliba, Debra: 1460
Salinas Rodríguez, Aarón: 4290
Salinger, Maggie R.: 5030
Salmon, Adam: 5001, 6010, 9010
Salom-Bail, Shirley: 6210, 9030
Saloniki, Eirini-Christina: 6700
Salvador, Andrea Francesca: 4461
Salvo, Erin: 4090
Salzman, Talia: 4340
Samant, Rahul S.: 5091
Sama, Susan R.: 2710
Samper-Ternent, Rafael: 3020, 4150, 6330
Sample, Alanson P.: 9000
Sampson, Carla: 9010
Sampson, Elizabeth l.: 3555
Sampson, Joshua: 4560
Sampson, Laura: 6830
Sampson, Natalie: 6490
Samuel, Laura J.: 2510, 3560
Samuel, Raja: 6090
Samus, Quincy: 4280
Sanchez-Reilly, Sandra: 9000
Sanchez, Ricci: 4681
Sandell, Lindsay L.: 9020
Sanders Schmidler, Gillian D.: 9030
Sanders, Beth: 6150, 6390
Sanders, Bethany E.: 3770
Sanders, Edie C.: 3670
Sanders, Jason: 3400
Sanders, John W.: 6100
Sanders, Lisbeth: 6370
Sanders, Margaret: 1190, 3530
Sandhu, Shobhana: 9020
Sandoval Garrido, Felipe A.: 9000
Sands, Bryan: 3810
Sands, Talyah: 3010, 6060
Sanford, Angela: 6260
Sanford, Jon A.: 4160
Sang, Elaine: 6100
Sanko, Stephen: 6770
Sanmartin Berglund, Johan: 3590
Sanner, Caroline: 1300
Sanni, Mubarak: 2300
Santana, Leticia: 2560
Santanasto, Adam J.: 3400, 4120, 4340, 4600, 4810
Santora, Kenneth: 3080, 3280
Santos, Flavia H.: 1060
Sanz Simon, Sharon: 6420
Sapozhnikova, Anna: 4850
Sappington, Erica: 6390
Saraboon, Yuwadee: 3840
Sardina, Angie L.: 3270, 6350
Sargent, Lana: 6140, 6320
Sarif, Nawaj: 2150, 6610
Sarkar, Abhirup: 6411
Sarkar, Nilanjan: 5090
Sarker, Ananya: 6390
Sarna, Kaylee: 6160
Sarnevitz, Janelle: 1070
Sarshar, Arash: 5171
Sass, Alexandra: 6180
Satake, Anna: 9020
Satizabal, Claudia L.: 9020
Satoh, Kana: 4440
Sauer, Jacquelin: 9030
Sauerteig-Rolston, Madison R.: 6290, 6810
Saunders, Michele J.: 3870
Saunders, Pamela A.: 4180
Savage, Kirstie M.: 2060
Savary, Patricia: 6130
Savich, Tatjana T.: 4020
Saviers, Brittany: 1420
Savla, Jyoti: 3240, 9020
Savundranayagam, Marie: 2520, 5000, 6160, 6460
Sawhney, Amitoj: 1470
Sawyer, Linda M.: 6420
Saxena, Divya: 6780
Sayed, Linda: 3430
Sayer, Tina B.: 3000
Saykin, Andrew J.: 3570, 6820
Scales, Kezia: 2710
Scanland, Susan: 6260
Sceppa, Carmen: 6790
Schönstein, Anton D.: 5110
Schaaf, George W.: 9010
Schaefer, Sarah K.: 5110
Schaeffer, James: 6170, 6580, 6790
Schafer, Marissa J.: 3241, 6010, 6411
Schafer, Markus H.: 6790
Schaff, Branden: 6220
Schapira, Lidia: 6430, 6530
Scheckler, Samara: 3900, 4380
Scheel, Jennifer: 4300
Scheffer, Julian A.: 4681, 9000
Schell, Brent R.: 9000
Schenck, Sabine: 3460
Schenkel, Rachel: 9000

Schepens Niemiec, Stacey L.: 4420
Scher, Clara: 1170, 5130
Scherer, Danette: 6000
Scherer, Hailey: 6140
Scherer, Philipp E.: 4241
Scherrer, Jeffrey: 3600
Scherzer, Rebecca: 6320, 6780
Schiaffino, Melody K.: 3670, 3750
Schiller, Gabrielle S.: 1160, 3660, 6320, 6610
Schilling, Birgit: 3241, 6840, 9010
Schilling, Oliver K.: 1020
Schiltz, Nicholas K.: 6510
Schilz, Matthew: 4240
Schipf, Sabine: 1010
Schmid, Arlene A.: 2280, 3930
Schmidt, Annika: 4300
Schmidt, Laura I.: 2750
Schmidt, Nicholas E.: 2030, 3850
Schmitz, Wiebke: 9020
Schneider, Haley: 3240
Schneider, Joanne: 6770
Schnoll, Roseanne: 9010
Schoenborn, Nancy: 2200
Schols, Jos: 6270, 9000
Schousboe, John T.: 9030
Schrack, Jennifer A.: 4050, 4120, 4330, 4600, 6740
Schriner, Sam: 6010
Schroeder, Matthew W.: 6390, 6450, 6580, 6650, 9000, 9030
Schroepfer, Tracy: 6720
Schubert, Carla R.: 2670
Schubert, Cathy C.: 6390
Schukle, Jamie S.: 4260
Schulman-Green, Dena: 1540
Schulte, Phillip: 6450
Schultz Lee, Kristen: 4070
Schultz, Linda: 6100
Schultz, Michael: 6641
Schultz, Rebecka: 6560
Schultz, Savannah R.: 9000
Schulz, Marc: 2620
Schulz, Richard: 4090
Schumacher, Benjamin T.: 9030
Schumacher, John G.: 6350, 9010
Schuman, Donna: 6250
Schumm, L.Philip: 2550
Schupf, Nicole: 6010
Schurman, Charles: 3241
Schuster, Amy M.: 4020, 9030
Schut, Bibi: 4460
Schut, Henk A.W.: 4460
Schutte, Brian C.: 4470
Schutte, Debra L.: 4470, 6420
Schwab, Delphine Roulet: 6400
Schwartz, Abby J.: 3100
Schwartzkopf, Ashley: 6390
Schwartz, Todd: 6760
Schwarzberg, Arielle: 1100
Schwingel, Andiara: 6530, 9020
Scott, Adrienne W.: 3560
Scott, Judith M.: 6770, 9010
Scott, Mallary: 3100
Scott, Rachel K.: 3740, 6520, 6580
Scott, Stacey B.: 3250, 3440, 4620, 6550
Scrivano, Rachel: 2350, 3780
Seale Reiff, jenni: 4680
Seaman, Aaron T.: 3550
Sears, Dorothy D.: 9020
Sears, Jeanelle: 4420
Sebastiani, Paola: 2501, 4810, 6010
Seckin, Gul: 6430
Sedore, Christine: 9010
Seeman, Teresa E.: 3440, 4560, 6440, 6580
Seetharaman, Kishore: 3450, 6500
Sefcik, Justine S.: 1120, 3310, 5020, 6110, 6390, 6600, 9020
Segal, Daniel L.: 6550, 6770
Segelman, Micah: 3640, 4350
Segerstrom, Suzanne C.: 5140, 6590
Sehgal, Raghav: 1140
Seifert, Allison M.: 1420
Seifert, Imke: 5170
Seino, Satoshi: 6320, 6710
Seixas, Azizi: 6680
Sejdic, Ervin: 3690, 4340
Sekhar, Rajagopal V.: 2241, 9010
Seki, Yuichi: 9000
Seligman, Benjamin: 4690, 6320, 9020
Sellers, Christina: 6350
Sellon, Alicia: 9030
Semelka, Charles T.: 6100
Semprini, Jason: 3660
Sen, Payel: 1200
Seo, Dongchul: 6780
Seo, Jin Young: 6480
Seo, Jisu: 6320
Seong, Hohyun: 5171
Seo, Shinae: 9010
Serdaly, Christine: 2090
Serra, Monica C.: 4260, 6390
Serrao Hill, Melanie M.Y: 6580
Servat, Caroline: 3540
Sesek, Greg: 9000
Seshadri, Sudha: 9020
Sesso, Howard D.: 3600
Severance, Jennifer J.: 1110, 1250, 6130
Sexson, Kathryn: 9000
Seyfried, Nicholas: 6840
Seyyedmirza, Paria: 6310
Shaaban, C. Elizabeth: 1050
Shadel, Doug: 6480
Shadmi, Efrat: 1160
Shadyab, Aladdin: 2200, 4560
Shaffer, Kelly: 9010
Shagalow, Shaina: 6260
Shah, Avani A.: 6180
Shah, Avantika: 4580, 6270
Shah, Priya: 3010, 6060
Shah, Raj C.: 6320
Shah, Rishabh: 6430
Shah, Sheetal: 6180
Shake, Matthew: 6570
Shakya, Iju: 4100
Shalaby, Alaa: 9010, 9030
Shalev, Idan: 5060
Shambley-Ebron, Donna: 6140
Shanbhag, Meera L.: 4680
Shang, Erhua: 6250
Sharafkhaneh, Amir: 9020
Shardell, Michelle: 3110, 6780
Sharit, Joseph: 3410
Sharma, Ayush: 6840
Sharma, Hari: 3950, 6360
Sharma, Shakshi: 6840
Sharma, Shubam: 3360, 4555, 6340, 6380
Sharp, Julia L.: 2250, 2280, 3930
Sharrett, Richey A.: 3560
Shatterly, Jaclyn: 4022
Shavit, Yochai Z.: 3190, 6170
Shaw, Clarissa: 2520, 2530, 5010
Shaw, Philip: 6170
Shawver, Zared: 3440
Shay, Kimberly A.: 2310
Shayya, Ashley: 6090
Shean, John: 3010, 6060, 6680
Shea, Steven: 3400
Sheck, Isabelle N.: 6350
Sheftel, Mara G.: 6300
Sheikh-Bahaei, Nasim: 9000
Sheikh, Sophia: 9030
Sheils, Natalie: 6110
Sheldon, Marisa: 3770
Shelley, Mack: 3500
Shellito, Natalie: 3320
Shende, Shraddha: 6390
Sheng, Chen: 6200
Shen, Huei-Wern: 6120
Shen, Jenny Y.: 4580, 6270
Shepherd, Alyx L.: 9000
Shepherd-Banigan, Megan: 6110
Sheppard, Christine: 4090
Sheppard, Luvon: 6280
Sherman, Aurora M.: 3480, 6580
Sherman, Ledric: 2250
Sherwood, Laura: 6350
Shewmaker, Peter: 6230, 6360
Shewokis, Patricia: 5020
Shi, Changyou: 1200
Shields, Cleveland: 9030
Shields, Pamela D.: 5090
Shields, Wendy: 9010
Shieu, Bianca: 6760
Shifflett, Benjamin: 6411
Shigenobu, Kazue: 9030
Shih, Patrick: 9020
Shi, Jiaming: 9000
Shi, Changyou: 1200
Shi, Ling: 1280
Shimabukuro, Michio: 6010
Shimada, Chiho: 6580
Shin, Donghee: 6240
Shin, Hyung Eun: 6450, 6510, 6740
Shin, Jacqueline C.: 9010
Shin, Jinhee: 6320
Shin, JiWon: 5090
Shin, John J.: 3560
Shin, Ju Young: 6760
Shinkai, Shoji: 6320, 6710
Shin, Marlena: 4770
Shin, Oejin: 9030
Shin, So Young: 6270, 9000
Shin, Yeonsoo: 6820
Shippee, Nathan: 6540
Shippee, Tetyana P.: 1050, 1480, 2650, 3310, 3910, 4130, 5130, 6540
Shirk, Steven D.: 1040
Shiroma, Kristina: 6730
Shirsat, Nikita: 3050
Shi, Sandra M.: 9020

Shi, Xiang: 6810
Shi, Ying: 6110
Shkirkova, Kristina: 9020
Shmuel, Shahar: 4320
Shofner, Sabrina J.: 2200
Shreeniwas, Sudha: 2730
Shrestha, Aman: 6750, 9010
Shrestha, Laxman: 4250
Shrestha, Srishti: 2670
Shrira, Amit: 3490
Shrout, M. Rosie: 3150, 4470
Shugrue, Noreen: 1310
Shulyaev, Ksenya: 1160, 2680, 9030
Shuman, Stephen K.: 3870
Shum, Eric Ngai Yin: 4022, 4684
Siaton, Bernadette: 6220
Sibille, Kimberly: 2300
Sibley, Kathryn: 6780, 9010
Sidani, Souraya: 6270
Siddiq, Hafifa: 3430
Sideman, Alissa: 9000
Sidey, Leanza: 9030
Sidhu, Amat: 6220
Sidoli, Simone: 1200
Sieber, Frederick: 1010
Sieber, Stefan: 6580, 6790
Siebert, Jelena S.: 5110
Siedlecki, Karen: 6760
Siegel, Elena O.: 2680
Siegler, Eugenia L.: 3000
Sieverding, Monika: 2750
Sigurdardottir, Sigurveig H.: 6220
Sihapark, Siranee: 4700
Sikder, Abu: 4190
Silberberg, Mina: 9000
Silbersack, Johanna: 3910
Silbert, Lisa C.: 2450, 4870
Sillner, Andrea: 2680, 3720, 6420
Silva, Graziele: 9020
Silva, Isabella: 9000
Silva, Jennifer: 3130
Silva-Smith, Amy L.: 6650
Silva, Susan G.: 9010
Silver, Emily: 9030
Silverman, Michael Alan: 6000
Silver, Michelle: 4684
Silverstein, Hannah I.: 6730, 9010
Silverstein, Merril: 1450, 3500, 6520, 6790
Silverstein, Nina M.: 3100, 3800, 6000, 6090, 6510
Silver, Stephanie A.: 2160
Simcox, Judith: 4241
Simmons, Ames: 9020
Simmons, Sandra F.: 4580, 6270
Simning, Adam: 6140
Simon, Darby M.: 6290, 9010
Simone, Abigail: 6450, 6510
Simon, Lois: 2020
Simon, Michael: 2090, 6270
Simonsick, Eleanor M.: 4120, 4200, 4330, 4600, 6740, 9020
Simpson, Deborah: 6280
Simpson, Elizabeth: 2040, 3320
Simpson, Felicia: 3400
Simpson, Luke: 6210
Sims, Tai: 9000
Sims, Wynton: 6460
Sinah, Indranil: 5001
Sinclair, Christian T.: 6730
Sinclair, David A.: 1140, 6641
Sindi, Shireen: 3440, 6450
Singer, Dianne: 3495
Singer, Katelyn J.: 6290
Singer, Rachel: 4240
SIngh, Abanish: 1000
Singh, Anmoldeep: 3570, 6820
Singh, Mriganka: 9030
Singh, Nainwant: 6530
Singh, Priyanka: 6100
Singh, Roshni D.: 6140
Singh, Rufus: 6090
Singleton, Meki: 1400
Sinha, Indranil: 6840
Sinkler, Kateri: 5171
Sin, Mo-kyung: 5173
Sin, Nancy L.: 2430
Sinvani, Liron: 4700
Sioutas, Constantinos: 9020
Sirey, Jo Anne: 9030
Sison, Stephanie Denise M.: 6641, 9000, 9020
Siu, Albert: 3660
Siu, Joey Chung Yue: 4022, 4684
Sivakolunthuvel, Balamurugan: 6100
Sivers-Teixeira, Theresa M: 4890, 5173
Skanderson, Melissa: 6770
Skarphol, Tricia: 3910
Skemp, Eleanor A.: 6520
Skemp, Lisa: 1180
Skinner, Kelly: 6270
Skinner, Robbie: 2020
Skipper, Antonius D.: 6620
Skoblow, Hanamori F.: 4691, 6680
Skoog, Ingmar: 3590
Skow, Laura: 4120
Skubic, Marjorie: 4021, 6390
Slattum, Patricia W.: 2260, 3230, 6000, 6320
Slaug, Bjorn: 4220
Slaven, James E.: 6100, 6280, 6390
Slayday, Riki E.: 3250
Sliwinski, Martin J.: 3250, 6290, 6550, 6790, 9010
Sloan, Allyson E.: 6210
Sloane, Elliot: 9030
Sloane, Philip D.: 3910
Sloane, Richard: 5050
Sluys, Erica: 2300
Smail, Emily J.: 2170, 4840, 6510
Small, Brent J.: 1100, 3400, 6180
Smichenko, Juliana: 1160
Smirnov, Igor: 4461
Smith, Alex: 4460
Smith, Alexander k.: 5030
Smith, Claire E.: 1100, 2620, 4080
Smith, Emily: 6570
Smith-Greenaway, Emily: 9020
Smith, Gregory C.: 4060
Smith, Jacqui: 1410, 2220, 6050, 6580, 9020
Smith, Jason R.: 3510
Smith, Jennifer L.: 4060, 6250
Smith, Jessica: 6010
Smith, Kathrine A.: 9000
Smith, Kelly: 6210
Smith, Ken R.: 4530, 6310
Smith, Kevin M.: 4830
Smith, Lenora: 4870
Smith, Lindsey: 3200, 3910, 5081
Smith, Louisa H.: 5084
Smith, Matthew L.: 2250, 6310, 6710, 9030
Smith, Patsy: 1060
Smith, Phillip: 6641
Smith-ray, Renae: 6100
Smith, Rebecca: 6500
Smith, Sherri: 5140
Smith, Thomas: 4200
Smith, Thomas J.: 6710
Smit, Kirsten: 3555
Smits, Carolien: 3610
Smolen, Andrew: 9010
Smoyer, Karen E.: 3830
Sneed, Rodlescia S.: 1160, 3450, 3770, 6310
Snider, Christina A.: 6740
Snyder, Rani: 2560, 3940
Snyder, Yonda: 1190
Sobol, Adam: 9020
So, Christine: 3480, 6170
Sok, An: 6120
Sol, Ketlyne: 1240
Solomon, Phyllis: 4750
Solway, Erica: 3495
Somers, Virend: 6740
Somerville, Ceara: 1170, 2010, 6090
Sommerer, Yasmine: 1010, 6010
Sommet, Nicolas: 6200
Song, Huaxin: 6760
Song, Hwasung: 6670
Song, Jiaxiu: 6180
Song, Mi-Kyung: 6310
Song, MinKyoung: 3240
Song, Qian: 3040, 6200, 9020
Song, Rhayun: 6320, 9010
Song, Xiao: 4700
Song, Xiaowei: 1180
Song, Yu: 4683, 9020
Song, Yuhua: 9010
Song, Yuting: 2090, 4210
Song, Zeyuan: 2501, 4810, 6010
Son, Julie S.: 3270
Sonnega, Amanda J.: 9020
Sorkin, John: 6220
Soto, Elizabeth: 4470
Sotres-Alvarez, Daniela: 4330
Sousa, Susana: 6560
Spangler, Hillary B.: 4640, 6780
Spang, Robert P.: 6110
Spaulding, Trent: 4820
Spechler, Lily: 6210
Spetz, Joanne: 3950, 6530
Spindle, Natalie: 9030
Spini, Dario: 6200
Spinner, Ruth: 6100
Spira, Adam P.: 4330
Spira, Dominik: 6010, 6641
Spiro, Avron: 2620
Splaine, Mike: 9020
Splitstone, Matthew J.: 6000
Spoden, Natasha: 9000
Spradley, Laura: 6210
Sprague, Briana N.: 4340
Sprang, Rob: 1380
Springfield, Sparkle: 2200
Springstein, Tabea: 4570
Squillace, Marie R.: 3640, 4350
Sreenivas, Ramavarapu S.: 4160
Sreevalsan, Kavya: 6320

Srinivasan, Erica G.: 6000
Sritan, Sarawut: 2610
St. Goar, Carl: 9020
St. Louis, Renee M.: 6470
Stürmer, Til: 4320
Staal, Bart: 1120
Staben, Omar: 6490
Stachler, Eva: 6510
Stacke, Tobias: 3090
Stadler, Gertraud: 3070
Stainback, Maggie: 9010
Stallard, Eric: 6010, 6411
Stallings, Devita T.: 6100
Stanback, Brianne M.: 6000
Standridge, Natalie: 6670
Stanfield, Alyssa: 6000
Stange, Kurt: 2640
Stankovic, John: 5090
Stanley, Jennifer: 9000
Stansbury, Kim: 9030
Stark, Emma: 4650
Staton, Lori: 6280
Stavrinos, Despina: 3000
Stawnychy, Michael A.: 6100
Stawski, Robert S.: 6520, 6580
Steacy, Katarina: 5171
Stearns, Harvey L.: 9000
Stebbins, Rebecca: 6050
Stechuchak, Karen M.: 5083
Steckenrider, Janie S.: 6190
Steele, Jenessa: 6140
Steele, Joel S.: 4790
Steer, Christopher: 3550
Steering Committee, Brain Health in the Community (BHIC): 4860
Stefanacci, Richard: 4020
Stefanacci, Richard G: 6260
Steffen, Ann M.: 2030, 3251, 3850
Steffen, Melissa: 6100
Stehling, Elisa: 6770
Steinberg, Nessa: 9020
Steinhagen-Thiessen, Elisabeth: 1010, 3070, 4040, 6641
Steinman, Bernard A.: 3460, 6140
Steinman, Michael A.: 5030
Stelle, Charlie: 4022, 9020
Stenhaug, Ben: 3000
Stephan, Abigail T.: 3270
Stephens, Caroline E.: 4530, 6310
Stephens, Jacquelyn E.: 3150, 4570
Stepniak, Catherine: 1290, 6520
Steptoe, Andrew: 2270, 3210
Sterling, Madeline: 2710
Sterns, Harvey L.: 6000
Stern, Yaakov: 6420
Stessman, Jochanan: 6610
Stevens, Alan B.: 4150, 6100
Stevens, Erin: 3550
Stevens, Jen: 6280
Steward, Andrew T.: 2310, 4240, 4430
Stewart Steffensmeier, Kenda: 6100
Stewart, Heather G.: 1210
Stickel, Ariana: 9000
Stiefler, Susanne: 4300
Stiles, Robert: 6650
Stinchcombe, Arne: 6170, 9020
Stinnett, Sandra: 9000
Stirland, Lucy: 6450
Stoeckle, Rebecca J.: 4890
Stokes, Jeffrey E.: 1090, 2400, 4460, 6200, 9030
Stolfa, Claire: 6180
Stoll, Isabella: 5020, 9020
Stone, Arianna: 6720
Stone, Katie L.: 4080, 4560, 6200
Stone, Lisa E.: 6550
Stone, Robyn I.: 2010, 2720, 3640, 4360, 6210, 6760
Storms, Aaron D.: 6120
Stout, Jeffrey R.: 2290, 3840
Straker, Jane: 3520
Strand, Bjørn Heine: 1190, 6800
Stransky, Stephanie: 1200
Stratton, Lauren: 4010, 6130, 9030
Stratton, Sara K.: 4900
Strauch, Kim: 9030
Strauss, Anna Wiersma: 1450
Strayer, Andrea L.: 6641
Street, Lara: 6140
Striley, Catherine L.: 9020
Strobl, Carolin: 3115
Stroebel, Armin M.: 3090
Stroebe, Margaret S.: 4460
Strogatz, David: 5171
Strommen, Jane: 6780
Strong, Jessica V.: 6250, 6440, 6640
Strong, Randy: 9010
Strotmeyer, Elsa S.: 2670, 3690, 4120, 6650, 9030
Strough, JoNell: 6210
Struble, Laura M.: 4870
Stuart, Bruce C.: 9010
Stuart, Robin K.: 3670
Stuck, Amy: 6770
Stukova, Marina: 3483, 9030
Sturdevant, Diana: 1060, 5172
Subedi, Janardan: 9010
Sublett, Jennifer F.: 9000
Subramaniam, Savitha: 6420, 6570, 6780
Suda, Hitoshi: 4440
Sudore, Rebecca: 3050, 9030
Suen, Jonathan J.: 2530, 3560, 4680
Sugawara, Ikuko: 6580, 6800, 6810
Sugg, Maggie: 4820
Sugiura, Aki: 6770
Suitor, J Jill: 1290, 2440, 4070, 4460, 6520
Sul, Hei Sook: 4241
Suliburk, James: 2241
Sullivan, Darcy: 2720
Sullivan, Dennis: 6420
Sullivan, Jennifer L.: 4770
Sullivan, Kevin: 2670, 6780
Sullivan, Marlysa: 1550
Sullivan, Suzanne S.: 6110, 6280
Sundermann, Erin E.: 9000
Sun, Fei: 1170, 1500, 4023, 4270, 5150, 6060, 9000
Sung, Liwen: 4450
Sung, Pildoo: 3160, 4170
Sun, Haosen: 6790
Sun, Luyang: 1470
Sun, Minglong: 6420
Sun, Na: 4820
Sun, Peter: 6500, 9010
Sun, Rongjun: 1100
Suntai, Zainab: 5172, 9030
Sun, Winnie: 6270, 9000
Sun, Xiaoyi: 6411
Sun, Yuxiang: 6010
Super, Nora: 1560, 3540
Supiano, Katherine: 1320, 2540, 9010
Suppiah, Sumithra: 3160
Surachman, Agus: 3070, 3440
Surage, Jayshree: 1550
Sur, Aparajita: 3160
Suriaga, Armiel A.: 4580
Suri, Anisha: 3690, 4340
Su, Tai-Te: 2050, 2220
Sutphin, George L.: 6840
Sutton, MaryAnn: 6130
Sutton, Natalie: 9020
Su, Wei-Hsuan: 6010
Su, Yan: 9030
Su, YanJhu: 1070, 3100, 6090, 6450, 6510
Su, Yun-Pei: 6800
Svec, Joseph: 4905, 6790
Swagger, Phildra: 6140
Swanson, Marilyn: 9030
Swartz, Ann: 9010
Swartzell, Kristen: 9030
Swartzendruber, Lora L.: 2720
Sweeder, Logan A.: 2680, 3720, 6420
Sweeny, Brian: 4360
Sweerts, Lieke: 1120
Sweet, Elisabeth: 9030
Swenor, Bonnielin K.: 2530, 3560, 4690
Swierenga, Sarah: 4770
Swisa, Orel: 3490
Syed, Sumaiyah U.: 5060
Sylvers, Dominique: 6310
Sylvester, Donna: 6220
Syme, Robin: 2600
Synder, Joel: 4905, 6170
Szanton, Sarah L.: 2510, 3330, 3560, 4310, 4640, 4680
Szcześniak, Dorota: 5170
Sze, Lesley: 9020
Szto, Peter: 6350
Szymkowski, Amanda: 6280

### T

T.V., Sekher: 2150
Taani, Murad H.: 6780
Tabler, Jennifer: 3460
Tabon, Patrick K.: 5173
Tabuchi, Takahiro: 6280
Tadros, Clarissa: 6670
Taeckens-Seabaugh, Ashley: 2730, 9020
Taffet, George E.: 2241, 2740, 9010
Tahija, Nina: 6530
Taitel, Michael: 6100
Takagi, Emiko: 6000
Takahashi, Paul: 6110
Takano, Yumi: 9000
Takase, Mai: 6240, 6280
Takayama, Midori: 6580, 6810
Takemoto, Yoshito: 9010
Takeya, Yasushi: 6110, 6450, 6770
Takiue, Keigo: 9030
Taki, Yasuyuki: 9000, 9010
Talbot, C. Conover: 6411
Talegawkar, Sameera: 4200
Taler, Vanessa: 6290
Talley, Kristine: 9000
Talley, Susan A.: 6510

Talmage, Lexi: 2340
Tamariz, Leonardo: 6140
Tamburri, Nicholas B.: 6590
Tamla Rai, Vijaya: 6690
Tanaka, Toshiko: 4200, 9010
Tan, Alai: 5090
Tan, Chenxin: 3870
Tan, Chuen Seng: 3620
Tan, Ern Yu: 3390
Tang, Ada: 3620
Tan, Gamaliel: 4220
Tang, Bin: 2450
Tan, Genesis: 6070, 6200
Tang, Fengyan: 4240, 6680
Tang, Jennifer Y.M.: 6110
Tang, Junhan: 2170, 4320
Tangpricha, Vin: 3290
Tang, Weizhou: 6540
Taniguchi, Yu: 6320
Tan, Jaron: 6580
Tan, Jeremy: 3620
Tanner, Jackie: 6230
Tan, Ngiap Chuan: 3160
Tan, Sharon: 6480
Tan, Shyuan Ching: 3270
Tan, Su Ming: 3390
Tan, Tira: 3390
Tan, Vanessa: 4220, 9020, 9030
Tan, Veronique Kiak Mien: 3390
Tan, Xiao-Di: 6010
Tan, Yi Wen: 3160
Tappeiner, Waltraud: 1530
Tappen, Ruth M.: 4220, 4580
Tarabochia, Dawn: 1280
Tarasenko, Lisa: 3830
Tarbert, Rebecca J.: 4020
tarima, sergey: 9010
Tarraf, Wassim: 2510, 3430, 4040, 4420, 9000
Tarr, Julie E.: 6090, 6230
Tate, Ashley: 6210, 6250
Tate, Judith A.: 5090
Tate, Rio S.: 6180
Tatewaki, Yasuko: 9000, 9010
Tavakoli-Tabasi, Shahriar: 9020
Tavares, Jane L.: 3320, 5140, 6140
Tay, Djin L.: 4530, 6310
Taylor, Britain M.: 6590
Taylor, Catherine: 6540
Taylor, Janiece L.: 3330, 3750, 4680
Taylor, Miles: 2200, 9020
Taylor, Philip: 2460
Taylor, Tonya: 3760
Taylor, Tracy A.: 6100, 6280
Tay, Sarah Siew Cheng: 3160
Team, MiaGB Consortium: 9000, 9010
Teas, Elizabeth: 6080, 6790
Teasley, Kenneth: 4023
Teaster, Pamela B. Teaster B.: 3920, 4130
Tecson, Kristen: 6100
Teerawattananon, Yot: 9030
Tefera, Gashaye Melaku: 1210, 4021
Teixeira, Laetitia: 6560
Tell, Eileen J.: 1260, 3940
Telonidis, Jacqueline: 2060
Temirova, Alisha: 9010
Templin, Thomas: 4470, 6420
Teno, Joan: 3200, 3830
Teresi, Jeanne A.: 2160
Terhune, Katherina N.: 4520
Teri, Linda: 4540
Tesema, Naomi: 6110
Teshale, Salom: 3940
Teshome, Abraham Z.: 6630
Testai, Fernando: 9000
Teupen, Sonja: 1270
Tewary, Sweta: 6220
Tezcan-Guentekin, Huerrem: 3610
Thackeray, Anne: 1120
Thaitrong, Phatt: 4680
Thangwaritorn, Pilar: 6570
Thanik, Erin: 1350
Tharpe, Rita: 6160
Tha, Soe Han A.: 2690
Thayer, Erin K.: 4890, 5173
Thelusma, Naomi: 4681
Thiamwong, Ladda: 2290, 3840
Thibault, Olivier: 6411
Thielke, Stephen: 6450
Thierry, Amy: 6290
Thillainadesan, Janani: 2090, 6280
Thoma, Myriam V.: 4225, 5100, 6580
Thomas Hebdon, Megan C.: 2070
Thomas, Ariane: 6060, 6220, 6280
Thomas, Duncan: 6580
Thomas, Kali: 3130, 3200, 3910, 4465, 4580, 4780, 4820, 5081, 9000
Thomas, Karuna S.: 9020
Thomas, Kelsey: 9000
Thomas, Kristi: 6730
Thomas, Michael L.: 6200
Thomas, Patricia A.: 6290, 6810
Thomeer, Mieke B.: 4225, 6200
Thomley, Amelia: 9030
Thompson, Alisha R.: 6500, 9020
Thompson, Amber: 4480
Thompson, Atalie C.: 2530
Thompson, Ben: 9000
Thompson, Katherine: 6220
Thompson, Larry W: 6750
Thompson, Lena: 3240, 6830
Thompson, Roy A.: 9010
Thompson, Sheri: 9030
Thomson, Cynthia: 6430
Thomson, Donna: 6000
Thorne, Claudia: 1250, 9030
Thorne, Trina: 2090
Thornton, Marleen: 9020
Thorpe, Jr., Roland J.: 2380, 2510, 3330, 3440, 3560, 6180, 6290
Thorpe, Carolyn: 9000
Thorvaldsson, Valgeier: 4620
Thrane, Lisa: 9020
Thurston, Rebecca C.: 3070
Thyagarajan, Bharat: 4810, 6010
Thygesen, Lau C.: 4530
Thyreau, Benjamin: 9010
Tian, Chenxin: 6740
Tian, Junyan: 3270
Tian, Qu: 4120, 4200, 4330, 4340
Tian, Yi Jiao (Angelina): 6400
Tibirica, Lize: 6200
Tiecker, Ana Paula: 3620
Tien, Hsiao-Chuan: 2080
Tiernan, Chad: 6510
Tighe, Patrick J.: 4840, 6320
Timinski, John: 6170
Tingvold, Maiken: 2750
Tissot, Juliana T.: 6240
Tjia, Jennifer: 6140
Tkatch, Rifky: 6580, 6790
Tobey, Cara: 6840
Tobey, Leah: 6000
Tobin, Courtney Sinclair S.: 3440
Tobon Vallejo, Diana: 4340
Todorova, Irina: 6210
Toki, Hiroshi: 6110, 6450, 6770
Tolea, Magdalena I.: 6070, 6720
Toledo, Frederico G.: 4600, 9030
Toles, Mark: 1160, 3482, 6760, 9000
Tollman, Stephen M.: 3040
Tolosa, Ezequiel: 9000
Tombline, Gregory: 2501, 6840
To-Miles, Flora: 9010
Tomioka, Michiyo: 6580
Tomita, Naoki: 9000
Tompkins, Catherine: 2260, 4510, 6280
Tonkikh, Orly: 2680
Tonkin, Andrew M.: 4320, 4683, 5083
Toof, Robin: 2610
Toosizadeh, Nima: 6510
Toplyn, Lavona: 6560
Torgé, Cristina Joy: 6640
Torioge, Trevor: 6010
Torke, Alexia M.: 6100, 6280
Torrance, Blake L.: 9010
Torres-Gil, Fernando: 4450, 3770
Torres, Sandra: 2040
Torres, Stacy: 6200
Torrisan, Maral: 6740
Toruner, Murat: 9000
Toto, Pamela E.: 4310, 4600
Tourette, Cendrine: 9010
Touron, Dayna R.: 2750
Townsend, Michelly: 2610
Towsley, Gail L.: 2060, 2310, 9020
Towsley, Kerri: 9010
Toyama, Itsuko K.: 6120
Toyama, Masahiro: 6290
Toyokawa, Noriko: 6520
Toyokawa, Teru: 6520
Tracy, Elizabeth: 3115
Tracy, Eunjin Lee: 4682
Tracy, Karen: 5010, 6260
Trainum, Katie: 1570
Tran Levan, Darius: 4681
Tran, Candy: 9000
Tran, Elizabeth: 6570
Tran, Isabelle: 2080
Tran, Nathaniel: 1320
Tran, Sydney J.: 6580
Tran, Thanh: 6641
Tran, Yvette: 6760
Trappe, Scott: 9010
Trappe, Todd: 9010
Traunstein, Deb: 9010
Traver, Anthony C.: 2010, 3770
Travers, Jasmine: 1460, 2650, 3170, 4130

Travison, Thomas: 3110, 3600, 9030
Traywick, LaVona S.: 1420, 6740
Tremaine, Wendy: 4023
Trenary, Tanner T.: 9000
Trepanier, Jessica: 6100
Trinh, Mimi: 1570
Trinh-Shevrin, Chau: 1480
Trinkoff, Alison: 6560
Tripathi, Utkarsh: 6840
Trivedi, Ranak: 1330, 4010, 6310, 6430, 6530
Troutman, Brooke: 1080, 6630
Trowbridge, Kimberly S.: 6590
Truitt, William: 6220
Trujillo Tanner, Corinna M.: 6580, 6680
Trujillo, Miguel: 4430
Tsadok Cohen, Michal: 9020
Tsai, Pao-Feng: 6180
Tsai, Su-Ling: 6140
Tse, Dwight Cheuk Kit: 2480
Tse, Hin Wing (Florence): 4730, 6180
Tseng, Han-Yun (Heidi): 5110
Tseng, Tung-Sung: 4060
Tsoi, Yuet Yan: 3820
Tsugihashi, Yukio: 2580
Tsukada, Noriko: 3920, 4440, 6630
Tsumura, Hideyo: 9030
Tucker, Gretchen G.: 1250, 6790
Tucker, Julia E.: 1100, 6170
Tucker-Roghi, Gina C.: 2060
Tucker-Seeley, Reginald: 4510, 6540
Tuft, Samantha E.: 5083
Tunalilar, Ozcan: 3200, 4022, 6540
Tung, Yi-Hsuan: 1500, 6240
Turan, Bulent: 6590
Turfah, Ali: 6220
Turiano, Nicholas A.: 2140, 4620, 6550, 9010
Turkelson, Angela: 2440, 3480
Turner, Arlener D.: 6680
Turner, Kelly: 6760
Turner, Natalie: 3670
Turner, Rachael: 9020
Turner, Shelbie: 1080, 2280, 5000
Turney, Indira: 4470
Tu, Wanzhu: 1390, 6270
Tu, Xin: 6490
Twal, Riad: 1250
Tyler, Denise A.: 3640, 4350
Tyler, Janice: 4360
Tyrrell, Daniel: 3241

U

Ucar, Duygu: 3001
Udina Argilaga, Cristina: 6450
Udoh, Idorenyin Imoh: 9020
Ugiliweneza, Beatrice: 6110
Uittenhove, Kim: 6380, 6580
Ukraintseva, Svetlana: 1000, 1390, 4290, 4810, 6411, 9000
Ulbricht, Christine: 6770, 9010
Ullal, Tarini: 9010
Umberson, Debra J.: 4460, 6190, 9030
Um, Sun-Bi: 6810
Undem, Teri: 6780
Une, Saki: 6000
Ungar, Lyle: 6170
Ungar, Rachel: 6790
Unroe, Kathleen T.: 1390, 3170, 4100, 6760, 6770
Uppalapati, Naveen: 9020
Ursal, DJ: 4190
Utz, Rebecca L.: 4480, 4530, 6310, 9010
Uva, Katherine: 3780
Vélez Ortiz, Daniel: 5150

V

Vadean, Florin: 6700
Vaeli Zadeh, Ali: 3483, 4021, 6740, 9020, 9030
Valadares, Kevin J.: 6220
Valdes McClure, Nicole: 9030
Vale, Michael T.: 4730
Van Vleet, Samuel C.: 4684, 6050
van Achterberg, Theo: 1160, 2090, 9000
van Antwerp, Kodi: 6300
Van Bogart, Karina: 6290
Van Den Heever, Carmen: 4890
van der Beek, Allard: 9020
van der Kaaij, Serena: 4550
van der Steen, Jenny T.: 3555, 6270
van der Velpen, Isabelle F.: 5170
van der Wees, Philip: 1120
van der Willik, Odette: 2300
Van Haitsma, Kimberly: 2340, 2680, 3310, 3520, 4681, 6130, 6420
van Heumen, Lieke: 2050
Van Houtven, Courtney H.: 5083
van Leeuwen, Theo T.: 4550
Van Lierop, Merel (E.A.): 4720, 9010
Van Maanen, Rob: 9010
Van Puymbroeck, Marieke: 2280, 3930
van Rossum, Erik: 4720, 9010
Van Vleet, Bryce: 3480, 9020
Van Vught, Anneke: 4720
van Zyl, Carin: 6120
Vander Meulen, Maria E.: 9000
Vander Wyk, Brent: 5100
Vanderboom, Catherine E.: 3390
VanderWerf, Jessica A.: 6000, 6220
VandeWeerd, Carla: 6390
VanHaitsma, Kimberly: 4360
VanHoven, Miri: 6411
Vanias, Kaitlyn J.: 6530
VanSwearingen, Jessie: 3690
Varadaraj, Varshini: 3560
Varadhan, Ravi: 1010
Varela, Alberto: 2060
Varela, Antonio: 6740
Vargas, Laura X.: 4225, 6180
Vargas, Roberto: 9020
Varghese, Joshua: 4021
Varner Perez, Shelley: 6100
Varteresian, Taya: 3750
Vasan, Shradha: 6790
Vasiliadis, Helen-Maria: 6740
Vasquez, Elizabeth: 2170
Vazquez, Julia: 2700, 4225, 6180
Veal, Britney: 6290
Vecchione, Katia: 4030
Vedder, Anneke: 4460
Veeramah, Krishna: 4620
Vega, William: 6770
Veillet, Stanislas: 9010
Velasquez, Alfredo J.: 3440
Velazquez, Nathan: 4020
Velez, Bianca: 6790
Veliz, Phillip: 4690
Vemuri, Prashanthi: 6780
Vendittelli, Rebecca: 6290
Venema, Dawn: 6170
Venetto, Angelina: 4080
Vera Murillo, Lizbeth C.: 9000
Vera, Daniel: 1140
Vera, Renzo: 9000
Verbeek, Frank: 4720
Verbeek, Hilde: 1160, 2090, 3555, 4300, 6560
Verble, Danielle: 2390
Verdery, Ashton: 4070, 6300, 6310, 9020
Vergara, Lizandra G.: 1570, 6240
Verghese, Joe: 4340, 6780, 9020
Vernooij-Dassen, Myrra: 5170
Vernooij, Meike: 5170
Versey, H. Shellae: 3290, 5080, 6350
Verspoor, Eline: 5170
Vetter, Valentin M.: 1010, 6010, 6641
Vickers, Jasmine K.: 6110, 6590
Vickerstaff, Victoria: 3555
Victor, Christina: 2690
Viejo, Henry: 6650
Vien, Tracey L.: 9000
Vilar Fernandes, Lara: 9020
Villalba, Madeline: 9000
Villamor, Eduardo: 4905, 6790
Villamor, Monique V.: 9000
Villarreal, Francisco: 9010
Villeneuve, Evan: 3860
Vilpert, Sarah: 2680, 3720, 4480, 6550, 6730
Vincent, David: 3290
Virani, Salim: 4683
Virnig, Beth A.: 9010
Visaria, Abhijit: 2150
Visser, Geraldine: 4550
Vivek, Sithara: 6010
Viviano, Jeffrey: 6411
Vivoda, Jonathon: 6230
Voelkner, Abigail R.: 6130
Vogel, Carlyn E.: 3910, 4465, 4650
Vogel, David: 1140
Vogelsmeier, Amy: 3170, 4210
Vogle, Sophie: 6210
Vogt, Jax-Patrick: 6290
Voigt-Antons, Jan-Niklas: 6110
Voll, Stacey: 3440, 3510, 5084, 6440, 9020
Volpi, Elena: 4150, 6740
Von Ah, Diane: 2640
Von Gaudecker, Jane: 9030
von Gunten, Armin: 6380

von Humboldt, Sofia: 6180
Vonk, Jet: 4470
Vos, Eline: 9020
Voss, Lauren: 6080
Vu, Christine: 2080
Vu, Long: 6510
Vu, Nguyen: 6290, 6600
Vu, Thi: 4850, 9020
Vydiswaran, Vinod: 6060
Wöhrmann, Anne: 6700

**W**

Wacker, Michael: 5001
Wada, Tadashi: 4440
Wade, Gina: 4241
Wade, Kate: 4520
Wadhwa, Rohan: 6070
Wadsworth, Sally J.: 9000, 9010, 9020
Wagenknecht, Lynne: 9000
Wagner, Gert G.: 2470
Wagner, Jennifer: 6090
Wagster, Molly: 3080
Wahl, Hans-Werner: 2450, 2750, 5110
Waite, Linda J.: 1230, 2550
Wakefield, Dorothy: 1310, 2150
Wakita, Anna: 6180
Wakui, Tomoko: 3010, 6390, 6400
Walcott, Quinnlyn: 6120
Waldegrave, Charles: 2410, 3770
Waldinger, Robert J.: 2620
Waldron, Danielle: 9030
Waldrop, Deborah: 3930
Waldrop, Drenna: 4030
Walker, Jacob: 6220
Walker, Keenan: 9000
Walker, Leah: 6420
Walker, Ruth: 6640
Wallace, Andrea: 3500
Wallace, Cara L.: 6720
Wallace, Lauren A.: 2050
Wallace, Meredith L.: 2620, 4080
Wallace, Ramona: 9030
Wallace, Sarah: 6310
Waller, Dylan E.: 1040, 6420
Wallhagen, Margaret: 4050, 6460
Wall, Melanie W.: 3600
Wallsworth, Christine: 9010
Walsemann, Katrina M.: 1410
Walsh, Christine E.: 5060
Walsh, Sarah: 3160, 6000
Walston, Jeremy: 1010, 4680, 6411
Waltrip, Leah: 6260
Wan, Emily: 4470
Wang, Carly: 9000
Wang, Chen-pin: 6320
Wang, Claire: 4860, 6500
Wang, Cuiling: 6780
Wang, Danny: 1390, 2250, 6110, 6590, 9000
Wang, Dingyue (Demy): 9020
Wang, Ethan B.: 3560
Wang, Fei: 2200, 3630, 6410
Wang, Feng: 4461
Wang, Haowei: 6310, 9020
Wang, Hongning: 5090
Wang, Hongying: 6010
Wang, Huijun: 2080
Wang, James: 9000
Wang, Jiangxia: 3560
Wang, Jing: 1540, 4190, 1130, 4210, 6140, 6450, 3481
Wang, Jinjiao: 4580, 6140, 6270
Wang, Kaipeng: 1540, 4700, 5150
Wang, Kefang: 6760
Wang, Li: 6500
Wang, Lin: 1200
Wang, Lu: 4905, 6790
Wang, Mengya: 4510, 4905, 6790
Wangmo, Tenzin: 6400
Wang, Pengcheng: 6110
Wang, Qin: 6140
Wang, Qiong (Annabel): 4241
Wang, Sammy: 1130
Wang, Shaohung: 6170
Wang, Shuangshuang: 6530, 6720
Wang, Siqi: 2560
Wang, Song: 4700
Wang, Virginia: 5083
Wang, Weijun: 6280
Wang, Xiao (Joyce): 3200, 4820, 5081
Wang, Xiaochuan: 6090, 6350, 9020
Wang, Xinhui: 3510
Wang, Xueqing: 9030
Wang, Xuzhi: 9020
Wang, Yaqi: 6760
Wang, Yi: 1090, 6120, 6500
Wang, Yihan: 5030
Wang, Yiwen: 6290
Wang, YuHsuan (Olivia): 6720
Wang, Yuxia: 4681, 9030
Wang, Zhendong: 6150
Wanigatunga, Amal A.: 4120, 4330, 4600, 6740
Wanigatunga, Sarah K.: 4330
Wan, Qiaoqin: 6150
Wänström, Linda: 6440
Wan, Yihan: 6010
Waring, Molly E.: 6450
Warner, David F.: 6510
Warner, David: 6450
Warner, Nafisseh: 6450
Warren-Findlow, Jan: 2050
Warren, Keith: 4370
Warren, Rachel: 6460
Warren, Stacie: 6010
Washington, Faith-Christina: 2220, 9020, 9030
Washington, Karla T.: 6720, 6730
Washington, Tiffany R.: 2250, 2630
Wasil, Bridgette: 9030
Wastesson, Jonas: 6200
Watanabe, Kentaro: 6390, 6400
Watanabe, Yuichi: 9020
Waters, Leland: 1110, 2260, 6000, 9010
Watkinson, Erica: 2520
Watkins, Yashika: 6160
Watman, Deborah: 9030
Watson, Amanda A.: 6420
Watson, Wendy: 4022, 9020
Watt, Jennifer: 9000
Watts, Amber: 6190, 6570, 6650, 6730
Watts, Chelsea D.: 3230
Watts, Elizabeth E.: 3482
Waziry, Reem: 2241
Weaver, Ashley A.: 9000
Weaver, France: 3160
Weaver, Raven H.: 4900, 6610
Webb, Christopher: 2530
Webber, Anna: 6310
Webber, Katey: 9030
Webber, Sandra: 6780, 9010
Webber, Sophia: 6120
Webb, Fern J.: 6140
Webb, Nicole: 3550
Webster, Britney: 4060
Webster, Katelyn E.: 4320
Webster, Noah J.: 6310, 6490
Weech-Maldonado, Robert: 3660, 4820
Wee, Hwee lin: 6770
Weeks, Delaney: 2520
Weems, Jacy: 9000
Wehry, Susan: 3540, 6700, 9020
Weibel, Robert: 2450
Weidmann, Rebekka: 2280
Wei, Jeanne: 6130, 6180, 6840
Weil, Joyce: 3610
Wei, Melissa Y.: 2300
Weingrad, Zach: 5091
Weir, David R.: 3000
Wei, Sijia: 6280, 9030
Weiskittle, Rachel E.: 6220
Weissberger, Gali: 6480, 6580
Weiss, David: 2750
Welch, Sarah: 6270
Welle, Kevin: 5091
Wellens, Nathalie: 6270
Wendel-Hummell, Carrie L.: 2720
Wennberg, Alexandra: 1030
Werner, Nicole E.: 9000, 9010
Wesley, Alan: 5050
West, Brady: 3000
Westerhof, Gerben: 5110
Westerlind, Björn: 9020
West, Jessica S.: 5140
Westmore, Megan R.: 3000, 9030
Westra, Jordan: 9000
Westrick, Ashly C.: 1050, 2270, 4110
West, Stephanie T.: 3270
Wetherbee, Maya: 9000
Wethington, Elaine: 2300, 5150, 9010
Whalen, Laura: 2490
Wharton, Tracy C.: 6120
Wharton, Whitney: 2390
Wheaton, Felicia V.: 3260
Wheeler, David: 6070
Wheeler, Jason: 9020
Wheeler, Joseph E.: 9020
Wheeler, Kara: 6270
Wheeler, Katie: 9020
Whetung, Cliff: 5130, 6680
Whitbourne, Susan K.: 3800, 6000
White, Avalon: 6070, 6580, 6680
White, Carole L.: 2420, 4790
White, Elizabeth L.: 5081
Whitehead, Brenda: 6290
White, Justin: 6520
White, Katie: 2010, 3770
White, Kellee: 2300, 4730
White, Kina: 9030
White, Lon R.: 6411
White, Renee: 6720

White, Weyling: 3100
Whitfield, Keith E.: 2380, 3270, 4400, 6290
Whitmer, Rachel A.: 3600
Whitson, Heather: 2670, 4290, 4690
Whitten, Justin M.: 1280
Whitt-Glover, Melicia: 6180
Whitworth, Tanya: 1450
Wicker, Ellen: 6170, 6790
Wickramasinghe, Nilmini: 9030
Wickson-Griffiths, Abigail: 3555
Wickwire, Emerson: 6740
Widman, Christy A.: 6430
Wiechart, Kelly A.: 3540, 6070
Wiegelmann, Henrik: 5170
Wiemers, Emily E.: 1450
Wiemken, Timothy: 3600
Wiese, Lisa A.: 2210
Wieser, Heike: 1530
Wihry, David C.: 3540, 6270, 6540, 6700, 9030
Wikiera, John: 3000
Wilber, Kathleen: 3010, 4370, 4510, 6310, 9000
Wilckens, Max: 6700
Wilcock, Donna: 4870
Wilczek, Michael P.: 5084
Wilder, Jocelyn: 1230
Wiley, Jessica: 4520
Wilk, Christa: 6150, 6370, 6390
Wilkie, Rachel: 5130
Wilkinson, Anna V.: 1480
Wilkinson, Joanne: 6770
Wilks, Scott E.: 9020
Willard, Meredith: 4620, 6550, 9010
Willard, Tajalei: 9000
Willcox, Bradley J.: 3580, 6010
Willcox, D. Craig: 6010
Willems, Jules: 5100
Williams, Alice: 4150
Williams, Alicia R.: 6480
Williams, Brie: 2510, 5120
Williams, Dominique: 4681
Williams-Farrelly, Monica: 1410
Williams, Ishan C.: 1030, 2070, 2260, 6060
Williams, Kai: 2060
Williams, Karen: 3180
Williams, Kristine N.: 2520, 2530, 3240, 4030, 5010
Williams, Kyreon: 4160
Williamson, Jeff D.: 2530, 4150
Williams, Randi: 9000
Williams, Teresa: 6100
Williman, Martha R.: 3530
Willis, David: 6350
Willroth, Emily C.: 2140, 2620
Wills, Aria: 6270
Wills, Celia E.: 3360, 4650
Wilmoth, Janet M.: 2660
Wiloughby, Orion: 5091
Wilroth, Emily: 4620
Wilson, Deborah H.: 2200, 4680
Wilson, Edwina: 6420, 6780
Wilson, Jeffrey S.: 5080
Wilson, Kenneth A.: 6010, 6840
Wilson, Lara: 6350
Wilson-Lawson, Kimberly S.: 3360
Wilson, Loretta: 6100
Wilson, Lucas: 6310, 9030
Wilson, Lucy: 6270
Wilson, Lynn: 9020
Wilson, Megan E.: 2430
Wilson, Paige: 2530
Wilson, Stephanie J.: 3150, 4470, 5060
Wilson, Steven: 6700
Wilson, Traci L.: 2020
Wiltshire, Jacqueline C.: 9010
Windham, Beverly Gwen: 2670, 3110, 3600, 6780
Wingenfeld, Klaus: 9000
Wingo, Anna: 6720
Wingood, Gina: 3760
Winkler, Eva: 5110
Winship, Jodi: 3670
Winstead, Vicki: 6750
Winters-Stone, Kerri: 9030
Wise-Swanson, Breanne: 6610
Wishart, Dona J.: 4023
Wisk, Patricia G F.: 9030
Wissler Gerdes, Erin: 6840
Wister, Andrew: 6500, 6530
Witonsky, Kailyn: 9030
Witt, Dean D.: 2150
Wittenberg, Elaine: 9020
Witter, Julie: 6220
Witt, Paul: 4900
Witzel, Dakota D.: 5140, 6520, 6580
Wladkowski, Stephanie P.: 3930, 6000, 6720
Wojczynski, Mary K.: 4600, 4810, 9030
Wolfe, Alexander: 9010
Wolfe, Megan: 3120, 6350
Wolff, Jennifer: 4640, 4680
Wolf, Katie E.: 5150
Wolf, Michael: 1030
Wolf-Ostermann, Karin: 5170, 4300
Wolk, David A.: 6010
Womack, Jennifer L.: 3482
Won, Chang Won: 6450, 6510, 6690, 6740, 6780, 9020
Wong, Alan: 3483, 6740, 9030
Wong, Alyssa: 6110
Wong, Angel: 9030
Wong, Bob: 2310
Wong, Chelsea N.: 5084
Wong, Edwin: 9020
Wong, Edwin Lok Yan: 3481
Wong, Gloria H.Y.: 3481, 6110, 9020
Wong, Jen: 3270, 6750
Wong, Karen Lok Yi: 9000
Wong, Lily: 9000
Wong, Nicole HL: 3481
Wong, Rebeca: 2580, 4225, 6300, 6330, 6710, 9010, 9020
Wong, Rita: 9030
Wongsin, Utoomporn: 6330
Wongyara, Nattaya: 6770
Woodard, John L.: 6290, 6440, 6450
Woodard, LeChauncy: 3540
Wood, Elizabeth Geneva: 3870
Woodhouse, Mark: 6540
Wood, Leila: 5100
Wood, Rachel: 3670
Woods, Diana Lynn: 9030
Wood, Sinai: 5172
Woods, Robyn L.: 4320, 4683, 5083, 6320
Woods, Tonja: 3460
Woodward, Amanda T.: 4770
Woo, Jean: 3500
Woo, Jihee: 6090
Wooten, Karen: 3510
Working Group, Part: 6010
Worthington, Amber K.: 6270
Wretman, Christopher J.: 3910
Wright, Gus A.: 6010
Wright, Kathy D.: 3360, 4650, 5090
Wright, Kirsten: 1340, 9000
Wright, Regina S.: 2380, 6170
Wroblewski, Kristen E.: 1230, 6110
Wrosch, Carsten: 2620
Wrublowsky, Robert: 9030
Wrzus, Cornelia: 1020, 2470, 6590
Wu, Barry: 5172
Wu, Bei: 1080, 1130, 1540, 3115, 3481, 3870, 4240, 4500, 6140, 6450
Wu, Chao-Yi: 1340, 2450, 4790, 4870
Wu, Chenkai: 1000, 2170, 4040, 4320, 6200
Wu-Chung, E. Lim Lydia: 3481, 9010
Wu, Deqing: 6411
Wuestefeld, Paulina: 9030
Wuestney, Katherine: 6420
Wu, Heng: 6350
Wu, Jia-Rong: 3930
Wu, Kuan-Ching: 6420, 9000, 9030
Wu, Li-Xue: 4450
Wu, Lizi: 6580
Wu, Qiao: 2080
Wurm, Annika I.: 6050
Wurm, Susanne: 5110
Wurtz, Heather: 1160, 3660, 6610
Wu, Shih-Cyuan: 6540
Wu, Shinyi: 1500, 6240, 6750, 9030
Wu, Shwu-Chong: 6350
Wu, Tsung-Chin: 6490
Wu, Wenbo: 3130, 6060
Wu, Wenmiao: 6530
Wu, Yanyan: 6180
Wu, Ying-Jung: 6780
Wyatt-Brown, Anne M.: 4660
Wylie, Molly: 3320
Wyte-Lake, Tamar: 2660, 2720

### X

Xanthakis, Vanessa: 3400
Xavier, Rose Mary: 9000
Xiang, Lucille: 9030
Xiang, Qingyan: 2501
Xiang, Wang: 9010
Xiao, Chunhong: 6750
Xiao, Qian: 4560
Xiao, Yan: 6770
Xidous, Dimitra: 9010
Xie, Bo: 1570, 6150, 6260, 6730
Xie, Rui: 2290, 3840
Xie, Sharon: 6140
Xie, Yixia: 5001
Xiong, Yiyi: 9010
Xuanrong, Zhang: 9000
Xu, Dongjuan: 6760, 9030
Xue, Lingshu: 9030
Xu, Emily L.: 6530
Xue, Qian-Li: 1010, 3110, 4680

Xue, Tingzhong (Michelle): 9020, 9030
Xue, Wei-Lin: 6630
Xu, Hanzhang: 1130, 1540, 6450
Xu, Hongwei: 3010
Xu, Jiayun: 9030
Xu, Lili: 3950, 6360
Xu, Ling: 1080, 6060, 6630
Xu, Mengyu: 9020
Xu, Qi: 6350
Xu, Qingwen: 6230
Xu, Shu: 3100, 6090, 6450, 6510
Xu, Wenyao: 6280
Xu, Yanfeng: 3740, 9030
Xu, Yunpeng: 9010

**Y**

Yadav, Hariom: 9000, 9010
Yadav, Uday Narayan: 6750
Yaffe, Kristine: 1430, 2670, 3560, 4560, 5030, 6110
Yahr, Andrea: 6530
Yakong, Vida N.: 1420
Yamaga, Ryonosuke: 6240
Yamaguchi, Yuko: 9000
Yamakawa, Miyae: 6110, 6450, 6770, 9030
Yamamoto, Hiroshi: 6450
Yamamoto-Mitani, Noriko: 4440
Yamamoto, Ryohei: 6110, 6450, 6770
Yamanaka, Takashi: 6240
Yamashita, Takashi: 4555, 6130, 6520, 6670, 6710
Yamazaki, Sachiko: 6580
Yam, Hawking: 1480, 6540
Yancura, Loriena: 3740
Yan, Elsie: 4090, 9020
Yang, Bo K.: 9000
Yang, Eunjin: 6390, 6460, 6650, 9010
Yang, Feng: 4023
Yang, Iris: 5060
Yang, Jiue-An: 2450
Yang, Kevin H.: 9000
Yang, Laiji: 3600
Yang, Luyan: 1500
Yang, Mia: 4150
Yang, Na: 1200
Yang, Rumei: 4682
Yang, Wei: 3870
Yang, Yang C.: 3115, 5060
Yang, Yulin: 6200
Yan, Jinjin: 4730
Yankeelov, Pamela: 1110
Yan, Mengzhao: 3900, 4370
Yannatos, Isabel: 6140
Yao, Jacqueline: 6010
Yao, Nengliang: 6530, 6720
Yaqub, Amber: 5170
Yashin, Anatoliy: 1000, 1390, 4290, 6010, 6110, 6411, 9000
Yashkin, Arseniy: 1000, 1390, 4290, 6110, 9000
Yata, Vinod K.: 9010
Yauch, John: 2590
Yau, Jessie Ho-Yin: 3481
Yauk, Jessica: 4650, 6290
Yau, Roger: 9030
Yeager, Brooke: 9020
Yeager, Valerie: 6760
Yee, Claire: 4681, 4850
Yefimova, Maria: 1390
Yeh, An-Yun: 4700
Yeh, Charlotte: 6170, 6580, 6790
Yeh, Jarmin C.: 3290, 5080, 9030
Yellseagle, Debara: 1060
Yelvington, Miranda: 9000
Ye, Minzhi: 2640
Yentes, Jennifer: 6170
Yeo, Hyesu: 6700, 6720
Yeow, Adrian: 4220
Yeung, Dannii: 3630, 6390
Ye, Wei: 6700
Yi, Eun-Hye G.: 6120
Yi, Julie: 4680
Yin, Cheng: 1090, 3540
Yin, Michael: 9000
Yi, Stella S.: 1480
Ylitalo, Kelly: 6510, 6650
Yoder, Karmen: 6220
Yokoyama, Yuri: 6320, 6710
Yonashiro-Cho, Jeanine: 3010
Yoneda, Tomiko: 2140, 6290
Yon, Yongjie: 9000
Yoo-Jeong, Moka: 4905, 6790
Yoo, Ji Won: 6420
Yoon, Hyun Woo: 3160
Yoon, Joonsik: 1450
Yoon, Jung Min: 6560
Yoon, Si On: 3830
Yoon, YeaSeul: 6270
Yoo, Subin: 6280
Yoquinto, Lucas: 3450
Yorgason, Jeremy B.: 2660, 6070, 6580, 6680
Yoshikawa, Aya: 6310
Yoshinaga, Shushi: 2680
Yoshino Benavente, Julia: 1030
Yost, Elizabeth: 6220
Young, Danielle: 9010
Young, Heather: 2680
Young, Kim: 3550
Young, Yuchi: 6090, 6720
Yousefzadeh, Matt: 2501, 3241, 6840
You, Suha: 6340
You, Tongjian: 1280, 9010
Yow, W. Quin: 2290, 3250, 3840, 6210, 6411
Yuan, 3. Jessie: 5173
Yuan, Fengpei: 9000
Yuan, Jing: 4640
Yuan, Keming: 4100
Yuan, Shuhan: 9020
Yuan, Yiyang: 6770, 9010, 9020
Yue, Lirong: 6530
Yu, Fang: 4580, 6270, 9000
Yu, Hsiao-Wei: 9010
Yu, Jiao: 3260, 6730
Yu, Kexin: 1500, 2570, 4870
Yu, Lei: 4820
Yung, Raymond: 6470
Yun, Ji-Young: 3330
Yun, Soo R.: 3810
Yu, Wanting: 3510, 9010, 9030
Yu, Wenshan: 1410
Yu, Xinfang: 6620
Yu, Xuexin: 2270, 3040

**Z**

Zaccaria, Daniele: 6380
Zachgo, Ashlyn: 9030
Zadeh, Rana: 9010
Žádník, Štěpán: 1210
Zafar, Anum: 4500
Zafar, Mateeha: 6060
Zaheed, Afsara: 1240
Zahodne, Laura B.: 1240, 1410, 2360, 3510, 4470, 4700
Zajdel, Melissa: 6170
Zakoul, Heidi: 6641
Zakrajsek, Andrea G.: 6000
Zakrajsek, Jennifer S.: 3000, 6470
Zakrzewski, Chris: 5172
Zambrano Garza, Elizabeth: 6210, 6390
Zammit, Andrea R.: 4700
Zamora, Ezequiel: 9030
Zamrini, Edward: 5173
Zanetti, Mariella: 5010
Zang, Emma: 1540
Zang, Ying: 1160
Zanier, Nicole: 3000, 6470
Zaninotto, Paola: 3210
Zanjani, Faika A.: 2510, 6140, 9010
Zannas, Anthony: 3440
Zanville, Noah: 9020
Zanwar, Preeti: 1060, 2640, 3870
Zúñiga, Franziska: 1530, 2090, 6270, 9000
Zaragoza, Gabriela: 4510, 9000
Zarit, Steven H.: 1450
Zarow, Chris: 6411
Zarowitz, Barbara J.: 3650
Zaslavsky, Oleg: 6420, 9000, 9030
Zausniewski, Jaclene: 3115, 5140, 6090, 6820
Zavala, Daisy V.: 4620
Zazdravnykh, Evguenii: 1060
Zazueta-Borboa, Jesús-Daniel Z.: 6330
Zeki Al Hazzouri, Adina: 9010
Zelin, Alexandra: 6640
Zelinski, Elizabeth M.: 5000, 6440
Zell, Angela: 2210, 9020
Zeng, Qing: 6770
Zhai, Wanting: 3400
Zhang, Alice: 6650
Zhang, Amanda: 1230
Zhang, Bing: 2080
Zhang, Bo: 4620
Zhang, Fan: 9000
Zhang, Hongqiao: 9020
Zhang, Jackie: 9020
Zhang, Jingwen: 9020
Zhang, Jingyun: 6411
Zhang, Keqing: 1080
Zhang, Lei: 2501, 6840
Zhang, Lingling: 2080
Zhang, Meng: 6530
Zhang, Ming: 4700
Zhang, Mutian: 1500, 3390, 6540
Zhang, Nan: 9020
Zhang, Peiyuan: 1390, 6730, 9000
Zhang, Shenghao: 1570, 6290
Zhang, Shifang: 6150
Zhang, Shiyang: 1450, 6460
Zhang, Tan: 6641
Zhang, Wei: 1080, 1100
Zhang, Weiwei: 4691
Zhang, Wen: 9020
Zhang, Xiaomin: 6840
Zhang, Xiaoyan: 6790
Zhang, Xinyao: 2080
Zhang, Xu: 3241
Zhang, Yalu: 9010
Zhang, Yan: 2400, 4730, 9000
Zhang, Yaxin: 4683, 9020
Zhang, Ye: 9000, 9010
Zhang, Yingyang: 6110

Zhang, Yuan S.: 2270, 3040, 9020
Zhang, Yuankai: 9020
Zhang, Yun: 3440, 3510
Zhang, Yuteng: 5001
zhang, Zhenmei: 3010
Zhang, Ziying: 6500
Zhan, Rong-Fang: 1090
Zhan, Yan: 9020
Zhao, Dandan: 2080
Zhao, Duzhi: 4530, 5070
Zhao, Erfei: 5000
Zhao, Hui: 1030, 9000
Zhao, Xiaopeng: 1250, 9000
Zhao, Xiaoyan: 6150
Zhao, Yuxin: 6750
Zhao, Zixuan: 4700
Zha, Peijia: 4700
Zheng, Anqing: 9020
Zhong, Jie: 3060
Zhong, Lily: 2150
Zhong, Xin: 4023, 9030
Zhong, Zi-Ting: 6540
Zhou, Gang: 6420
Zhou, Juan Helen: 6411
Zhou, Junhong: 3482, 3510, 4040, 9010
Zhou, Rui: 6720
Zhou, Shuai: 1400, 3820
Zhou, Weijiao: 4320
Zhou, Yuanjin: 1150, 4010, 6260
Zhou, Zehui L.: 4680
Zhou, Zexi: 1450, 6170
Zhou, Zeyu: 1470
Zhu, Huanzhi: 6140, 6450
Zhu, Jingmin: 3210
Zhu, Shanshan: 6760
Zhu, Shijun: 5171
Zhu, Xiaonan: 3250, 3690, 4340, 9030
Zhu, Xiaoqian: 2670
Zhu, Yating: 4430
Zhu, Yi: 6840
Zhu, Yujun: 4100
Zhu, Zheng: 3481
Ziade, Garnelle: 6380
Zigman Suchsland, Monica: 5010
Zilcha-Mano, Sigal: 3600
Zilioli, Samuele: 6200, 6290
Zimmerman, Kristin M.: 2260
Zimmerman, Sheryl: 3910, 4681
Zingmark, Magnus: 4220
Zionts, Nancy B.: 9030
Zisberg, Anna: 1160, 2680
Zmora, Rachel: 4060
Zmuda, Joseph M.: 3400, 4810, 6450
Zohn, Jennifer: 6770
Zonderman, Alan B.: 5060
Zou, Ning: 6150
Zubatsky, Max: 6790, 9000
Zubkoff, Lisa: 9010
Zucchero, Renee' A.: 4750
Zullo, Andrew: 4580
Zuoting, Nie: 4682
Zuraw, Matthew: 9010
Zurlo, Karen A.: 4380
Zu, Zhongliang: 4461
Zwakhalen, Sandra M.: 4720, 6270, 9010
